# ﻿Thirty novel fungal lineages: formal description based on environmental samples and DNA

**DOI:** 10.3897/mycokeys.124.161674

**Published:** 2025-10-20

**Authors:** Leho Tedersoo, Mahdieh S. Hosseyni Moghadam, Kristel Panksep, Victoria Prins, Sten Anslan, Vladimir Mikryukov, Mohammad Bahram, Kessy Abarenkov, Urmas Kõljalg, Keyvan Esmaeilzadeh-Salestani, Julia Pawłowska, Christian Wurzbacher, Yi Ding, Saad Hussin Alkahtani, R. Henrik Nilsson

**Affiliations:** 1 Mycology and Microbiology Center, University of Tartu, Liivi 2, 50409 Tartu, Estonia; 2 Department of Zoology, College of Science, King Saud University, P.O. Box 54234, 5294 Riyadh, Saudi Arabia; 3 Institute of Ecology and Earth Sciences, University of Tartu, Liivi 2, Tartu 50400, Estonia; 4 Chair of Hydrobiology and Fishery, Estonian University of Life Sciences, Kreutzwaldi 5, Tartu 51006, Estonia; 5 Institute of Technology, University of Tartu, Nooruse 1, 50411 Tartu, Estonia; 6 Department of Aquatic Resources, Swedish University of Agricultural Sciences, Ulls gränd 1, 756 51 Uppsala, Sweden; 7 Department of Biological and Environmental Science, University of Jyväskylä, Survontie 9, 40500 Jyväskylä, Finland; 8 Department of Biology, College of Science, Princess Nourah bint Abdulrahman University, Saudi Arabia; 9 Department of Agroecology, Aarhus University, Slagelse, Denmark; 10 Department of Ecology, Swedish University of Agricultural Sciences, Ulls väg 16, 756 51 Uppsala, Sweden; 11 Faculty of Biology, Institute of Evolutionary Biology, University of Warsaw, ul. Zwirki i Wigury 101, 02-089 Warsaw, Poland; 12 Chair of Urban Water Systems Engineering, Technical University of Munich, Am Coulombwall 3, 85748 Garching, Germany; 13 State Key Laboratory of Plant Diversity and Specialty Crops, Wuhan Botanical Garden, Chinese Academy of Sciences, Wuhan 430074, China; 14 Department of Biological and Environmental Sciences, Gothenburg Global Biodiversity Center, University of Gothenburg, Box 463, 405 30 Gothenburg, Sweden

**Keywords:** Dark taxa, DNA-based taxonomy, early-diverging fungal lineages, higher-level classification, legitype, nucleotype, phylogeny of fungi

## Abstract

Molecular analyses of soil and water commonly reveal large proportions of fungal taxa that cannot be assigned to any taxonomic or functional groups. Some of these so-called dark taxa have been encoded alphanumerically, while others have remained completely overlooked. Using long-read sequencing that covers much of the ribosomal RNA operon, we shed light on the phylogenetic and ecological distribution of fungal dark taxa and formally describe 30 of the most prominent phylum- to order-level lineages based on their characteristic DNA features. This increases the known large-scale fungal phylogenetic diversity by roughly one-third. Formal names will enhance taxonomic reproducibility, facilitate communication among researchers, and enable the estimation of conservation and quarantine needs for uncultivable species and higher-ranking taxa. The new species in the respective highest-level novel taxonomic groups include *Pantelleria saittana* (Pantelleriomycetes), *Paraspizellomyces parrentiae* (Paraspizellomycetales), *Aquieurochytrium lacustre* (Aquieurochytriomycetes), *Edaphochytrium valuojaense* (Edaphochytriomycetes), *Tibetochytrium taylorii* (Tibetochytriomycetes), *Tropicochytrium toronegroense* (Tropicochytriomycetes), *Algovorax scenedesmi* (comb. nov.) and *Solivorax pantropicus* (Algovoracomycetes), *Aquamastix sanduskyensis* (Aquamastigomycetes), *Cantoromastix holarctica* (Cantoromastigomycetes), *Dobrisimastix vlkii* (Dobrisimastigomycetes), *Palomastix lacustris* (Palomastigomycetes), *Sedimentomastix tueriensis* (Sedimentomastigomycetes), *Terrincola waldropii* (Terrincolales), *Curlevskia holarctica* (Curlevskiomycota), *Mycosocceria estonica* (Mycosocceriales), *Maerjamyces jumpponenii* (Maerjamycetes), *Ruderalia cosmopolita* (Ruderaliomycetes), *Bryolpidium mundanum* (Bryolpidiomycetes), *Chthonolpidium enigmatum* (Chthonolpidiomycetes), *Savannolpidium raadiense* (Savannolpidiomycetes), *Gelotisporidium boreale* (Gelotisporidiomycetes), *Sumavosporidium sylvestre* (Sumavosporidiomycetes), *Parakickxella borikenica* (Parakickxellomycetes), *Aldinomyces tarquinii* (Aldinomycota), *Borikenia urbinae* (Borikeniomycota), *Mirabilomyces abrukanus* (Mirabilomycota), *Nematovomyces vermicola* (comb. nov.) and *N. soinasteënsis* (Nematovomycota), *Viljandia globalis* (Viljandiomycota), *Waitukubulimyces cliftonii* (Waitukubulimycota), and *Tartumyces setoi* (Tartumyceta).

## ﻿Introduction

Fungi are tremendously diverse regarding species numbers and evolutionary divergence (Tedersoo et al. 2018; Voigt et al. 2021; Niskanen et al. 2023; Quandt et al. 2023; Seto et al. 2023). Despite their fundamental biological roles and importance in ecosystem functioning, the taxonomy and classification of fungi lag far behind those of macroscopic plants and animals. This disparity is related to their microscopic size, challenges in isolation, establishing pure cultures, and the relatively limited number of taxonomists specializing in this area (Niskanen et al. 2023; Seto et al. 2023). Advances in nucleic acid sequencing technologies have revolutionized our understanding of fungal diversity, revealing previously unrecognized lineages and shedding light on their ecological roles (Tedersoo et al. 2017; Ahrendt et al. 2018; Galindo et al. 2021; Seto et al. 2023).

Unlike prokaryotes, which can be described solely based on nearly full-length genome sequences according to SeqCode (Hedlund et al. 2022), the solutions for DNA-based taxonomy remain poorly regulated for hitherto uncultured and unobserved fungi by the International Code of Nomenclature (ICN) for algae, fungi, and plants (ICNAFP; Turland et al. 2025) and the ICN for animals (ICZN), which handles microsporidia (International Commission on Zoological Nomenclature 1999). For example, it remains unclear to what extent voucher specimens and samples may contain other organisms (e.g., lichenized fungi) and whether DNA, as a part of an organism, can be used as type material.

In the research and practitioner communities, there is a high demand for communicating fungal taxa (Ryberg and Nilsson 2018; Kõljalg et al. 2020). In particular, identifying and categorizing species into higher-ranking taxonomic groups is essential for understanding the biodiversity and functioning of the surrounding microbiome, as well as for incorporating them into legislative frameworks (Lücking et al. 2021; Nilsson et al. 2023). The absence of a universally accepted framework for naming limits the potential for consistent identification and comparison of these organisms across studies and ecosystems (Nilsson et al. 2023).

In mycology, higher-ranking taxonomic groups of previously undescribed lineages (PULs) are often indicated by non-standardized alphanumeric identifiers or other types of informal names. These names typically vary across research groups, which complicates data synthesis and across-study comparisons (Arroyo et al. 2018; Kõljalg et al. 2020; Tedersoo et al. 2024). Some of the order-to-phylum-level PULs within or close to the fungal kingdom were, as a rule, evinced from relatively short, sometimes non-overlapping marker gene fragments from specific habitats (Tedersoo et al. 2017; Arroyo et al. 2018; Quandt et al. 2023). Here, we combine a long-read, high-throughput sequencing approach of soil and sediment samples with recently released long-read data in public databases to accommodate the PULs into the fungal tree of life. Our main objective is to characterize and formally describe these enigmatic fungal lineages based on voucher samples and eDNA, long-read sequences, and phylogenetic analyses to progress towards completion of the fungal tree of life.

## ﻿Materials and methods

We downloaded the sequence data identified to any fungal phylum (but not to class or lower taxonomic levels), unspecified fungi, or unspecified eukaryotes from three nucleotide sequence databases: NCBI (https://www.ncbi.nlm.nih.gov/), UNITE v9.1 (Abarenkov et al. 2024; https://unite.ut.ee/), and EUKARYOME v1.9.2 (Tedersoo et al. 2024; https://eukaryome.org/). These sequences were first assigned to rough taxonomic groups based on BLASTn queries against identified sequences in EUKARYOME. Within these groups, long reads containing > 1000 bp of the 18S rRNA gene (SSU) and/or > 1000 bp of the 28S rRNA gene (LSU) were selected for preliminary phylogenetic analyses using long-read reference sequences from all eukaryotic phyla. The sequences were aligned using MAFFT v7 (Katoh and Standley 2013), followed by manual trimming of overarching and misaligned ends, trimming of introns in internal transcribed spacer (ITS) regions, and manual correction in case of apparent misalignments using AliView v1.26 (Larsson 2014). The alignments were processed in ClipKIT v1.4.0 (Steenwyk et al. 2020) to remove phylogenetically uninformative positions. Preliminary phylogenetic analyses were performed using IQ-TREE v2.2.5 (Minh et al. 2020), including 1000 trees and 1000 ultrafast bootstrap replicates. The trees were visualized in FigTree v1.4.4 (Rambaut 2018) for phylogeny-guided taxonomic regrouping of unknown fungi into PULs. During this process, we marked and removed low-quality and chimeric reads (around 10% of all reads). We then performed more focused phylogenetic analyses for the fungal kingdom by adding at least one representative sequence per order (or class in Basidiomycota and Ascomycota) as described above and keeping representative sequences of Choanoflagellozoa and Nucleariae, a sister group of fungi, as an outgroup.

Based on fungal phylogenies, we removed reads from any unknown fungi that formed ultra-long branches but had no evidence of quality issues. Due to their long branches and destabilizing effect on the phylogenetic estimates, we also removed representatives of Microsporidia, Caulochytriales, Nephridiophagales, Dimargaritales, Asellariales, and Coelomomycetales after ensuring that these groups affiliate with none of the PULs. From each taxonomic group of PULs, we kept the longest reads characteristic of sublineages to avoid oversizing the phylograms. For the final phylogenetic analysis, we added a few shorter reads by using the MAFFT “align” option if these were deemed important for genus-level taxonomic interpretation of the PULs (i.e., delimiting new genera). To keep the main focus at the phylum level, we excluded most class-level PULs of Rozellomycota (syn. Cryptomycota), Ascomycota, and Basidiomycota, all of which warrant separate analyses of similar magnitude. We only handled two large PULs of Rozellomycota that were placed in other phylogenetic positions based on previous analyses using much smaller datasets (clades GS01 and GS15 in Tedersoo et al. 2017).

For each fungal PUL, we prepared additional separate phylogenetic analyses by compiling all existing sequence data and associated geographical and ecological metadata (as of 30 September 2024) in NCBI, UNITE, EUKARYOME, the Global Soil Mycobiome Consortium (GSMc) project (Tedersoo et al. 2021), and the FunAqua project (water and sediment samples; https://sisu.ut.ee/funaqua/). We also queried representative sequences of PULs against GlobalFungi v5.0 (Vetrovsky et al. 2020) to obtain additional sequences and associated information on the distribution of these species based on previous short-read amplicon studies. For inclusive phylogenetic analysis of each PUL, a few representatives of sister taxa, determined based on the main analysis, were selected as an outgroup. Reads with compromised quality were excluded based on MAFFT alignments covering the SSU, ITS, and LSU regions and preliminary phylograms. We determined the potential type species and assignment of terminal taxa into genera and higher-level taxonomic groups based on the final alignments and phylograms. Potential type species were selected considering i) their commonness, ii) representation of the entire PUL (i.e., assignment to one of the main sublineages), iii) representation by at least one ultra-long read with an available DNA sample and rich metadata, and iv) unequivocal distinction from closely related species based on the ITS region. The ITS region is the formal fungal DNA barcode (Schoch et al. 2012) and the most broadly used marker for species-level identification (Nilsson et al. 2019).

Diagnoses of species were prepared based on molecular characters in the ITS and LSU regions by selecting the most characteristic short, unique barcodes of typically 20 bases that we refer to as diagnostic nucleotide signatures (Labeda et al. 2001) for the target species based on the PUL-specific alignments. Within the ITS region, we specifically focused on ITS2 because of more available sequence data and fewer homopolymers that give rise to sequencing errors. The diagnostic nucleotide signatures were required to have a minimum number of ambiguous positions for the target species and a maximum number of mismatches to closely related species. Based on the alignments, we estimated the number of allowed mutations for the target species to be distinguishable from any related species. For the entire alignment length of ITS2 and LSU, we estimated intraspecific sequence variability based on the maximum proportion of differences among individual reads. For the LSU, characteristic barcodes and intraspecific variability are less clear due to smaller read coverage and the lower number of species available for comparison. Similar but typically shorter diagnostic nucleotide signatures were determined for genera and higher-ranking taxa. The positions of diagnostic nucleotide signatures refer to the positions of the ex-holotype sequence for the ITS region and additionally to *Saccharomyces cerevisiae* reference strain S288C accession NR_132207 (locus tag YNCL0010C) for SSU (NR_132213), 5.8S (NR_132211), and LSU (NR_132209). We used the information on ITS intraspecific variability of the type species for clustering and manual assessment of phylograms to produce rough estimates of potential species richness at the levels of PUL and genus. No attempt was made to extrapolate to unsampled species.

The vouchered physical samples that served as a basis for long-read sequence data were selected as holotypes to describe the type species, following Kirk and Griffith (2021). These vouchered-type samples, along with the extracted and vouchered eDNA samples, are deposited in the repository of the University of Tartu (acronym TUE, with 6-digit accession numbers) unless mentioned otherwise. We propose the term “nucleotype” (*nucleotypus* in Latin) to stand for these ex-type DNA samples, which under certain circumstances (e.g., when the holotype is lost) could also be used as types (Tedersoo et al. 2025). We also propose the term “legitype” (*legitypus* in Latin) to denote the main holotype-derived DNA sequence that is used to represent the species. Legitypes constitute amplicon-derived DNA barcodes or whole-genome sequences that are intended to act as ex-type DNA sequences or as types (for example, when the holotype and nucleotype are lost, or when the research community decides to shift to sequence-based typification). Here, we determined legitypes among the longest and highest-quality sequences derived from the nucleotype of preserved physical voucher samples. Most of the holotypes and underlying environmental samples used for typification originate from composite topsoil samples of the GSMc project, water and sediment samples of the FunAqua project, and various material samples sequenced by Jamy et al. (2022). These samples are accordingly referred to in the species descriptions and their voucher information in TUE. Legitype and other DNA sequences with metadata were first deposited in the EUKARYOME database (denoted by “EUK” with 7-digit accession numbers) and subsequently submitted to the UNITE and European Nucleotide Archive (accessions OZ253786–OZ253832) databases.

For the naming of taxa, all co-authors were invited to propose names separately for species epithets and genera, including justification and etymology. Based on the geographical and ecological metadata and collector information of type material and additional samples, co-authors were instructed to propose characteristic names by favoring local languages but avoiding names of co-authors and those potentially insulting or related to the dominant plant species. All proposed names were checked for homonymy and spelling correctness. Then, all co-authors voted for the most suitable names; in case of equal votes, the first author decided on the final name. Such naming conventions are common in mycology, although adjectives prevail in classical names described based on culture or fruiting body specimens or illustrations (Smith 2024). Apart from typification, species descriptions follow the best practices for fungi (Aime et al. 2021).

For establishing higher-ranking taxa, such as genera, families, orders, classes, phyla, and subkingdoms, we used the following criteria: i) monophyly; ii) bootstrap support > 95; iii) phylogenetic breadth and divergence roughly comparable to previously described taxa; and iv) minimizing the number of novel taxa (i.e., preferably retaining larger groups if there were multiple alternative splitting possibilities). Names of higher taxa were derived from generic names.

## ﻿Results and discussion

### ﻿Novel fungal lineages

**Figure 1.**
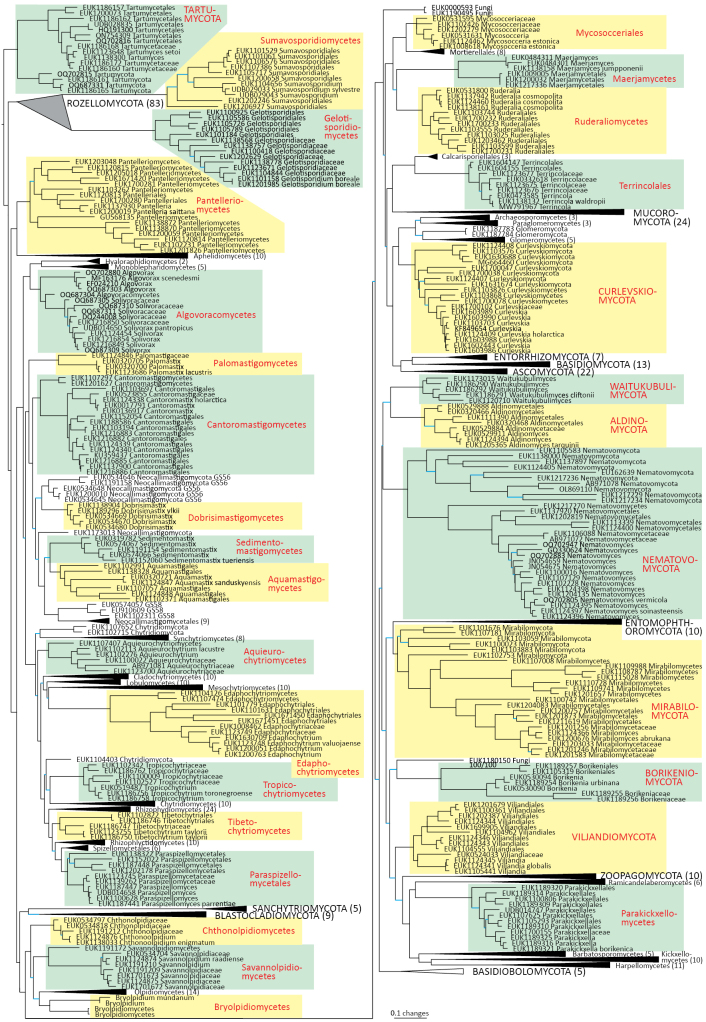
Maximum Likelihood SSU-5.8S-LSU phylogram indicating placement of novel lineages in the fungal kingdom as highlighted in a coloured shade and red font. Previously described taxonomic clades are collapsed. All branches of Sumavosporidiomycetes and Gelotisporidiomycetes are shortened two-fold. Poorly supported branches with bootstrap values below 95%/99% are indicated in blue. The original tree with bootstrap values and uncollapsed branches is indicated in Suppl. material 1.

Maximum likelihood phylogenetic analyses of long-read rRNA markers across eukaryotes and within fungi grouped the PULs into 30 coherent, monophyletic taxonomic groups within the fungal kingdom (Table 1, Fig. 1, Suppl. material 1: fig. S1). Six of these groups correspond to previously noted distinct fungal lineages with alphanumeric identifiers (Tedersoo et al. 2017; Arroyo et al. 2018; Seto et al. 2023). Others constitute entirely novel taxa, although searches against nucleotide sequence databases usually reveal strong matches to previous entries of environmental sequences produced by cloning and Sanger sequencing (Waldrop et al. 2006; Curlevski et al. 2014) or various high-throughput sequencing technologies (Anderson et al. 2018; Eshghi Sahraei et al. 2022). Based on a conservative taxonomic approach, eight of these PULs correspond to novel phyla and add significantly to the hitherto recognized phylogenetic diversity of fungi (11–18 phyla, depending on classification; Tedersoo et al. 2018; James et al. 2020; Hyde et al. 2024). Outside these novel phyla, we describe and delimit 22 well-supported class- or order-level groups in the non-Dikarya. In total, we describe 29 new species, 31 genera, 31 families, 31 orders, 27 classes, and 8 phyla, and propose two new taxonomic combinations (Table 1; see below). The fungal taxonomic table, updated from the Outline of Fungi (Hyde et al. 2024), is provided as Suppl. material 2.

**Table 1.** Newly proposed phyla, classes, orders, genera and species.

Phylum	Class	Order	Genus and species
Aldinomycota	Aldinomycetes	Aldinomycetales	Aldinomyces tarquinii
Aphelidiomycota ^1^	Pantelleriomycetes	Pantelleriales	Pantelleria saittana
Borikeniomycota	Borikeniomycetes	Borikeniales	Borikenia urbinana
Calcarisporiellomycota ^1^	Calcarisporiellomycetes ^2^	Terrincolales	Terrincola waldropii
Chytridiomycota ^1^	Aquaeurochytriomycetes	Aquaeurochytriales	Aquieurochytrium lacustre
Chytridiomycota ^1^	Edaphochytriomycetes	Edaphochytriales	Edaphochytrium valuojaense
Chytridiomycota ^1^	Spizellomycetes ^2^	Paraspizellomycetales	Paraspizellomyces parrentiae
Chytridiomycota ^1^	Tibetochytriomycetes	Tibetochytriales	Tibetochytrium taylorii
Chytridiomycota ^1^	Tropicochytriomycetes	Tropicochytriales	Tropicochytrium toronegroense
Curlevskiomycota	Curlevskiomycetes	Curlevskiales	Curlevskia holarctica
Kickxellomycota ^1^	Parakickxellomycetes	Parakickxellales	Parakickxella borikenica
Mirabilomycota	Mirabilomycetes	Mirabilomycetales	Mirabilomyces abrukanus
Monoblepharomycota ^1^	Algovoracomycetes	Algovoracales	Algovorax scenedesmi
Monoblepharomycota ^1^	Algovoracomycetes	Solivoracales	Solivorax pantropicus
Mortierellomycota ^1^	Maerjamycetes	Maerjamycetales	Maerjamyces jumpponenii
Mortierellomycota ^1^	Mortierellomycetes ^2^	Mycosocceriales	Mycosocceria estonica
Mortierellomycota ^1^	Ruderaliomycetes	Ruderaliales	Ruderalia cosmopolita
Nematovomycota	Nematovomycetes	Nematovomycetales	Nematovomyces soinasteënsis^3^
Neocallimastigomycota ^1^	Aquamastigomycetes	Aquamastigales	Aquamastix sanduskyensis
Neocallimastigomycota ^1^	Cantoromastigomycetes	Cantoromastigales	Cantoromastix holarctica
Neocallimastigomycota ^1^	Dobrisimastigomycetes	Dobrisimastigales	Dobrisimastix vlkii
Neocallimastigomycota ^1^	Palomastigomycetes	Palomastigales	Palomastix lacustris
Neocallimastigomycota ^1^	Sedimentomastigomycetes	Sedimentomastigales	Sedimentomastix tueriensis
Olpidiomycota ^1^	Bryolpidiomycetes	Bryolpidiales	Bryolpidium mundanum
Olpidiomycota ^1^	Chthonolpidiomycetes	Chthonolpidiales	Chthonolpidium enigmatum
Olpidiomycota ^1^	Savannolpidiomycetes	Savannolpidiales	Savannolpidium raadiense
Rozellomycota ^1^	Gelotisporidiomycetes	Gelotisporidiales	Gelotisporidium boreale
Rozellomycota ^1^	Sumavosporidiomycetes	Sumavosporidiales	Sumavosporidium sylvestre
Tartumycota	Tartumycetes	Tartumycetales	Tartumyces setoi
Waitukubulimycota	Waitukubulimycetes	Waitukubulimycetales	Waitukubulimyces cliftonii
Viljandiomycota	Viljandiomycetes	Viljandiales	Viljandia globalis

^1^ Previously described phylum ^2^ Previously described class ^3^ There is additionally a new combination, *N. vermicola*.

Most PULs were detected mainly from soil, except Aquieurochytriomycetes, Aquamastigomycetes, Palomastigomycetes, and Sedimentomastigomycetes, which were more frequent in freshwater or sediment samples (Suppl. material 3). This may be related to the fact that soil is the principal habitat for most fungal species (Anthony et al. 2023). A majority of PULs featured terrestrial as well as aquatic fungi, suggesting the occurrence of multiple habitat transitions in these groups as commonly observed in other fungi and eukaryotes (Jamy et al. 2022). Nearly all PULs had a broad distribution across multiple biomes and continents, indicating low endemicity at higher taxonomic levels. Borikeniomycota and Tropicochytriomycetes were globally distributed but displayed most records in neotropical habitats.

We estimate that the present PULs comprise from five species (Maerjamycetes, Sedimentomastigomycetes, and Tibetochytriomycetes) to around 1,000 and beyond (Mirabilomycota, Parakickxellomycetes, and Sumavosporidiomycetes) based on phylogenies and clustering analyses. This is markedly less than the richness of most extant fungal phyla, whose members number in the thousands based on DNA sequences (Abarenkov et al. 2024). Still, the values compare well with some less speciose fungal phyla, such as Zoopagomycota and Blastocladiomycota, and especially Calcarisporiellomycota, which comprises only two described species. Here, the newly described Calcarisporiellomycota order Terrincolales (50–70 species) is more diverse than the previously described Calcarisporiellales (2 species). Furthermore, many previously described fungal classes are monospecific (e.g., Barbatosporomycetes and Novakomycetes), indicating that very small deep lineages are common in fungi and, thus, many more such lineages are expected to be found. A few putatively phylum- and class-level orphan lineages, with insufficient records for formal description, are indicated in the phylogram (Fig. 1). Collectively, the newly described taxa harbor around 5,000–6,000 species, roughly corresponding to 3.1–3.7% of the 165,000 accepted fungal species and up to 0.20–0.25% of the estimated fungal richness of 2–3 million species (Niskanen et al. 2023).

### ﻿Overview of novel taxa

Out of the 30 major PULs, our analysis revealed that Tartumycota (represented by *Tartumyces setoi* sp. nov.), formerly known as clade BCG2 or freshol1, forms a sister group to all other fungi with strong statistical support (Fig. 1, Suppl. material 1: fig. S1) and warrants a subkingdom of its own (*Tartumyceta* subreg. nov.). Although most records originate from the soil environment, single-cell images of Tartumycota sp. indicate its potentially parasitic associations with green algae in aquatic habitats (Seto et al. 2023). This group likely parasitizes soil surface chlorophytes as well.

The phylum Rozellomycota (syn. Cryptomycota) harbors phylogenetically and functionally highly diverse groups, including extracellular and intracellular parasites (Fig. 1; Quandt et al. 2023). Using a deep sampling of Rozellomycota, we find that the two previously reported long-branching clades, GS01 (described as class Sumavosporidiomycetes, represented by *Sumavosporidium sylvestre* sp. nov.) and GS15 (Gelotisporidiomycetes, represented by *Gelotisporidium boreale* sp. nov.), form deep, well-supported lineages within this phylum. Both classes occur mainly in soil. As with all other members of rozellids, these groups are believed to be parasites.

Aphelids (subkingdom Aphelidiomyceta) comprise the phylum Aphelidiomycota, in which we propose a new class, Pantelleriomycetes (type species, *Pantelleria saittana* sp. nov.). Pantelleriomycetes forms a deep-diverging but well-supported sister clade to the rest of the Aphelidiomycota. Individual records of this group are derived mainly from soil but also from water, sediment, and plant leaves across all continents. There is no unequivocal indication of a putative lifestyle for Pantelleriomycetes species. Still, we hypothesize that the members of this group are parasites, consistent with the behavior observed in all other known aphelids (Karpov et al. 2014).

Chytrids (subkingdom Chytridiomyceta) harbor multiple novel lineages. In Chytridiomycota s. stricto, our analyses reveal the new classes Aquieurochytriomycetes (*Aquieurochytrium lacustre* sp. nov.), Edaphochytriomycetes (*Edaphochytrium valuojaense* sp. nov.), Tibetochytriomycetes (*Tibetochytrium taylorii* sp. nov.), and Tropicochytriomycetes (*Tropicochytrium toronegroense* sp. nov.) and the order Paraspizellomycetales (also known as clade GS14: *Paraspizellomyces parrentiae* sp. nov.) within the class Spizellomycetes. Edaphochytriomycetes has highly divergent subclades and is placed as a sister group of Mesochytriomycetes with poor statistical support. Together with Rhizophlyctidomycetes and Spizellomycetes, Tibetochytriomycetes form a sister group of Rhizophydiomycetes. Tropicochytriomycetes and Aquieurochytriomycetes have an uncertain position within Chytridiomycota. While Aquieurochytriomycetes is common in soil, sediment, and freshwater habitats, Edaphochytriomycetes, Paraspizellomycetales, Tropicochytriomycetes, and Tibetochytriomycetes are found almost exclusively in soil. We also found several novel clades in Neocallimastigomycota, the most prominent of which we name Aquamastigomycetes (*Aquamastix sanduskyensis* sp. nov.), Cantoromastigomycetes (*Cantoromastix holarctica* sp. nov.), Dobrisimastigomycetes (*Dobrisimastix vlkii* sp. nov.), Palomastigomycetes (*Palomastix lacustris* sp. nov.), and Sedimentomastigomycetes (*Sedimentomastix tueriensis* sp. nov.). Unlike other soil-inhabiting classes, Palomastigomycetes, Aquamastigomycetes, Sedimentomastigomycetes, and a yet-unnamed Neocallimastigomycetes clade GS58 are found mainly in sediments. As the latter three groups are successive sisters to Neocallimastigales—known as anaerobic gut symbionts of herbivorous mammals and turtles (Pratt et al. 2023)—ancestors of this order may have acquired animal symbiosis in an already anaerobic state. Our extended analyses also reveal that the novel class Algovoracomycetes (known as clade GS13 or NC-ChyL1) is a member of Monoblepharomycota rather than Chytridiomycota (Tedersoo et al. 2017; Seto et al. 2023). The Algovoracomycetes appear to be parasites of green algae (Ding et al. 2018; Seto et al. 2023). Here, we propose recombining the species *Algovorax scenedesmi* (basionym *Phlyctidium scenedesmi*) and suggest an epitype based on available material (see below). Within Algovoracomycetes, we also describe another, more common order, namely Solivoragomycetales, based on *Solivorax pantropicus* sp. nov. Both Chytridiomycota and Neocallimastigomycota harbor several additional class-level clades (Fig. 1, Suppl. material 1: fig. S1).

In the zoosporic phylum Olpidiomycota (subreg. Olpidiomyceta), we describe the three earliest diverging lineages at the class level: Bryolpidiomycetes (represented by *Bryolpidium mundanum* sp. nov.), Chthonolpidiomycetes (*Chthonolpidium enigmatum* sp. nov.), and Savannolpidiomycetes (*Savannolpidium raadiense* sp. nov.). Bryolpidiomycetes is recorded in soil and moss samples, whereas Chthonolpidiomycetes is found in soil, and Savannolpidiomycetes occurs in soil and sediments. Species of Olpidiomycota are obligate intracellular pathogens of plants (Lay et al. 2018). Therefore, we hypothesize that the members of Bryolpidiomycetes, Chthonolpidiomycetes, and Savannolpidiomycetes may be pathogens of bryophytes and algae.

The zoosporic zygomycetes from subkingdom Zoopagomyceta accommodate six additional phyla, viz., Aldinomycota (represented by *Aldinomyces tarquinii* sp. nov.), Borikeniomycota (*Borikenia urbinana* sp. nov.), Mirabilomycota (*Mirabilomyces abrukanus* sp. nov.), Nematovomycota (*Nematovomyces vermicola* comb. nov. and *N. soinasteënsis* sp. nov.), Viljandiomycota (*Viljandia globalis* sp. nov.), and Waitukubulimycota (*Waitukubulimyces cliftonii* sp. nov.), as well as the new class Parakickxellomycetes (known as clade GS15; *Parakickxella borikenica* sp. nov.) within the Kickxellomycota. While these new taxa are statistically well supported, their relationships to each other change depending on analysis parameters and the inclusion of additional taxa. While Aldinomycota, Viljandiomycota, and Basidiobolomycota seem to have a low rate of rRNA gene evolution as deduced from branch lengths, other phyla of *Zoopagomyceta* display relatively rapid rRNA gene evolution. All these novel groups have been almost exclusively recovered from soil samples, and they likely represent either saprotrophs or animal parasites, i.e., lifestyles common to the previously known phyla of the subkingdom, viz., Basidiobolomycota, Kickxellomycota, Entomophthoromycota, and Zoopagomycota. While the other groups are newly described, Nematovomycota accommodates “*Olpidium*” vermicola, for which we propose a new name, *Nematovomyces vermicola* (see below), because all other sequenced species of *Olpidium* are placed in the subkingdom Olpidiomyceta. Several species of Nematovomycota have been identified as parasites of nematodes, rotifers, or their eggs (Glockling 1998; Seto et al. 2023).

In the group of zygomycetes, Mucoromyceta, our analysis reveals the new phylum Curlevskiomycota (represented by *Curlevskia holarctica* sp. nov.), which forms a well-supported sister group to the phylum Glomeromycota. Since these species have been found almost exclusively in soil samples rather than roots, this group likely represents saprotrophs rather than arbuscular mycorrhizal symbionts, an otherwise exclusive strategy in Glomeromycota. We also propose the new order Terrincolales (*Terrincola waldropii* sp. nov.), a sister group to Calcarisporiellales in Calcarisporiellomycota. Within Mortierellomycota, we describe two new classes, Maerjamycetes (*Maerjamyces jumpponenii* sp. nov.) and Ruderaliomycetes (*Ruderalia cosmopolita* sp. nov.), as well as the new order Mycosocceriales (*Mycosocceria estonica* sp. nov.) within Mortierellomycetes. All four of these groups commonly occur in disturbed urban and cropland soils, suggesting a somewhat copiotrophic lifestyle characteristic of the closely related groups Mucorales and Calcarisporiellales. However, the lack of success in culturing these relatively common groups suggests a potential biotrophic lifestyle.

### ﻿Descriptions of new species and higher-ranking taxa

Here, we provide formal descriptions from species through genera to phyla and propose two species-level combinations. In diagnoses of genera and higher-ranking taxa, diagnostic nucleotides that specifically differ from the closest related taxa are underlined. The indicated nucleotide positions are numbered relative to the legitype of the type species (ITS region and LSU) and *Saccharomyces cerevisiae* (SSU, 5.8S, and LSU).

#### Fungi

Taxon classificationFungiFungiFungi

R.T. Moore Botanica Marina 23(6): 371 (1980)

789DCE7E-9D36-5502-A642-775FB1F8B674

90155

##### Type phylum.

None.

##### Description.

As in Moore (1980).

##### Notes.

Currently harbors the subkingdoms Aphelidiomyceta, Blastocladiomyceta, Chytridiomyceta, Dikarya, Mucoromyceta, Olpidiomyceta, Rozellomyceta, Zoopagomyceta, and Tartumyceta (subreg. nov).

#### Aphelidiomyceta

Taxon classificationFungiFungiAphelidiomyceta

Tedersoo, Sanchez-Ramirez, Kõljalg, Bahram, M. Döring, Schigel, T.W. May, M. Ryberg & Abarenkov, Fungal Diversity 90: 147 (2018)

59FF4FA3-C591-5D9B-A37C-B247CAD6B746

553989

##### Type class.

Aphelidiomycota Tedersoo, Sanchez-Ramirez, Kõljalg, Bahram, M. Döring, Schigel, T.W. May, M. Ryberg & Abarenkov.

##### Description.

As in Tedersoo et al. (2018).

##### Notes.

Currently harbors Aphelidiomycota.

#### Aphelidiomycota

Taxon classificationFungiAphelidiomycetaAphelidiomycota

Tedersoo, Sanchez-Ramirez, Kõljalg, Bahram, M. Döring, Schigel, T.W. May, M. Ryberg & Abarenkov, Fungal Diversity 90: 147 (2018)

66C30CDD-AAE1-5209-8DF9-5B0129A7512F

553990

##### Type class.

Aphelidiomycetes Tedersoo, Sanchez-Ramirez, Kõljalg, Bahram, M. Döring, Schigel, T.W. May, M. Ryberg & Abarenkov.

##### Description.

As in Tedersoo et al. (2018).

##### Notes.

Currently harbors Aphelidiomycetes and Pantelleriomycetes (class. nov.).

#### Pantelleriomycetes

Taxon classificationFungiAphelidiomycetaAphelidiomycota

Tedersoo class. nov.

199821CC-2B65-58E3-A970-E6760E830C88

858887

##### Type order.

Pantelleriales Tedersoo.

##### Diagnosis.

Distinguishable from other Aphelidiomycota based on diagnostic nucleotide signature in LSU 5’ end (positions 42–51 in type species and *S. cerevisiae* tatcattaag; no mismatch allowed). Forms a monophyletic, least inclusive clade in Aphelidiomycota, covering sequences EUK1203048, EUK1205018, EUK1200019, EUK1137930, EUK1120815, EUK1103262, GU568135, EUK1200059, EUK1138870, EUK1138872, EUK1120814, EUK1102231, and EUK1201826 (Fig. 1).

##### Notes.

Recognized based on eDNA sequences only. Encoded as clade GS55 in EUKARYOME v1.9. Currently harbors Pantelleriales (ord. nov.) and potentially order-level groups represented by sequences EUK1203048 (forest soil in Italy), EUK1205018 (forest soil in Italy), GU568135 (experimental soil in China), EUK1200059 (forest soil in Estonia), EUK1102231 (lake water in Sweden), EUK1120815 (cropland soil in Estonia), EUK1201826 (forest soil in Italy), EUK1138870 (forest soil in New Zealand), EUK1138872 (forest soil in New Zealand), EUK1120814 (urban soil in Estonia), EUK1671420 (forest soil in NA, USA), EUK1103262 (lake sediment in Sweden), and EUK1700281 (desert soil in Oman). Comprises potentially 170–200 species. Detected in soil (98.9% out of 633 records), freshwater (0.5%), sediments (0.3%), and plant leaves (0.3%) in high arctic to wet tropical biomes across all continents, including Antarctica.

#### Pantelleriales

Taxon classificationFungiAphelidiomycetaPantelleriomycetes

Tedersoo ord. nov.

5920EF54-D087-557F-BA2E-65AA00F2FDEA

858888

##### Type family.

Pantelleriaceae Tedersoo.

##### Diagnosis.

Distinguishable from other fungi based on diagnostic nucleotide signature in SSU V7 (positions 1389–1408 in *S. cerevisiae* ctatcgacgtwtagtcgatg; no mismatch allowed). Forms a monophyletic, least inclusive clade in Pantelleriomycetes, covering sequences EUK1200019, EUK1137930, EUK1120813, and EUK1700280 (Fig. 1).

##### Notes.

Recognized based on eDNA sequences only. Currently includes Pantelleriaceae (fam. nov.) and potential family-level groups represented by sequences EUK1700280 (forest soil in MI, USA), EUK1217356 (lake sediment in Brazil), EUK1138063 (moss sample in Estonia), EUK1138061 (wasteland soil in Estonia), EUK0348101 (forest soil in the Canary Islands), EUK0348102 (desert soil in Oman), EUK0348062 (desert soil in Qatar), EUK0348068 (grassland soil in Bangladesh), EUK0348055 (woodland soil in Ghana), EUK0348090 (shrubland soil in Argentina), and EUK1120813 (lake sediment in Ethiopia).

#### Pantelleriaceae

Taxon classificationFungiPantelleriomycetesPantelleriales

Tedersoo fam. nov.

E1A8C972-0942-56BF-AF1B-0DCA2BFA8931

858892

##### Type genus.

*Pantelleria* Tedersoo.

##### Diagnosis.

Distinguishable from other fungi based on diagnostic nucleotide signature in SSU V9 (positions 1671–1684 in *S. cerevisiae* gaamctcggatcgtt; one mismatch allowed), and 5.8S (positions 119–133 in type species and 120–134 in *S. cerevisiae* ataggtattcctrtg; one mismatch allowed). Forms a monophyletic, least inclusive clade in Pantelleriales, covering sequences EUK1200019 and EUK1137930 (Fig. 1).

##### Notes.

Recognized based on eDNA sequences only. Currently includes *Pantelleria* (gen. nov.).

#### Pantelleria

Taxon classificationFungiPantellerialesPantelleriaceae

Tedersoo gen. nov.

0C93F2C0-9614-5E57-9A4C-29093B795F7D

858894

##### Type species.

*Pantelleria saittana* Tedersoo.

##### Diagnosis.

**Figure 2.**
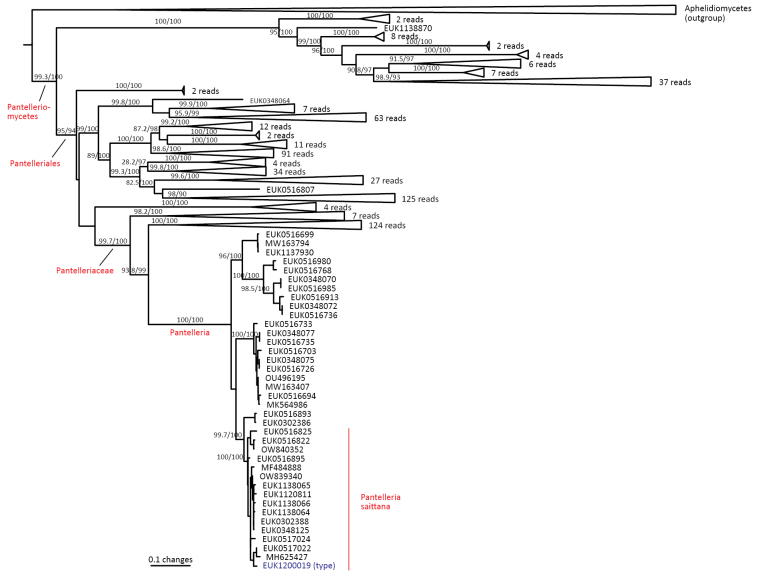
Maximum Likelihood SSU-ITS-LSU phylogram indicating the position of *Pantelleria saittana* within Pantelleriomycetes, with ultra-rapid bootstrap values indicated (for higher-level classifications mainly). Other genus-level groups are collapsed. Aphelidiomycota spp. were used as an outgroup.

Distinguishable from other fungi based on diagnostic nucleotide signature in SSU V9 (positions 1671–1684 gaamctcggatcgtt in *S. cerevisiae*; one mismatch allowed), ITS2 (positions 99–113 tcatttacttttaag in type species; one mismatch allowed), and 5.8S (positions 119–133 in type species and 120–134 in *S. cerevisiae* ataggtattcctrtg; one mismatch allowed). Forms a monophyletic, least inclusive clade in Pantelleriaceae, covering sequences EUK1200019 and EUK1137930 (Figs 1, 2).

##### Notes.

Recognized based on eDNA sequences only. Contains 6–7 potential species represented by sequences MW163794 (cropland soil in Italy), OU496195 (unspecified soil in China), EUK0348072 (cropland soil in Benin), EUK0348070 (woodland soil in Turkey), EUK1137930 (urban soil in Estonia), and EUK0516893 (park soil in Estonia).

#### Pantelleria saittana

Taxon classificationFungiPantellerialesPantelleriaceae

Tedersoo sp. nov.

3D7B89BF-1BA5-5054-AC0D-06CEF12A5232

858898

##### Diagnosis.

**Figure 3.**
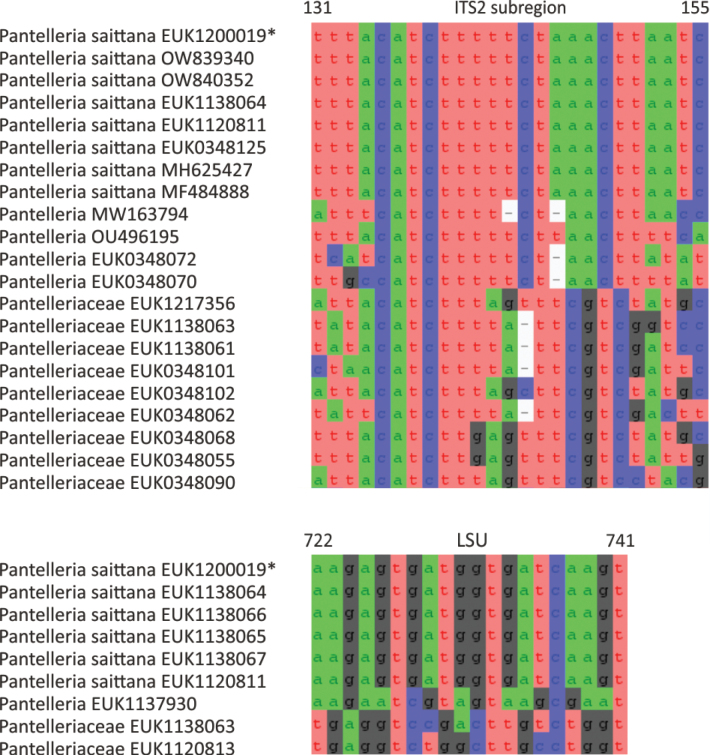
Diagnostic nucleotide sequences of *Pantelleria saittana* relative to the closest related species in ITS2 and LSU. Numbers indicate positions in the legitype (marked with an asterisk).

Separation from other species of *Pantelleria* based on ITS2 (positions 131–155 tttacatctttttctaaacttaatc; one mismatch allowed) and LSU D2 (positions 722–741 aagagtgatggtgatcaagt; one mismatch allowed) as indicated in Fig. 3. Intraspecific variation up to 3.8% in ITS2 and up to 1.5% in LSU. Interspecific distance at least 10.1% in ITS2.

##### Type.

Vouchered soil sample TUE000518 (**holotype**); eDNA sequence EUK1200019 = OZ253786 (**legitype**); eDNA sample TUE100518 (**nucleotype**); GSMc plot G3487, *Quercus ilex* forest soil in Ponta Spadillo, Pantelleria, Italy, 36.8185°N, 12.0149°E.

##### Description.

Other sequences: OW839340 and OW840352 (unspecified soil in Tianshan Mountains, Uyghur Autonomous Region, China) ; EUK1138064 (GSMc plot G4627, mixed forest soil in Tudusoo, Estonia, 59.11368°N, 26.75944°E); EUK1120811 (GSMc plot S281, *Quercus robur* alley soil in Tartu, Estonia, 58.379°N, 26.706°E); EUK0348125 (urban park soil in Niort, France, 46.325, –0.4672°E); MH625427 (microcosm soil in New Zealand); and MF484888 (unspecified soil in Great Britain).

##### Etymology.

*>Pantelleria* (Maltese) refers to the type locality, and *Saitta* (Sicilian) refers to *Alessandro Saitta*, who collected material from the type locality.

##### Notes.

Found in soil samples and occasionally in oceanic sediments in temperate and subtropical regions worldwide (n = 18 records). The 86 GlobalFungi records confirm global soil distribution.

#### Chytridiomyceta

Taxon classificationFungiFungiFungi

Tedersoo, Sanchez-Ramirez, Kõljalg, Bahram, M. Döring, Schigel, T.W. May, M. Ryberg & Abarenkov, Fungal Diversity 90: 148 (2018)

65AF7815-3662-523F-8BB3-9E6669C93901

553996

##### Type class.

Chytridiomycota Doweld.

##### Description.

As in Tedersoo et al. (2018).

##### Notes.

Currently harbors the phyla Chytridiomycota, Monoblepharomycota, and Neocallimastigomycota.

#### Chytridiomycota

Taxon classificationFungiFungiChytridiomyceta

Doweld, Prosyllabus Tracheophytorum, Tentamen systematis plantarum vascularium (Tracheophyta): LXXVII (2001)

BA5B3EFB-0C63-50A9-99F1-6BEEFECAFD47

90736

##### Type class.

Chytridiomycetes Caval.-Sm.

##### Description.

As in Doweld (2001).

##### Notes.

Currently harbors the classes Caulochytriomycetes, Chytridiomycetes, Cladochytriomycetes, Lobulomycetes, Mesochytriomycetes, Polychytriomycetes, Rhizophydiomycetes, Rhizophlyctidomycetes, Spizellomycetes, Synchytriomycetes, Aquieurochytriomycetes (class. nov), Edaphochytriomycetes (class. nov.), Tibetochytriomycetes (class. nov.), and Tropicochytriomycetes (class. nov.), and potentially class-level groups represented by sequences EUK1102715 (forest soil in Puerto Rico), EUK1107652 (peatland soil in Sweden), and EUK1104403 (forest soil in Sweden).

#### Spizellomycetes

Taxon classificationFungiChytridiomycetaChytridiomyceta

Tedersoo, Sanchez-Ramirez, Kõljalg, Bahram, M. Döring, Schigel, T.W. May, M. Ryberg & Abarenkov, Fungal Diversity 90: 149 (2018)

023AAF1B-B47D-52E7-BB9E-82FD194D8C37

554003

##### Type order.

Spizellomycetales D.J.S. Barr.

##### Description.

As in Tedersoo et al. (2018).

##### Notes.

Currently harbors Spizellomycetales and Paraspizellomycetales (ord. nov.).

#### Paraspizellomycetales

Taxon classificationFungiChytridiomycetaChytridiomyceta

Tedersoo ord. nov.

A5C16A3D-11ED-577C-A582-5644C11E5AAD

858901

##### Type family.

Paraspizellomycetaceae Tedersoo.

##### Diagnosis.

Distinguishable from other fungi based on a diagnostic nucleotide signature in LSU D1 (positions 128–142 in type species and 123–137 in *S. cerevisiae* cggttcgccggtgcg or gggttcttacctatg or gggttccacctatgc; one mismatch allowed). Forms a monophyletic, least inclusive clade in Spizellomycetes, covering sequences EUK1138322, EUK1152022, EUK1187448, EUK1202178, EUK1187441, EUK1187447, UDB014658, EUK1100628, EUK1123745, and EUK1139262 (Fig. 1).

##### Notes.

Recognized based on eDNA sequences only. Encoded as clade GS14 in EUKARYOME v1.9. Currently includes Paraspizellomycetaceae (fam. nov.) and one or more potentially family-level groups represented by sequences EUK1138322, EUK1152022 (both forest soil in New Zealand), EUK1187448 (forest soil in Chile), and EUK1202178 (tundra soil in Norway). Comprises potentially around 50–70 species. Detected in soil (94.6% out of the 159 records), sediments (4.2%), and freshwater (1.2%) in tundra to tropical biomes across all continents except Antarctica.

#### Paraspizellomycetaceae

Taxon classificationFungiChytridiomycetaParaspizellomycetales

Tedersoo fam. nov.

2C2903E2-4565-5248-8C52-A534C8F54406

858903

##### Type genus.

*Paraspizellomyces* Tedersoo.

##### Diagnosis.

Distinguishable from other fungi based on diagnostic nucleotide signatures in LSU D2 (positions 578–592 in type species and 562–576 in *S. cerevisiae* aaggtcatgcttt; one mismatch allowed) and ITS2 (positions 134–148 in type species aatggttcccaagtg; two mismatches allowed). Forms a monophyletic, least inclusive clade in Paraspizellomycetales, covering sequences EUK1187441, EUK1187447, UDB014658, EUK1100628, EUK1123745, and EUK1139262 (Fig. 1).

##### Notes.

Recognized based on eDNA sequences only. Includes *Paraspizellomyces* (gen. nov.) and another potentially genus-level group represented by sequences EUK1123745 (forest soil in Estonia), and EUK1139262 (forest soil in New Zealand).

#### Paraspizellomyces

Taxon classificationFungiParaspizellomycetalesParaspizellomycetaceae

Tedersoo gen. nov.

75DAB7E4-C1B7-5619-BFF3-8377DCF725F0

858904

##### Type species.

*Paraspizellomyces parrentiae* Tedersoo.

##### Diagnosis.

**Figure 4.**
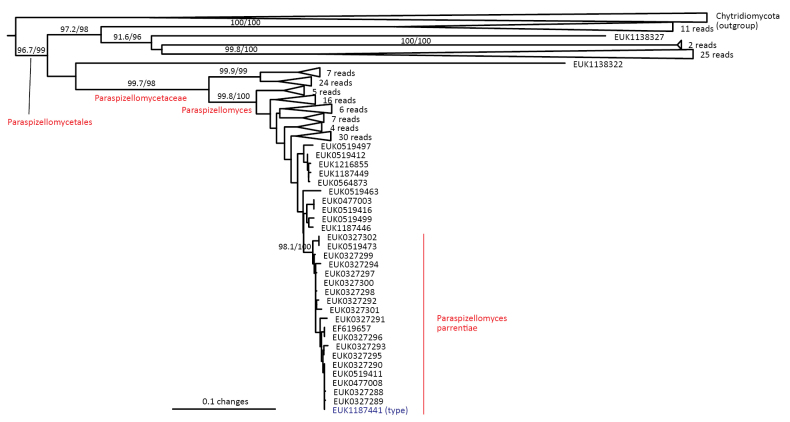
Maximum Likelihood SSU-ITS-LSU phylogram indicating the position of *Paraspizellomyces parrentiae* within Paraspizellomycetales, with ultra-rapid bootstrap values indicated (for higher-level classifications mainly). Other genus-level groups are collapsed. Chytridiomycota spp. were used as an outgroup.

Distinguishable from other fungi based on diagnostic nucleotide signatures in LSU D1 (positions 228–237 in type species and 227–236 in *S. cerevisiae* taacgaccca; one mismatch allowed) and SSU V9 (positions 1695–1709 in *S. cerevisiae* aatttcggttgctgg or agtttcggccgctgg; one mismatch allowed). Forms a monophyletic, least inclusive clade in Paraspizellomycetaceae, covering sequences EUK1187441, EUK1187447, UDB014658, and EUK1100628 (Figs 1, 4).

##### Notes.

Recognized based on eDNA sequences only. Comprises potentially 20–30 species represented by sequences EUK1187447 (forest soil in Puerto Rico), UDB014658 (forest soil in Madagascar), EUK1100628 (forest soil in Puerto Rico), and GQ921827 (forest soil in Australia).

#### Paraspizellomyces parrentiae

Taxon classificationFungiParaspizellomycetalesParaspizellomycetaceae

Tedersoo sp. nov.

29EC75DD-EBF8-5828-A95E-588A79B3F4CD

858905

##### Diagnosis.

**Figure 5.**
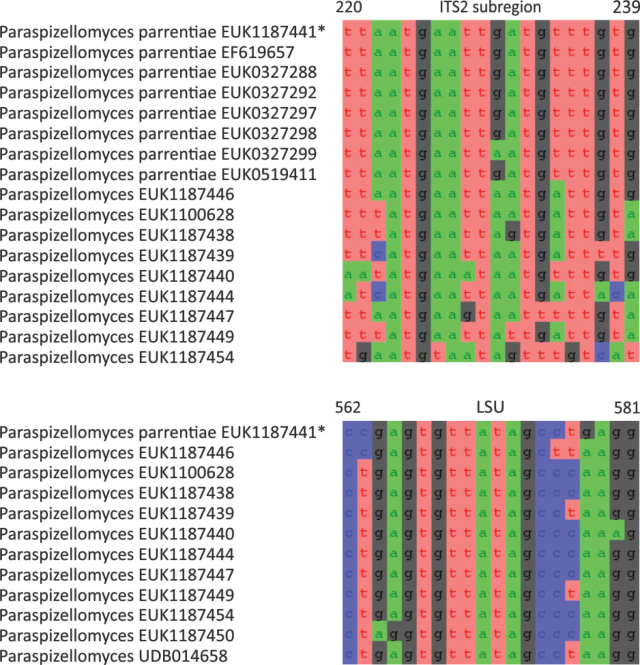
Diagnostic nucleotide sequences of *Paraspizellomyces parrentiae* relative to the closest related species in ITS2 and LSU. Numbers indicate positions in the legitype (marked with an asterisk).

Separation from other species of *Paraspizellomyces* based on ITS2 (positions 220–239 tttatgaattartgattgta; no mismatch allowed) and LSU (positions 562–581 ccgagtgttatagcctgagg; no mismatch allowed) as indicated in Fig. 5. Intraspecific variation up to 3.7% in ITS2. Interspecific distance at least 3.7% in ITS2; i.e., there is no clear barcode gap in ITS2. In ITS1, the maximum intraspecific difference 2.4%, and the minimum interspecific distance 3.3%.

##### Type.

Vouchered soil sample TUE002024 (**holotype**); eDNA sequence EUK1187441 = OZ253787 (**legitype**); eDNA sample TUE102024 (**nucleotype**) GSMc plot G5047, tropical rainforest forest soil in Morne Louis, Guadeloupe, 16.1856, –61.7450.

##### Description.

Other sequences: EF619657 (soil in *Pinus taeda* plantation, NC, USA); EUK0327288 (GSMc plot G6004, tropical rainforest soil in Cascada Julieta in Panama, 9.2274, –79.4312); EUK0327292 (GSMc plot G4982, subtropical rainforest soil in Weiloi, Meghalaya, India, 25.3570°N, 91.6060°E); EUK0327297 (GSMc plot S372, subtropical forest soil in Menglun, Yunnan, China, 21.572°N, 101.57°E); EUK0327298 (GSMc plot S013, tropical woodland soil in Isalo, Madagascar, –22.5339, 45.3703); EUK0327299 (GSMc plot S765, tropical rainforest soil in Mbomole, Tanzania, –5.0946, 38.6292); and EUK0519411 (GSMc plot S1190, tropical rainforest soil in La Palma, Costa Rica, 10.5046, –84.6949).

##### Etymology.

*Para* (Greek) and *Spizellomyces* (Latin) refer to phylogenetic relatedness to Spizellomycetales, and *Parrent* (English) refers to Jeri Lynn Parrent, who was the first to collect material from this species (EF619657; Parrent and Vilgalys 2007).

##### Notes.

Found in 18 soil samples in tropical and warm temperate forest habitats in North and South America, Africa, and Asia. The 33 additional GlobalFungi records confirm these findings but add that plant tissues may be an additional habitat (12.1% of records).

#### Aquieurochytriomycetes

Taxon classificationFungiChytridiomycetaChytridiomyceta

Tedersoo & Esmaeilzadeh-Salestani class. nov.

419B76C4-64CC-5F87-9FAE-A1FFB4AAD13E

858909

##### Type order.

Aquieurochytriales Tedersoo & Esmaeilzadeh-Salestani.

##### Diagnosis.

Distinguishable from other fungi based on a diagnostic nucleotide signature in LSU D1 (positions 171–185 in type species and *S. cerevisiae* ggcaagccgggcaaa OR ggctgctcggacaaa; two mismatches allowed). Forms a monophyletic, least inclusive clade in Chytridiomycota, covering sequences EUK1107407, AB971081, EUK1100022, EUK1102113, EUK1123700, and EUK1102276 (Fig. 1).

##### Notes.

Recognized based on eDNA sequences only. Encoded as clade GS59 in EUKARYOME v1.9. Currently harbors Aquieurochytriales (ord. nov.) and potentially order-level groups represented by sequences EUK1107407 (peatland soil in Sweden), EUK0130469 (woodland soil in Australia), EUK0519470 (cropland soil in Estonia), and EUK0569228 (freshwater sediment in Estonia). Comprises potentially 50–60 species. Detected in water (53.8% out of the 80 records), sediments (32.5%), and soil (13.7%) in tundra to tropical biomes in all continents except Antarctica.

#### Aquieurochytriales

Taxon classificationFungiChytridiomycetaAquieurochytriomycetes

Tedersoo & Esmaeilzadeh-Salestani ord. nov.

5D92AAD1-D97C-5062-B56F-2830AB425770

858910

##### Type family.

Aquieurochytriaceae Tedersoo & Esmaeilzadeh-Salestani.

##### Diagnosis.

Distinguishable from other fungi based on diagnostic nucleotide signatures in LSU D1 (positions 171–185 in type species and *S. cerevisiae* ggcaagccgggcaaa; one mismatch allowed), SSU V4 (positions 871–885 in *S. cerevisiae* atactttcattagtc; one mismatch allowed), and ITS2 (positions 173–195 in type species taatgctgggcgtcagcctgctt OR taatgacgggcgtcagcctgctt; three mismatches allowed). Forms a monophyletic, least inclusive clade in Aquieurochytriomycetes, covering sequences AB971081, EUK1100022, EUK1102113, EUK1123700, and EUK1102276 (Fig. 1).

##### Notes.

Recognized based on eDNA sequences only. Currently includes Aquieurochytriaceae (fam. nov.).

#### Aquieurochytriaceae

Taxon classificationFungiAquieurochytriomycetesAquieurochytriales

Tedersoo & Esmaeilzadeh-Salestani fam. nov.

A37740D8-E196-5B99-8E66-02B8C1C7E3EA

858911

##### Type genus.

*Aquieurochytrium* Tedersoo & Esmaeilzadeh-Salestani.

##### Diagnosis.

Distinguishable from other fungi based on diagnostic nucleotide signatures in LSU D1 (positions 171–185 in type species and *S. cerevisiae* ggcaagccgggcaaa; one mismatch allowed), SSU V4 (positions 871–885 in *S. cerevisiae* atactttcattagtc; one mismatch allowed), and ITS2 (positions 173–195 in type species taatgctgggcgtcagcctgctt OR taatgacgggcgtcagcctgctt; three mismatches allowed). Forms a monophyletic, least inclusive clade in Aquieurochytriales, covering sequences AB971081, EUK1100022, EUK1102113, EUK1123700, and EUK1102276 (Fig. 1).

##### Notes.

Recognized based on eDNA sequences only. Includes *Aquieurochytrium* (gen. nov.) and other potentially genus-level groups represented by sequences AB971081 (water in Japan), EUK1123700 (freshwater sediment in New Zealand), and EUK1100022 (permafrost in Canada).

#### Aquieurochytrium

Taxon classificationFungiAquieurochytrialesAquieurochytriaceae

Tedersoo & Esmaeilzadeh-Salestani gen. nov.

CD469F26-8421-56BD-9CF0-5B38F3BBABCC

858912

##### Type species.

*Aquieurochytrium lacustre* Tedersoo & Esmaeilzadeh-Salestani.

##### Diagnosis.

**Figure 6.**
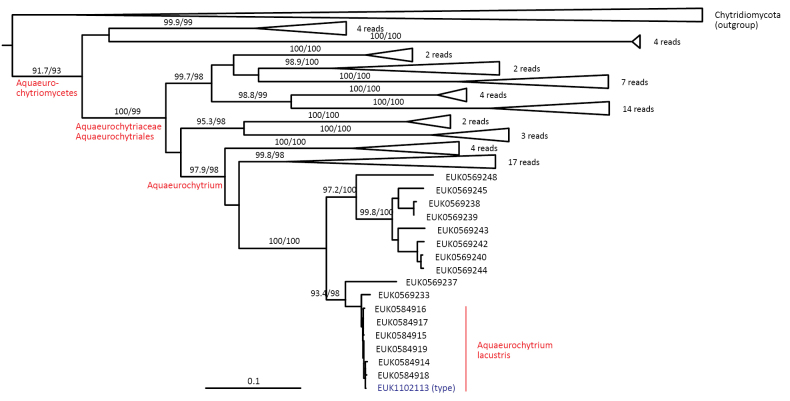
Maximum Likelihood SSU-ITS-LSU phylogram indicating the position of *Aquieurochytrium lacustre* within Aquieurochytriomycetes, with ultra-rapid bootstrap values indicated (for higher-level classifications mainly). Other genus-level groups are collapsed. Chytridiomycota spp. were used as an outgroup.

Distinguishable from other fungi based on diagnostic nucleotide signatures in ITS2 (positions 160–168 ccgcgacga; one mismatch allowed) and LSU D2 (positions 618–637 in type species and 591–610 in *S. cerevisiae* tcgcagcgcaccgtaaggcg). Forms a monophyletic, least inclusive clade in Aquieurochytriaceae, covering sequences EUK1102113 and EUK1102276 (Figs 1, 6).

##### Notes.

Recognized based on eDNA sequences only. Comprises potentially 25–30 species represented by sequences EUK1102276 (lake water in Sweden), EUK0569233 (lake water in Estonia), and EUK0569237 (lake water in Benin).

#### Aquieurochytrium lacustre

Taxon classificationFungiAquieurochytrialesAquieurochytriaceae

Tedersoo & Esmaeilzadeh-Salestani sp. nov.

602728F4-A522-57E7-8C93-3EB92F79E768

858913

##### Diagnosis.

**Figure 7.**
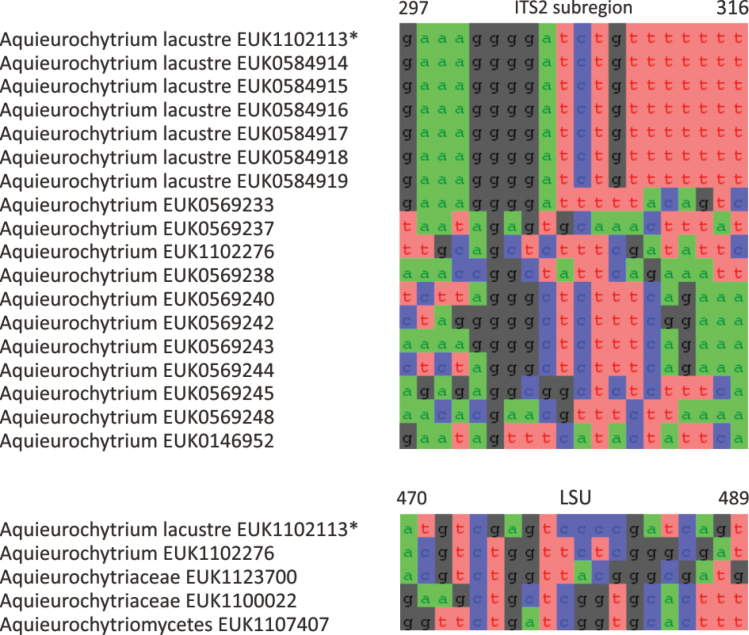
Diagnostic nucleotide sequences of *Aquieurochytrium lacustre* relative to the closest related species in ITS2 and LSU. Numbers indicate positions in the legitype (marked with an asterisk).

Separation from other species of *Aquieurochytrium* based on the ITS2 (positions 297–316 gaaaggggatctgttttttt; one mismatch allowed) and LSU D2 (positions 470–489 in type species and 450–469 in *S. cerevisiae* atgtcgagtccccgatcagt; no mismatch allowed) as indicated in Fig. 7. Intraspecific variation up to 1.1% in ITS2. Interspecific distance at least 3.4% in ITS2.

##### Type.

Vouchered aquatic eDNA sample TUE128819 (**holotype**); eDNA sequence EUK1102113 = OZ253788 (**legitype**); freshwater in Lake Skogaryd, Sweden, 58.37°N, 12.16°E.

##### Description.

Other sequences: EUK0584914 (FunAqua sample W0790w; lake water in Beukenlaan, Netherlands, 52.000°N, 4.487°E); EUK0584915 (FunAqua sample W0038w; water in Lake Luke Vanajärv, Estonia, 58.2438°N, 26.5751°E); EUK0584916 (FunAqua sample W0458w; water in Lake Stübnitzsee, Germany, 53.1071°N, 13.1891°E); EUK0584917 (FunAqua sample W0624w; water in Lake Vejlsø, Denmark, 56.1514°N, 9.5618°E); EUK0584918 (FunAqua sample W0454w; water in Lake Kleiner Wentowsee, Germany, 53.4494°N, 13.1052°E); and EUK0584919 (FunAqua sample W0364s; sediment in Lake Ototoa, New Zealand, –36.5302, 174.2324).

##### Etymology.

*Aqua* (Latin) and *Europa* (Greek) refer to the habitat in European waters; and *lacuster* (Latin) specifies the lake habitat.

##### Notes.

Found in six temperate and boreal freshwater lakes in Central and Northern Europe, with one record from New Zealand (sequences differ by one nucleotide from closest European records; sequenced in an independent library). The eight additional GlobalFungi records originate from lake water in Scandinavia.

#### Edaphochytriomycetes

Taxon classificationFungiChytridiomycetaChytridiomyceta

Tedersoo class. nov.

803D1E66-2962-51A0-AA02-6341AEFC1E66

858914

##### Type order.

Edaphochytriales Tedersoo.

##### Diagnosis.

Distinguishable from other fungi based on a diagnostic nucleotide signature in 5.8S (positions 45–64 in type species and *S. cerevisiae* catagtgaaatgtgataact or catggtgaaatgtgacaatt; one mismatch allowed). Forms a monophyletic, least inclusive clade in Chytridiomycota, covering sequences EUK1104126, EUK1107474, EUK1671450, EUK1671451, EUK1008462, EUK1200051, EUK1101631, EUK1101779, EUK1200763, and EUK1123748 (Fig. 1).

##### Notes.

Recognized based on eDNA sequences only. Encoded as clade GS42 in EUKARYOME v1.9. Currently harbors Edaphochytriales (ord. nov.) and potentially an order-level group represented by sequences EUK1104126 (lake water in Sweden) and EUK1107474 (peatland soil in Sweden). Comprises potentially 40–45 species. Detected in soil (94.4% out of the 89 records), sediments (2.2%), glacial ice (2.2%), and freshwater (1.1%) in tundra to wet tropical biomes across all continents except Antarctica.

#### Edaphochytriales

Taxon classificationFungiChytridiomycetaChytridiomyceta

Tedersoo ord. nov.

31016709-247B-5E02-9EDC-2234BA66CA96

858915

##### Type family.

Edaphochytriaceae Tedersoo.

##### Diagnosis.

Distinguishable from other fungi based on a diagnostic nucleotide signature in 5.8S (positions 45–64 catagtgaaatgtgataact in type species and *S. cerevisiae*; no mismatch allowed). Forms a monophyletic, least inclusive clade in Edaphochytriomycetes, covering sequences EUK1671450, EUK1671451, EUK1008462, EUK1200051, EUK1101631, EUK1101779, EUK1123746, EUK1200763, and EUK1123748 (Fig. 1).

##### Notes.

Recognized based on eDNA sequences only. Currently includes Edaphochytriaceae (fam. nov.) and other potentially family-level groups represented by sequences EUK1671450 (forest soil in Guadeloupe), EUK1101631 (permafrost in Canada), EUK1101779 (cropland soil in Great Britain), and EUK1671451 (shrubland soil in Morocco).

#### Edaphochytriaceae

Taxon classificationFungiChytridiomycetaEdaphochytriales

Tedersoo fam. nov.

FF8CD3CA-C7EB-570B-B183-0EEB07F80A1A

858916

##### Type genus.

*Edaphochytrium* Tedersoo.

##### Diagnosis.

Distinguishable from other fungi based on a diagnostic nucleotide signature in the LSU 5’ end (positions 15–24 in the type species and *S. cerevisiae* tagtggacta or tgatggacta; one mismatch allowed). Forms a monophyletic, least inclusive clade in Edaphochytriales, covering sequences EUK1008462, EUK1200051, EUK1123749, EUK1630709, EUK1123746, EUK1200763, and EUK1123748 (Fig. 1).

##### Notes.

Recognized based on eDNA sequences only. Includes *Edaphochytrium* (gen. nov.) and other potentially genus-level groups represented by sequences EUK1123749 and EUK1008462.

#### Edaphochytrium

Taxon classificationFungiChytridiomycetaEdaphochytriaceae

Tedersoo gen. nov.

743523EE-A3FD-58E7-9B13-8FA3F9184DF6

858918

##### Type species.

*Edaphochytrium valuojaense* Tedersoo.

##### Diagnosis.

**Figure 8.**
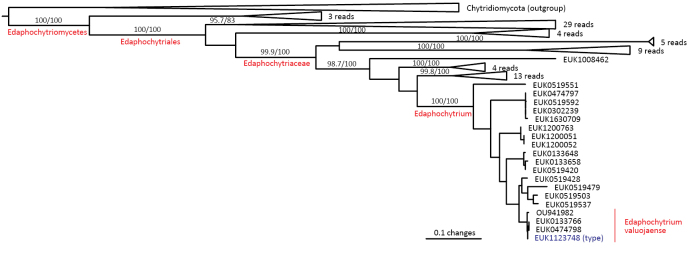
Maximum Likelihood SSU-ITS-LSU phylogram indicating the position of *Edaphochytrium valuojaense* within Edaphochytriomycetes, with ultra-rapid bootstrap values indicated (for higher-level classifications only). Other genus-level groups are collapsed. Chytridiomycota spp. were used as an outgroup.

Distinguishable from other fungi based on a diagnostic nucleotide signature in 5.8S (positions 114–133 in type species and *S. cerevisiae* cagtctcttaaggagataat; one mismatch allowed). Forms a monophyletic, least inclusive clade in Edaphochytriaceae, covering sequences EUK1200051, EUK1630709, EUK1200763, and EUK1123748 (Figs 1, 8).

##### Notes.

Recognized based on eDNA sequences only. Comprises potentially 4–5 species represented by sequences EUK1200051 (forest soil in Estonia), EUK1630709 (forest soil in Estonia), and EUK0133658 (plantation soil in the Canary Islands).

#### Edaphochytrium valuojaense

Taxon classificationFungiChytridiomycetaEdaphochytriaceae

Tedersoo sp. nov.

3CBD05F1-1A47-576B-918D-75F77FA7B862

858919

##### Diagnosis.

**Figure 9.**
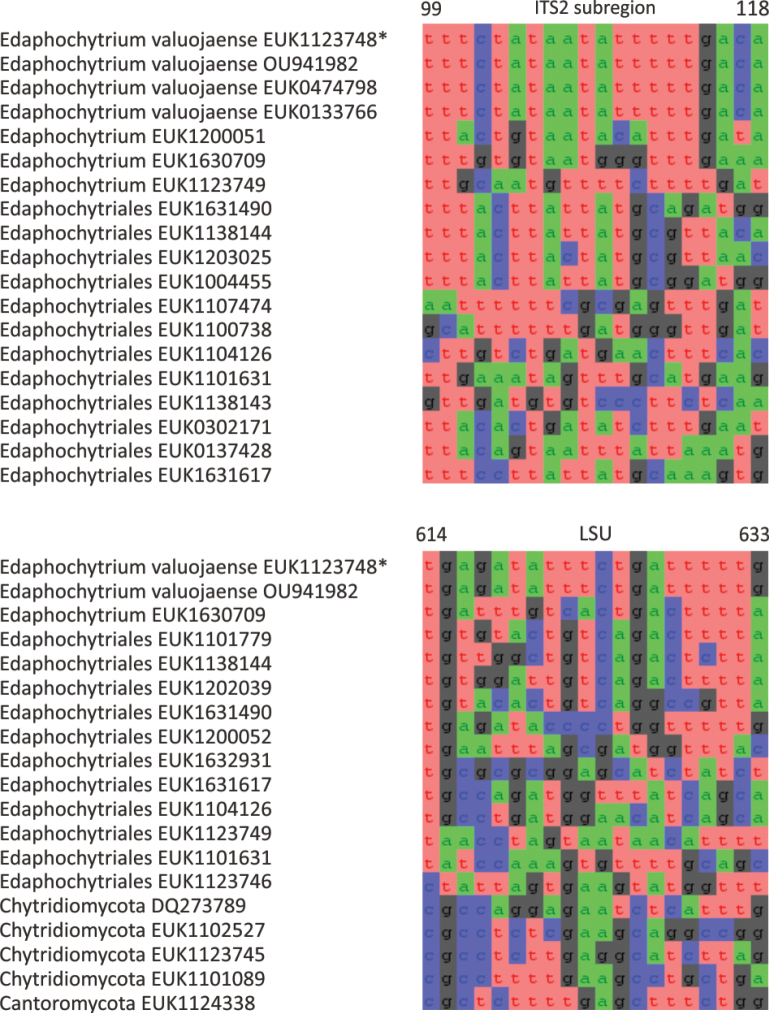
Diagnostic nucleotide sequences of *Edaphochytrium valuojaense* relative to the closest related species in ITS2 and LSU. Numbers indicate positions in the legitype (marked with an asterisk).

Separation from other species of *Edaphochytrium* based on ITS2 (positions 99–118 tttctataatatttttgaca; one mismatch allowed) and LSU (positions 614–633 tgagatatttctgatttttg; one mismatch allowed) as indicated in Fig. 9. Intraspecific variation up to 2.1% in ITS2 and up to 0.6% in LSU. Interspecific distance at least 11.8% in ITS2 and 6.9% in LSU.

##### Type.

Vouchered soil sample TUE001432 (**holotype**); eDNA sequence EUK1123748 = OZ253789 (**legitype**); eDNA sample TUE101432 (**nucleotype**); GSMc plot G4257z, *Salix fragilis* grove soil in Valuoja park, Viljandi, Estonia, 58.3643°N, 25.5859°E.

##### Description.

Other sequences: EUK0133766 (GSMc plot G3522, temperate deciduous forest soil in Pidula, Estonia, 58.4211°N, 22.1522°E); EUK0474798 (*Populus × wettsteinii* plantation soil in Nõgiaru, Estonia, 58.3262°N, 26.5545°E); and OU941982 (grassland soil in Kungsängen, Sweden, 59.837°N, 17.661°E).

##### Etymology.

*Edaphos* (Greek) refers to ground, and *Valuoja* (Estonian) refers to the type locality.

##### Notes.

Found in soil across contrasting habitats in Estonia and Sweden (n = 4 records). GlobalFungi reveals an additional 35 records in European soils and two records in US soils, nearly all in cropland and grassland habitats.

#### Tibetochytriomycetes

Taxon classificationFungiChytridiomycetaChytridiomyceta

Tedersoo class. nov.

88EC7C3A-5484-5D79-92ED-EFCFDC2FA99E

858920

##### Type order.

Tibetochytriales Tedersoo.

##### Diagnosis.

Distinguishable from other fungi based on diagnostic nucleotide signatures in SSU V4 (positions 722–736 in *S. cerevisiae* gtgggttagggatcc or gtgggttagggagct; one mismatch allowed), 5.8S (positions 120–134 in type species and *S. cerevisiae* gctggtattccggcg or tttggtatcccgaag; one mismatch allowed), and LSU D2 (positions 505–619 in type species and 600–614 in *S. cerevisiae* ggcttagctggatac or agcttttgcagggat; two mismatches allowed). Forms a monophyletic, least inclusive clade in Chytridiomycota, covering sequences EUK1186747, EUK1123755, EUK1186750, EUK1102822, and EUK1186746 (Fig. 1).

##### Notes.

Recognized based on eDNA sequences only. Encoded as clade GS43 in EUKARYOME v1.9. Currently harbors Tibetochytriales (ord. nov.). Comprises around five potential species. Detected in soil (91.6% out of the 24 records), once in roots, and once in sediments. Found in tundra to wet tropical biomes across all continents except Antarctica.

#### Tibetochytriales

Taxon classificationFungiChytridiomycetaTibetochytriomycetes

Tedersoo ord. nov.

B1584609-4313-55B9-84D9-CCE38CE198A1

858922

##### Type family.

Tibetochytriaceae Tedersoo.

##### Diagnosis.

Distinguishable from other fungi based on diagnostic nucleotide signatures in SSU V4 (positions 722–736 in *S. cerevisiae* gtgggttagggatcc or gtgggttagggagct; one mismatch allowed), 5.8S (positions 120–134 in type species and *S. cerevisiae* gctggtattccggcg or tttggtatcccgaag; one mismatch allowed), and LSU D2 (positions 505–619 in type species and 600–614 in *S. cerevisiae* ggcttagctggatac or agcttttgcagggat; one mismatch allowed). Forms a monophyletic, least inclusive clade in Tibetochytriomycetes, covering sequences EUK1186747, EUK1123755, EUK1186750, EUK1102822, and EUK1186746 (Fig. 1).

##### Notes.

Recognized based on eDNA sequences only. Currently includes Tibetochytriaceae (fam. nov.) and another potentially family-level group represented by sequences EUK1102822 and EUK1186746 (both forest soil in Puerto Rico).

#### Tibetochytriaceae

Taxon classificationFungiTibetochytriomycetesTibetochytriales

Tedersoo fam. nov.

29B66768-5025-5C88-ACB2-924703FD0A6C

858923

##### Type genus.

*Tibetochytrium* Tedersoo.

##### Diagnosis.

Distinguishable from other fungi based on diagnostic nucleotide signatures in SSU V4 (positions 722–736 in *S. cerevisiae* gtgggttagggatcc; one mismatch allowed), 5.8S (positions 120–134 in type species and *S. cerevisiae* gctggtattccggcg; one mismatch allowed), and LSU D2 (positions 505–619 in type species and 600–614 in *S. cerevisiae* ggcttagctggatac; one mismatch allowed). Forms a monophyletic, least inclusive clade in Tibetochytriales, covering sequences EUK1186747, EUK1123755, and EUK1186750 (Fig. 1).

##### Notes.

Recognized based on eDNA sequences only. Includes *Tibetochytrium* (gen. nov.) and another potentially genus-level group represented by the sequence EUK1186747 (forest soil in Yunnan, China).

#### Tibetochytrium

Taxon classificationFungiTibetochytrialesTibetochytriaceae

Tedersoo gen. nov.

2DE72145-97C3-55DD-8A88-082191CDECB6

858924

##### Type species.

*Tibetochytrium taylorii* Tedersoo.

##### Diagnosis.

**Figure 10.**
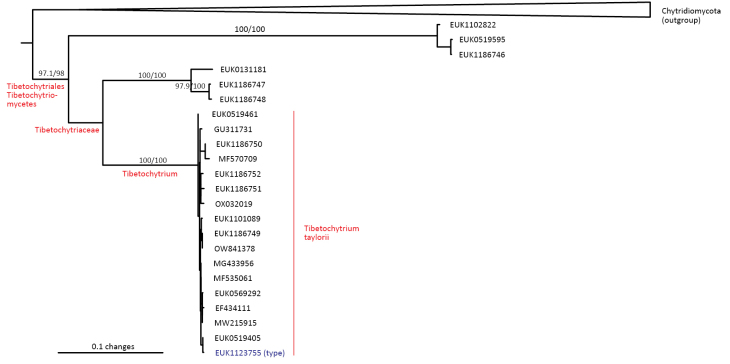
Maximum Likelihood SSU-ITS-LSU phylogram indicating the position of *Tibetochytrium taylorii* within Tibetochytriomycetes, with ultra-rapid bootstrap values indicated (for higher-level classifications only). Other genus-level groups are collapsed. Chytridiomycota spp. were used as an outgroup.

Distinguishable from other fungi based on diagnostic nucleotide signatures in ITS2 (positions 100–119 in type species ttgacagacttacgcgtctt; two mismatches allowed), LSU D2 (positions 552–571 in type species and 547–566 in *S. cerevisiae* aaagtgttatagcttttcat; two mismatches allowed), and SSU V9 (positions 1700–1714 in *S. cerevisiae* caacgaaaatagatt; one mismatch allowed). Forms a monophyletic, least inclusive clade in Tibetochytriaceae, covering sequences EUK1123755 and EUK1186750 (Figs 1, 10).

##### Notes.

Recognized based on eDNA sequences only. Comprises a single species, *Tibetochytrium taylorii* (sp. nov.).

#### Tibetochytrium taylorii

Taxon classificationFungiTibetochytrialesTibetochytriaceae

Tedersoo sp. nov.

4499B23F-FEAB-5A70-AE9A-BE273C68F07B

858925

##### Diagnosis.

**Figure 11.**
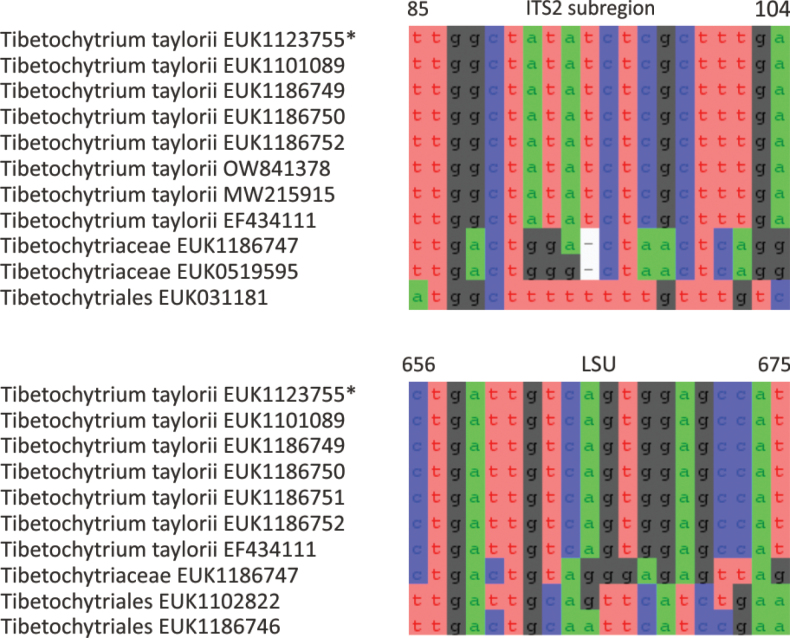
Diagnostic nucleotide sequences of *Tibetochytrium taylorii* relative to the closest related species in ITS2 and LSU. Numbers indicate positions in the legitype (marked with an asterisk).

Separation from other species of *Tibetochytrium* based on ITS2 (positions 85–104 ttggctatatctcgctttga; one mismatch allowed) and LSU (positions 656–675 ctgattgtcagtggagccat; no mismatch allowed) as indicated in Fig. 11. Intraspecific variation up to 3.8% in ITS2 and up to 1.0% in LSU. Interspecific distance > 20% in ITS2.

##### Type.

Vouchered soil sample TUE002260 (**holotype**); eDNA sequence EUK1123755 = OZ253790 (**legitype**); eDNA sample TUE102260 (**nucleotype**); GSMc plot G5283; *Quercus robur* plantation soil in Rahinge, Estonia, 58.3845°N, 26.5943°E).

##### Description.

Other sequences: EUK1186749 (GSMc plot S949; boreal coniferous forest soil in Mt. Mayak, Altai, Russian Federation, 51.0443°N, 82.9694°E); EUK1186750 (GSMc plot S958, temperate broadleaf forest soil in Měšice, Czechia, 50.2006°N, 14.5284°E); EUK1186752 (GSMc plot S966; temperate broadleaf forest soil in Orlík nad Vltavou, Czechia, 49.5002°N, 14.1742°E); OW841378 (unspecified soil in Tianshan Mountains, Uyghuria, China); MW215915 (rhizosphere soil in Lithuania); EF434111 (boreal forest soil in Bonanza Creek, AK, USA); EUK0519405 (GSMc plot S1406, grassland soil in Chuy, Kyrgyzstan, 42.5502°N, 74.5121°E); GU311731 (grassland soil in KS, USA); OX032019 (*Festuca brevipila* roots in temperate grassland, Mallnow, Germany).

##### Etymology.

*Tibet* (Tibetan) refers to the region of the type habitat, and *Taylor* (English) refers to the last name of D. Lee Taylor, who was the first to collect material of this species (EF434111; Taylor et al. 2007).

##### Notes.

The 18 records indicate occurrence mainly in soil (88.9%), with single findings in roots and sediments. Distribution in temperate Eurasia, with two records from North America. The 140 additional GlobalFungi records confirm the soil habitat (97.9%) but extend the distribution to temperate Australia, New Zealand, and Patagonia.

#### Tropicochytriomycetes

Taxon classificationFungiChytridiomycetaChytridiomyceta

Tedersoo class. nov.

C9A27E99-923A-52A8-9A23-890C55A5F4AF

858926

##### Type order.

Tropicochytriales Tedersoo.

##### Diagnosis.

Distinguishable from other fungi based on diagnostic nucleotide signatures in SSU V4 (positions 841–850 in *S. cerevisiae* tccgggracc; no mismatch allowed) and LSU D2 (positions 598–607 in the type species and 592–601 in *S. cerevisiae* agcagcgctg; one mismatch allowed). Forms a monophyletic, least inclusive clade in Chytridiomycota, covering sequences EUK1102342, EUK1186762, EUK1100009, EUK1186758, EUK0519487, EUK1186756, and EUK1102527 (Fig. 1).

##### Notes.

Recognized based on eDNA sequences only. Encoded as clade GS60 in EUKARYOME v1.9. Currently harbors Tropicochytriales (ord. nov.). Comprises potentially around 220–230 species. Detected in soil (98.0% out of the 299 records) and sediments (2.0%). Found in warm temperate to tropical biomes across all continents (except Antarctica), especially in neotropical habitats (46.3% of records). Only 3.0% of records originate from cool temperate localities (in Europe).

#### Tropicochytriales

Taxon classificationFungiChytridiomycetaTropicochytriomycetes

Tedersoo ord. nov.

862DE849-425A-5751-8C0E-005AB0B227E3

858927

##### Type family.

Tropicochytriaceae Tedersoo.

##### Diagnosis.

Distinguishable from other fungi based on diagnostic nucleotide signatures in SSU V4 (positions 841–850 in *S. cerevisiae* tccgggracc; no mismatch allowed) and LSU D2 (positions 598–607 in the type species and 592–601 in *S. cerevisiae* agcagcgctg; one mismatch allowed). Forms a monophyletic, least inclusive clade in Tropicochytriomycetes, covering sequences EUK1102342, EUK1186762, EUK1100009, EUK1186758, EUK0519487, EUK1186756, and EUK1102527 (Fig. 1).

##### Notes.

Recognized based on eDNA sequences only. Currently includes Tropicochytriaceae (fam. nov.).

#### Tropicochytriaceae

Taxon classificationFungiTropicochytriomycetesTropicochytriales

Tedersoo fam. nov.

E98E6ABF-0237-5E69-BFD6-009170375C7B

858929

##### Type genus.

*Tropicochytrium* Tedersoo.

##### Diagnosis.

Distinguishable from other fungi based on diagnostic nucleotide signatures in SSU V4 (positions 841–850 in *S. cerevisiae* tccgggracc; no mismatch allowed) and LSU D2 (positions 598–607 in the type species and 592–601 in *S. cerevisiae* agcagcgctg; one mismatch allowed). Forms a monophyletic, least inclusive clade in Tropicochytriales, covering sequences EUK1102342, EUK1186762, EUK1100009, EUK1186758, EUK0519487, EUK1186756, and EUK1102527 (Fig. 1).

##### Notes.

Recognized based on eDNA sequences only. Includes *Tropicochytrium* (gen. nov.) and other potentially genus-level groups represented by sequences EUK1102342, EUK1186762, EUK1102527, and EUK1100009 (all forest soil in Puerto Rico).

#### Tropicochytrium

Taxon classificationFungiTropicochytrialesTropicochytriaceae

Tedersoo gen. nov.

B5DD887F-EC7E-5FD6-8490-69B66C9792E2

858930

##### Type species.

*Tropicochytrium toronegroense* Tedersoo.

##### Diagnosis.

**Figure 12.**
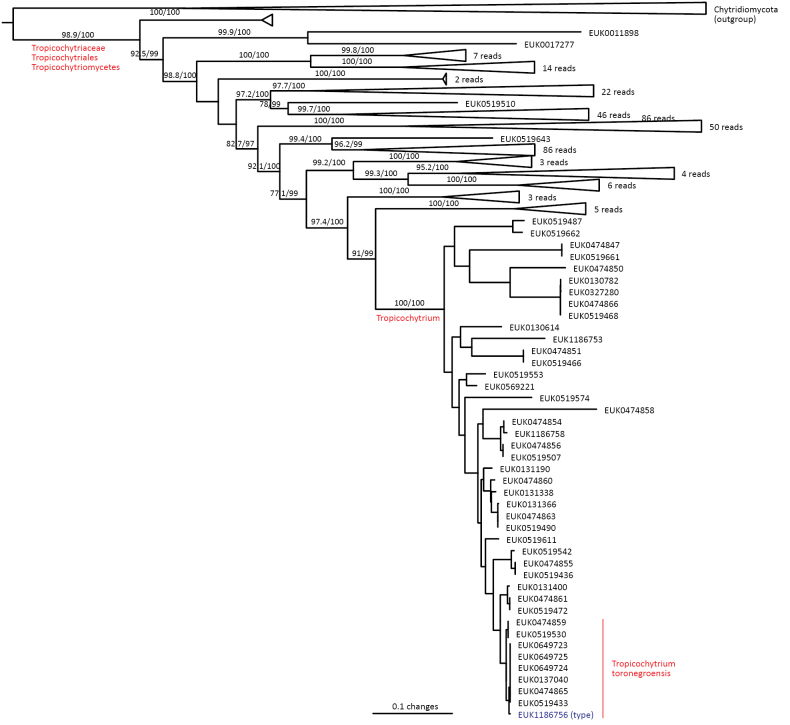
Maximum Likelihood SSU-ITS-LSU phylogram indicating the position of *Tropicochytrium toronegroense* within Tropicochytriomycetes, with ultra-rapid bootstrap values indicated (for higher-level classifications only). Other genus-level groups are collapsed. Chytridiomycota spp. were used as an outgroup.

Distinguishable from other fungi based on diagnostic nucleotide signature in ITS2 (positions 108–127 in type species ctcgtggtccgcaaggcttt; one mismatch allowed) and LSU D2 (positions 557–577 in type species and 549–569 in *S. cerevisiae* agtttatagcctccggtcctg; one mismatch allowed). Forms a monophyletic, least inclusive clade in Tropicochytriaceae, covering sequences EUK1186758, EUK0519487, and EUK1186756 (Figs 1, 12).

##### Notes.

Recognized based on eDNA sequences only. Comprises potentially 20–25 species represented by sequences EUK0519487 (forest soil in the Philippines), EUK1186758 (forest soil in Guadeloupe), EUK1186753 (forest soil in Puerto Rico), and EUK0131338 (grassland soil in Colombia).

#### Tropicochytrium toronegroense

Taxon classificationFungiTropicochytrialesTropicochytriaceae

Tedersoo sp. nov.

BC7695C5-B463-568B-9562-2A35EEAE9C2D

858931

##### Diagnosis.

**Figure 13.**
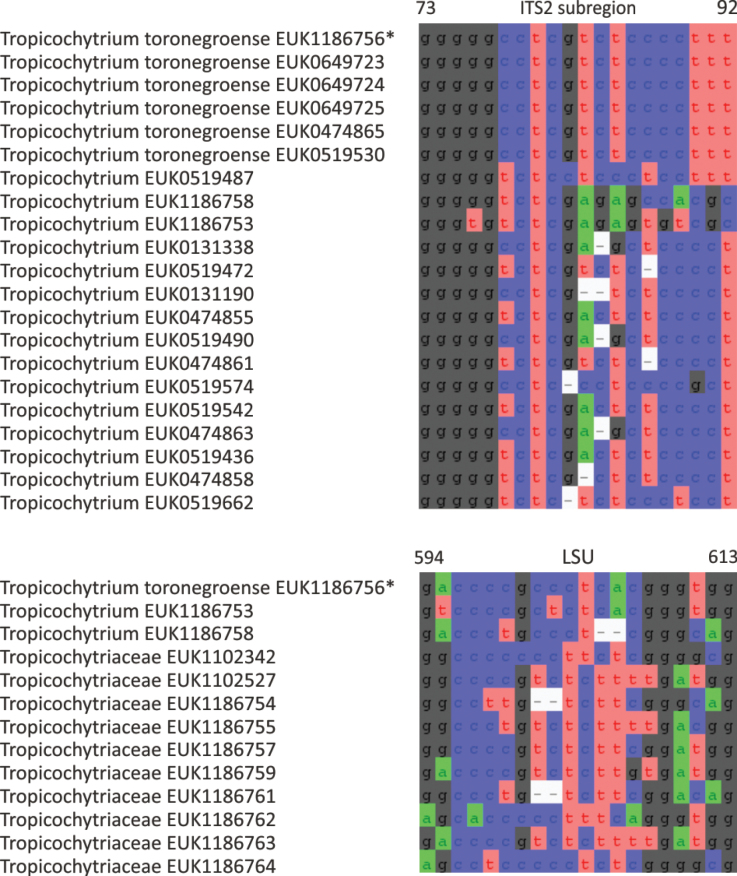
Diagnostic nucleotide sequences of *Tropicochytrium toronegroense* relative to the closest related species in ITS2 and LSU. Numbers indicate positions in the legitype (marked with an asterisk).

Separation from other species of *Tropicochytrium* based on ITS2 (positions 182–201 gggggcctcgtctccccttt; one mismatch allowed) and LSU D2 (positions 536–555 gaccccgccctcacgggtgg; no mismatch allowed) as indicated in Fig. 13. Intraspecific variation up to 2.1% in ITS2. Interspecific distance at least 3.1% in ITS2.

##### Type.

Vouchered soil sample TUE002012 (**holotype**); eDNA sequence EUK1186756 = OZ253791 (**legitype**); eDNA sample TUE102012 (**nucleotype**); GSMc plot G5035; tropical rainforest soil in Toro Negro, Puerto Rico, 18.1770, –66.4884.

##### Description.

Other sequences: EUK0649723 (GSMc plot MX35, *Pinus chiapensis*-dominated tropical forest, Mecacalvo, Veracruz, Mexico, 19.7760, –97.1016); EUK0649724 (GSMc plot S914, tropical forest in Lagos de Monte Bello, Chiapas, Mexico, 16.1004, –91.6871); EUK0649725 (GSMc plot G5037, tropical forest in Maricao, Puerto Rico, 18.1450, –66.9669); EUK0137040, EUK0474865 and EUK0519433 (all GSMc plot S381, tropical forest soil in Col Palmarena, Costa Rica, 10.2211, –84.5992); and EUK0474859 and EUK0519530 (both GSMc plot JYK042, tropical forest soil in Barclayville, Liberia, 4.6777°N, 8.1230°E).

##### Etymology.

*Tropica* (Greek) refers to the tropics, where the genus mainly occurs; *Toro Negro* (Spanish) refers to the type locality.

##### Notes.

Found in soil in tropical rainforest habitats of Central America and West Africa (six localities). There are no additional GlobalFungi records.

#### Monoblepharomycota

Taxon classificationFungiFungiChytridiomyceta

Doweld, Prosyllabus Tracheophytorum, Tentamen systematis plantarum vascularium (Tracheophyta): LXXVII (2001)

3122B554-E8DA-561A-AC34-5C45238AC500

90752

##### Type class.

Monoblepharidomycetes J.H. Schaffner.

##### Description.

As in Schaffner (1909).

##### Notes.

Monoblepharomycota currently harbors Hyaloraphidiomycetes, Monoblepharidomycetes, and Algovoracomycetes (class. nov.).

#### Algovoracomycetes

Taxon classificationFungiChytridiomycetaMonoblepharomycota

Tedersoo & Y. Ding class. nov.

1EB976B9-7454-50A9-BFEA-EF43903308C3

858932

##### Type order.

Algovoracales Tedersoo & Y. Ding.

##### Diagnosis.

Distinguishable from other fungi based on a diagnostic nucleotide signature in SSU V5 (positions 696–714 in *S. cerevisiae* tctttctttctggggaacc or ycttttcttttggggaacc; no mismatch allowed). Forms a monophyletic, least inclusive clade in Monoblepharomycota, covering sequences MF163176, OQ702880, EF024210, OQ687303, OQ687304, OQ687305, OQ687310, OQ687311, EUK1216850, DQ244008, UDB014650, EUK1124454, EUK1216854, EUK1216849, and OQ687309 (Fig. 1).

##### Notes.

Encoded as clade GS13 in EUKARYOME v1.9. Algovoracomycetes currently harbors Algovoracales (ord. nov.), Solivoracales (ord. nov.), and a potential order-level group represented by the sequence OQ687304 (lake water in MI, USA). Comprises potentially 130–160 species. Detected in soil (65.0% out of 303 records), water (20.5%), sediment (13.2%), and algae (1.3%). Algovoracomycetes includes algal parasites, but it remains unknown if this is the most common trophic strategy or characteristic of the order Algovoracales. Members of Algovoracomycetes have been recorded from high arctic to hot tropical biomes across all continents, including Antarctica.

#### Algovoracales

Taxon classificationFungiMonoblepharomycotaAlgovoracomycetes

Tedersoo & Y. Ding ord. nov.

6A8FB21D-8A08-5468-9503-1C486E4E738F

858933

##### Type family.

Algovoracaceae Tedersoo & Y. Ding.

##### Diagnosis.

Distinguishable from other fungi based on diagnostic nucleotide signatures in 5.8S-ITS2 (positions starting from 150 in type species and 154 in *S. cerevisiae* gtgaaacctcctcaa; one mismatch allowed) and from other groups of Monoblepharomycota in SSU V7 (positions 1485–1494 in *S. cerevisiae* acgagtatat; one mismatch allowed). Forms a monophyletic, least inclusive clade in Algovoracomycetes, covering sequences MF163176, OQ702880, EF024210, and OQ687303 (Fig. 1).

##### Notes.

Currently includes Algovoracaceae (fam. nov.).

#### Algovoracaceae

Taxon classificationFungiAlgovoracomycetesAlgovoracales

Tedersoo & Y. Ding fam. nov.

FBFF4A6C-9250-540F-9707-D6CD24A575AC

858934

##### Type genus.

*Algovorax* Tedersoo & Y. Ding.

##### Diagnosis.

Distinguishable from other fungi based on diagnostic nucleotide signatures in 5.8S-ITS2 (positions starting from 150 in type species and 154 in *S. cerevisiae* gtgaaacctcctcaa; one mismatch allowed) and from other groups of Monoblepharomycota in SSU V7 (positions 1485–1494 in *S. cerevisiae* acgagtatat; one mismatch allowed). Forms a monophyletic, least inclusive clade in Algovoracales, covering sequences MF163176, OQ702880, EF024210, and OQ687303 (Fig. 1).

##### Notes.

Includes the genus *Algovorax* (gen. nov.).

#### Algovorax

Taxon classificationFungiAlgovoracalesAlgovoracaceae

Tedersoo & Y. Ding gen. nov.

C908FF2E-2960-55D5-A3D5-3E5EFE4A6530

858935

##### Type species.

*Algovorax scenedesmi* (Fott) Tedersoo & Y. Ding.

##### Description.

Thallus monocentric, consisting of extramatrical, inoperculate, spherical to oval sporangium, and intramatrical spherical apophysis. Rhizoids absent. Zoospores spherical, thin-walled. Resting spores spherical, thick-walled, arising from the extramatrical sporangium. Parasitic on green algae.

##### Diagnosis.

**Figure 14.**
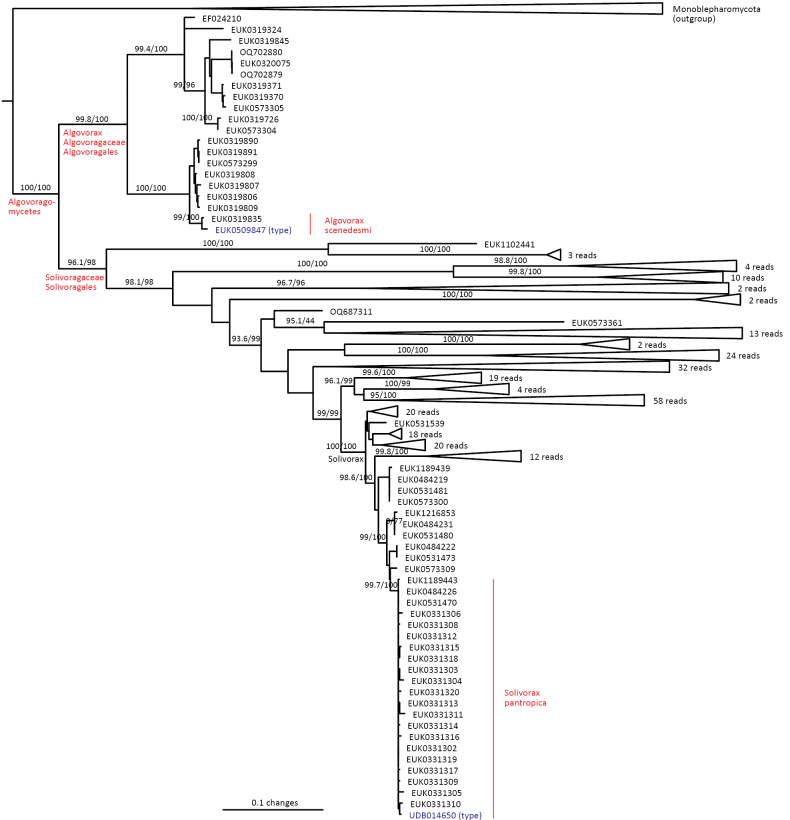
Maximum Likelihood SSU-ITS-LSU phylogram indicating the position of *Algovorax scenedesmi* and *Solivorax pantropicus* within Algovoracomycetes, with ultra-rapid bootstrap values indicated (for higher-level classifications mainly). Other genus-level groups are collapsed. Monoblepharomycota spp. were used as an outgroup.

Distinguishable from other genera of Monoblepharomycota by an intramatrical spherical apophysis and inoperculate sporangium. Distinguishable from other fungi based on diagnostic nucleotide signatures in 5.8S-ITS2 (positions starting from 150 in type species and 154 in *S. cerevisiae* gtgaaacctcctcaa; one mismatch allowed) and from other groups of Monoblepharomycota in SSU V7 (positions 1485–1494 in *S. cerevisiae* acgagtatat; one mismatch allowed). Forms a monophyletic, least inclusive clade in Algovoracaceae, covering sequences MF163176, OQ702880, EF024210, and OQ687303 (Figs 1, 14).

##### Notes.

There are potentially around 6–8 species in *Algovorax* based on ITS sequences, with examples including taxa represented by sequences OQ702880 (algal sample in MI, USA), EUK0319806 (lake sediment in Estonia), EUK0319324 (river sediment in Italy), EUK0319845, and EUK0320075 (both lake sediment in Germany).

#### Algovorax scenedesmi

Taxon classificationFungiAlgovoracalesAlgovoracaceae

(Fott) Tedersoo & Y. Ding comb. nov.

4BE03F6F-92B3-59DE-8BA5-A5FA42E7BA5F

858939

##### Basionym.

*Phlyctidium scenedesmi* Fott [480416].

##### Synonym.

*Rhizophydium scenedesmi* (Fott) Karling [480758].

##### Diagnosis.

**Figure 15.**
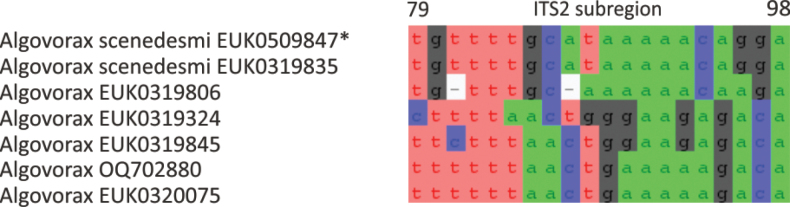
Diagnostic nucleotide sequences of *Algovorax scenedesmi* relative to the closest related species in ITS2. Numbers indicate positions in the legitype (marked with an asterisk).

Separation from species of *Phlyctidium* and *Rhizophydium* based on the lack of rhizoids, large sporangium (5–8 µm), and thin-walled zoospores. Distinguishable from other fungi based on a diagnostic nucleotide signature in ITS2 (positions 79–98 tgttttgcataaaaacagga; one mismatch allowed) as indicated in Fig. 15. Intraspecific variation up to 1.7% in ITS2. Interspecific distance at least 6.7% in ITS2.

##### Type.

Species description based on illustrations in Fott (1967:100)(**holotype**); parasitized algal sample CCTCC M2015486 (**epitype**), eDNA sequences MF163176 (SSU) and EUK0509847 = OZ253793 (ITS) obtained from the epitype; culture EPG01, freshwater pond algae in Chenghai, Yunnan, China (26.38°N, 100.41°E).

##### Description.

As in Fott (1967). Other sequence: EUK0319835 (FunAqua sediment sample W0265; Malinówka river in Krzesławicka, Poland, 49.9864°N, 20.0136°E).

##### Etymology.

*Algovorax* is derived from the Latin words *algos* (algae) and *vorare* (to devour), referring to algae eaters following the parasitic habit characteristic of the type species.

##### Notes.

An old species resurrected by identified specimens and DNA sequences. The eDNA sequence EUK0319835 from Poland provides an additional link between the holotype description from Czechia and the epitype from China. There are no additional records in GlobalFungi. Algal hosts besides *Scenedesmus* spp. include *Chlorococcum* spp. and *Graesiella* sp. (Ding et al. 2018).

#### Solivoracales

Taxon classificationFungiMonoblepharomycotaAlgovoracomycetes

Tedersoo ord. nov.

7CB6B202-99DA-52B3-BA5A-C25BE06DF806

858943

##### Type family.

Solivoracaceae Tedersoo.

##### Diagnosis.

Distinguishable from other fungi based on a diagnostic nucleotide signature in LSU D2 (positions 694–703 in type species and 596–605 in *S. cerevisiae* ctaacgtgct or cctttgtgct; one mismatch allowed). Forms a monophyletic, least inclusive clade in Algovoracomycetes, covering sequences OQ687305, OQ687310, OQ687311, EUK1216850, DQ244008, UDB014650, EUK1124454, EUK1216854, EUK1216849, and OQ687309 (Fig. 1).

##### Notes.

Recognized based on eDNA sequences only. Currently includes Solivoracaceae (fam. nov.).

#### Solivoracaceae

Taxon classificationFungiAlgovoracomycetesAlgovoracomycetes

Tedersoo fam. nov.

6DD4E2A7-6002-570C-B923-F64DED31CFC5

858944

##### Type genus.

*Solivorax* Tedersoo.

##### Diagnosis.

Distinguishable from other fungi based on diagnostic nucleotide signatures in ITS2 (positions in type species gctaagttta; two mismatches allowed) and LSU D2 (positions 694–703 in type species and 596–605 in *S. cerevisiae* ctaacgtgct; one mismatch allowed). Forms a monophyletic, least inclusive clade in Solivoracales, covering sequences OQ687305, OQ687310, OQ687311, EUK1216850, DQ244008, UDB014650, EUK1124454, EUK1216854, EUK1216849, and OQ687309 (Fig. 1).

##### Notes.

Recognized based on eDNA sequences only. Includes *Solivorax* (gen. nov.) and other potentially genus-level groups represented by sequences OQ687305 (lake water in MI, USA), OQ687310 (lake water in MI, USA), OQ687311 (lake water in MI, USA), EUK1216850 (lake sediment in Benin), and DQ244008 (lake water in France).

#### Solivorax

Taxon classificationFungiAlgovoracomycetesSolivoracaceae

Tedersoo gen. nov.

4BAB13E3-2D14-5F27-8C95-563FCC10068D

858945

##### Type species.

*Solivorax pantropicus* Tedersoo.

##### Diagnosis.

Distinguishable from other fungi based on a diagnostic nucleotide signature in ITS2 (positions 85–102 in type species gtaccgctaagtttaagc; one mismatch allowed). Forms a monophyletic, least inclusive clade in Solivoracaceae, covering sequences UDB014650, EUK1124454, EUK1216854, EUK1216849, and OQ687309 (Figs 1, 14).

##### Notes.

Recognized based on eDNA sequences only. Comprises about 60 species represented by sequences EUK1124454 (forest soil in Estonia), EUK1216854 (forest soil in Czechia), EUK1216849 (wetland soil in Estonia), OQ687309 (lake water in MI, USA), EUK0484219 (forest soil in Thailand), KX514861 (rainwater in China), EUK0331291 (woodland soil in Brazil), EUK0331301 (forest soil in Czechia), and EUK0484210 (grassland soil in Estonia).

#### Solivorax pantropicus

Taxon classificationFungiAlgovoracomycetesSolivoracaceae

Tedersoo sp. nov.

24CB805A-5177-5C51-9CD4-DB95B1B27B14

858946

##### Diagnosis.

**Figure 16.**
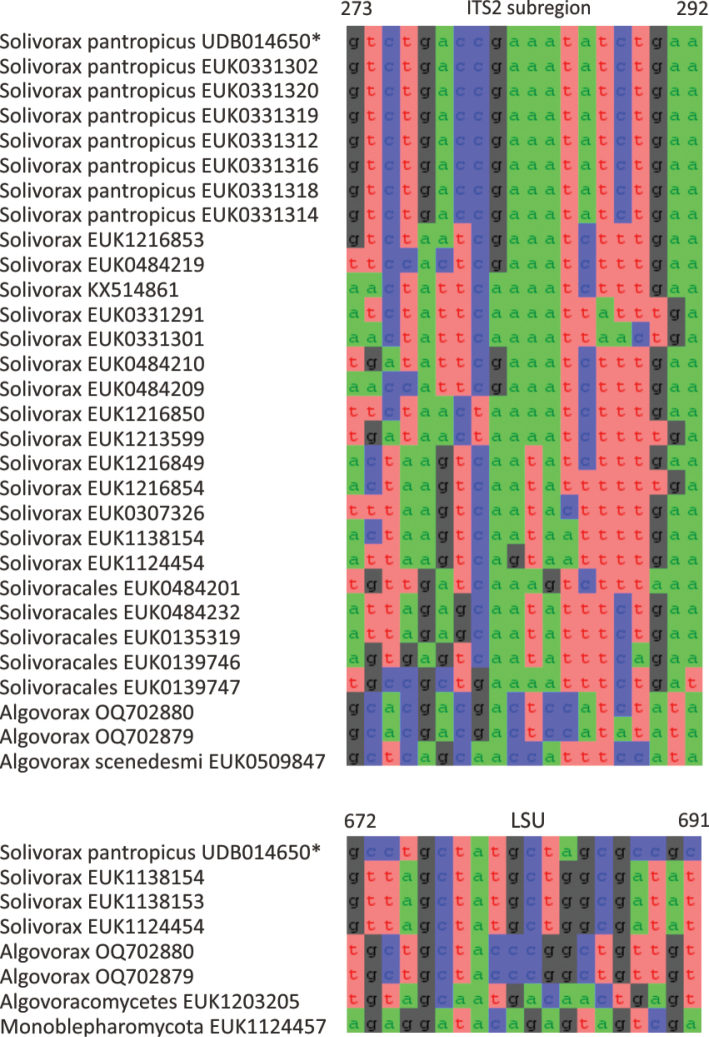
Diagnostic nucleotide sequences of *Solivorax pantropicus* relative to the closest related species in ITS2 and LSU. Numbers indicate positions in the legitype (marked with an asterisk).

Separation from other species of *Solivorax* based on ITS2 (positions 273–292 gtctgaccgaaatatctgaa; one mismatch allowed) and LSU D2 (positions 672–691 gcctgctatgctagcgccgc; one mismatch allowed) as indicated in Fig. 16. Intraspecific variation up to 2.4% in ITS2. Interspecific distance at least 8.2% in ITS2 and 6.2% in LSU.

##### Type.

Vouchered soil sample TUE000167 (**holotype**); eDNA sequence UDB014650 = OZ253792 (**legitype**); eDNA sample TUE100167 (**nucleotype**); GSMc plot G2750, tropical forest soil in Douglas Hot Springs, NT, Australia, –13.7655, 131.4395.

##### Description.

Other sequences: EUK0484226 (GSMc plot S1278, *Eucalyptus* plantation soil near Durban, South Africa, –29.7502, 30.7419); EUK0331319 (GSMc plot G5027, subtropical swamp forest soil in Deweyville, LO, USA, 30.3076°N, 93.7304°E); EUK0331320 (GSMc plot S915, tropical dry forest soil in Rancho Calimayor, Mexico, 16.5461, –93.8828); EUK0331312 (GSMc plot G5687, tropical garden soil in Kakamega, Kenya, 0.2866°N, 34.7673°E); EUK0331316 (GSMc plot JYK054, *Eucalyptus* plantation soil in Bome, Liberia, 6.5245, –10.8400); EUK0331318 (GSMc plot S1267, tropical rainforest soil in Khong Ngam, Thailand, 19.6691°N, 99.8199°E); and EUK0331314 (GSMc plot G5068, tropical rainforest soil in Quixada, Brazil, 4.8876, –39.0461).

##### Etymology.

*Solum* and *vorax* (Latin) refer to soil devouring, and *pantropicos* (Greek) refers to widespread distribution across tropical regions.

##### Notes.

Found in tropical and subtropical forest soils worldwide (n = 23 records). The 17 additional GlobalFungi records confirm the tropical distribution and indicate potential colonization of plant roots (2 records).

#### Neocallimastigomycota

Taxon classificationFungiFungiChytridiomyceta

M.J. Powell, Mycological Research 111 (5): 516 (2007)

0EFCBE20-C5D4-5127-852B-555747970FA4

501279

##### Type class.

Neocallimastigomycetes M.J. Powell.

##### Description.

As in Hibbett et al. (2007).

##### Notes.

Currently harbors Neocallimastigomycetes, Aquamastigomycetes (class. nov.), Cantoromastigomycetes (class. nov.), Dobrisimastigomycetes (class. nov.), Palomastigomycetes (class. nov.), Sedimentomastigomycetes (class. nov.), and potentially class-level taxa represented by sequences EUK1173013 (forest soil in Puerto Rico), EUK0534646 (tundra soil in Buryatiya), EUK1191158 (forest soil in Altai Kray, Russian Federation), EUK0534648 (forest soil in Mexico), EUK1200010 (grassland soil in Italy), and EUK0534645 (forest soil in South Africa).

#### Aquamastigomycetes

Taxon classificationFungiChytridiomycetaNeocallimastigomycota

Tedersoo & Esmaeilzadeh-Salestani class. nov.

6D83079E-1F65-5681-AD40-8154F575CB3D

858947

##### Type order.

Aquamastigales Tedersoo & Esmaeilzadeh-Salestani.

##### Diagnosis.

Distinguishable from other fungi based on a diagnostic nucleotide signature in SSU V4 (positions 966–975 gatcaagagc in *S. cerevisiae*; no mismatch allowed). Forms a monophyletic, least inclusive clade in Neocallimastigomycota, covering sequences EUK1102371, EUK1107057, EUK1124848, EUK1138328, EUK1102991, EUK1124847, and EUK0320721 (Fig. 1).

##### Notes.

Recognized based on eDNA sequences only. Encoded as clade GS38 in EUKARYOME v1.9. Currently harbors Aquamastigales (ord. nov.). Comprises 15–20 species. Detected in sediment (72.4% out of 29 records), freshwater (6.9%), and soil (17.2%) samples in tundra to subtropical biomes in Eurasia, North America, and South America. The predominant records from sediments and flooded soils suggest that members of this class are facultative anaerobes.

#### Aquamastigales

Taxon classificationFungiNeocallimastigomycotaAquamastigomycetes

Tedersoo & Esmaeilzadeh-Salestani ord. nov.

8B25E622-15D9-50D7-970A-0CB34ADCCA7F

858948

##### Type family.

Aquamastigaceae Tedersoo & Esmaeilzadeh-Salestani.

##### Diagnosis.

Distinguishable from other fungi based on a diagnostic nucleotide signature in SSU V4 (positions 966–975 gatcaagagc in *S. cerevisiae*; no mismatch allowed). Forms a monophyletic, least inclusive clade in Aquamastigomycetes, covering sequences EUK1102371, EUK1107057, EUK1124848, EUK1138328, EUK1102991, EUK1124847, and EUK0320721 (Fig. 1).

##### Notes.

Recognized based on eDNA sequences only. Currently includes Aquamastigaceae (fam. nov.) and potential family-level taxa represented by sequences EUK1102371 (permafrost sample in Canada), EUK1107057 (lake sediment sample in Sweden), EUK1124848 (forest soil sample in Estonia), EUK1138328 (wastewater sample in Estonia), and EUK1102991 (lake sediment sample in Sweden).

#### Aquamastigaceae

Taxon classificationFungiAquamastigomycetesAquamastigales

Tedersoo & Esmaeilzadeh-Salestani fam. nov.

2AC2D727-6E8D-5414-B1D3-72E58E83161B

858949

##### Type genus.

*Aquamastix* Tedersoo & Esmaeilzadeh-Salestani.

##### Diagnosis.

Distinguishable from other fungi based on a diagnostic nucleotide signature in SSU V4 (positions 966–975 gatcaagagc in *S. cerevisiae*; no mismatch allowed). Forms a monophyletic, least inclusive clade in Aquamastigales, covering sequences EUK1124847 and EUK0320721 (Fig. 1).

##### Notes.

Recognized based on eDNA sequences only. Currently harbors the genus *Aquamastix* (gen. nov.).

#### Aquamastix

Taxon classificationFungiAquamastigalesAquamastigaceae

Tedersoo & Esmaeilzadeh-Salestani gen. nov.

DB2930C4-813E-5C6E-9C1B-8A033D0A419B

858950

##### Type species.

*Aquamastix sanduskyensis* Tedersoo & Esmaeilzadeh-Salestani.

##### Diagnosis.

**Figure 17.**
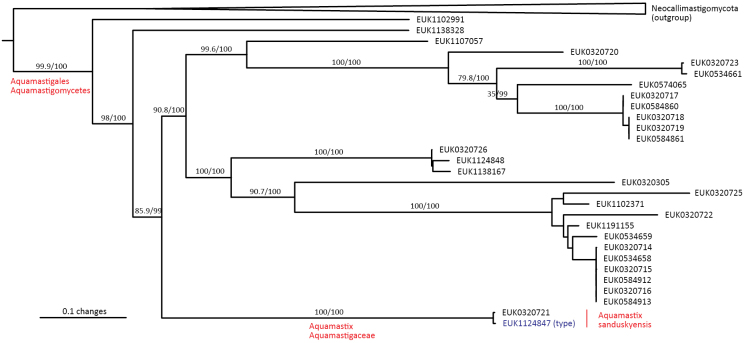
Maximum Likelihood SSU-ITS-LSU phylogram indicating the position of *Aquamastix sanduskyensis* within Aquamastigomycetes, with ultra-rapid bootstrap values indicated. Other genus-level groups are collapsed. Neocallimastigomycota spp. were used as an outgroup.

Distinguishable from other fungi based on diagnostic nucleotide signatures in 5.8S (positions 120–139 ttcctctttg in type species and 118–127 in *S. cerevisiae*; no mismatch allowed), SSU V9 (positions 1685–1699 agtaacttccccttg in *S. cerevisiae*; no mismatch allowed), and LSU D1 (positions 125–139 in type species and 123–137 in *S. cerevisiae* gtgacggtttaactg; two mismatches allowed). Forms a monophyletic, least inclusive clade in Aquamastigaceae, covering sequences EUK1124847 and EUK0320721 (Figs 1, 17).

##### Notes.

Recognized based on eDNA sequences only. Comprises a single species, *Aquamastix sanduskyensis* (sp. nov.).

#### Aquamastix sanduskyensis

Taxon classificationFungiAquamastigalesAquamastigaceae

Tedersoo & Esmaeilzadeh-Salestani sp. nov.

67574F56-DCA4-59EA-8EB1-7CEB8D43DBA9

858951

##### Diagnosis.

**Figure 18.**
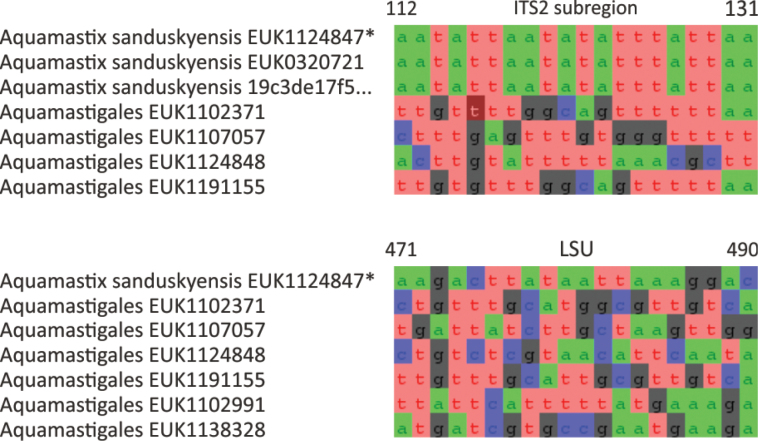
Diagnostic nucleotide sequences of *Aquamastix sanduskyensis* relative to the closest related species in ITS2 and LSU. Numbers indicate positions in the legitype (marked with an asterisk). The full GlobalFungi accession for 19c3de17f5 is 19c3de17f55f5bab0644510c210733d4.

Separation from other species of *Aquamastix* based on ITS2 (positions 112–131 aatattaatatatttattaa; one mismatch allowed) and LSU (positions 471–490 aagacttataattaaaggac; one mismatch allowed) as indicated in Fig. 18. Intraspecific variation up to 1.2% in ITS2. Closest species differ by > 20% in ITS2 and > 10% in LSU.

##### Type.

Vouchered sediment sample TUE031498 (**holotype**); eDNA sequence EUK1124847 = OZ253794 (**legitype**); eDNA sample TUE131498 (**nucleotype**); FunAqua sample W0822s, Sandusky Bay, Lake Erie, OH, USA, 41.45, –82.96.

##### Description.

Other sequences: EUK0320721 (FunAqua sediment sample W0987s, Szczecin Lagoon, Poland, 53.74°N, 14.44°E) and GlobalFungi accession 19c3de17f55f5bab0644510c210733d4 (sediment in Hulun Lake, Inner Mongolia, China, 48.86°N, 117.4°E; two biological samples).

##### Etymology.

*Aqua* (Latin) and *mastix* (Greek) refer to water and *Neocallimastix*, respectively, and *Sandusky* (Wyandot) refers to the cold waters and the part of Lake Erie where the type material originates.

##### Notes.

Found in sediments of lakes in the Northern Hemisphere (n = 3 records).

#### Cantoromastigomycetes

Taxon classificationFungiChytridiomycetaNeocallimastigomycota

Tedersoo class. nov.

72FD6806-E943-56B5-8602-9927B9DA642F

858953

##### Type order.

Cantoromastigales Tedersoo.

##### Diagnosis.

Distinguishable from other fungi based on a diagnostic nucleotide signature in LSU D6 (positions 1759–1778 in type species and 1662–1681 in *S. cerevisiae* ggagacgtcgggdggagccc; no mismatch allowed). Forms a monophyletic, least inclusive clade in Neocallimastigomycota, covering sequences EUK1107297, EUK1201627, OQ687232, OQ687239, EUK1103194, EUK1188586, EUK1152054, EUK1103194, EUK1216883, EUK1124338, EUK1103697, EUK1216882, EUK1124339, EUK1124340, KU359437, EUK1216885, EUK1137900, and EUK1216886 (Fig. 1).

##### Notes.

Recognized based on eDNA sequences only. Encoded as clade GS39 in EUKARYOME v1.9. Currently harbors Cantoromastigales (ord. nov.) and potential order-level groups represented by sequences EUK1107297 (peatland soil in Sweden), EUK1201627 (forest soil in Italy), OQ687232 (lake water in MI, USA), and OQ687239 (unspecified water). Comprises around 130–140 species. Detected in soil (99.0% out of 220 records), water (0.5%), and sediment (0.5%) in tundra to hot tropical biomes across all continents except Antarctica.

#### Cantoromastigales

Taxon classificationFungiNeocallimastigomycotaCantoromastigomycetes

Tedersoo ord. nov.

DB68778F-7C60-5625-9689-4A72C7977263

858955

##### Type family.

Cantoromastigaceae Tedersoo.

##### Diagnosis.

Distinguishable from other fungi based on a diagnostic nucleotide signature in the LSU 5’ end (positions –2–8 in the type species and *S. cerevisiae* acgtggtctc or atatggtctc; no mismatch allowed). Forms a monophyletic, least inclusive clade in Cantoromastigomycetes, covering sequences EUK1152054, EUK1103194, EUK1216883, EUK1124338, EUK1103697, EUK1216882, EUK1124339, EUK1124340, KU359437, EUK1216885, EUK1137900, and EUK1216886 (Fig. 1).

##### Notes.

Recognized based on eDNA sequences only. Currently includes Cantoromastigaceae (fam. nov.) and other potentially order-level groups represented by sequences EUK1152054 (forest soil in New Zealand), EUK1103194 (lake water in Sweden), EUK1216883 (river sediment in Romania), EUK1103697 (forest soil in Puerto Rico), EUK1216882 (forest soil in the Canary Islands), EUK1124339 (grassland soil in Estonia), EUK1124340 (greenhouse soil in Estonia), KU359437 (plantation soil in China), EUK1216885 (forest soil in Estonia), EUK1137900 (urban soil in Estonia), and EUK1216886 (forest soil in Estonia).

#### Cantoromastigaceae

Taxon classificationFungiCantoromastigomycetesCantoromastigales

Tedersoo fam. nov.

2F9B1523-A43A-59DF-B9D5-4A87FB1EA3D2

858956

##### Type genus.

*Cantoromastix* Tedersoo.

##### Diagnosis.

Distinguishable from other fungi based on diagnostic nucleotide signatures in 5.8S (positions 117–136 in type species and *S. cerevisiae* ctttcgggtaayccccggga; one mismatch allowed) and ITS2 (positions 156–170 in type species cgtaaccaaaaggct or cgtaccaratctttt; one mismatch allowed). Forms a monophyletic, least inclusive clade in Cantoromastigales, covering sequences EUK1124338, EUK0136917, EUK0017791, and EUK0523855 (Fig. 1).

##### Notes.

Recognized based on eDNA sequences only. Includes *Cantoromastix* (gen. nov.) and another potentially genus-level group represented by the sequence EUK0523855 (forest soil in FL, USA).

#### Cantoromastix

Taxon classificationFungiCantoromastigalesCantoromastigaceae

Tedersoo gen. nov.

C8743E36-712B-55F4-968E-3424896724DD

858957

##### Type species.

*Cantoromastix holarctica* Tedersoo.

##### Diagnosis.

**Figure 19.**
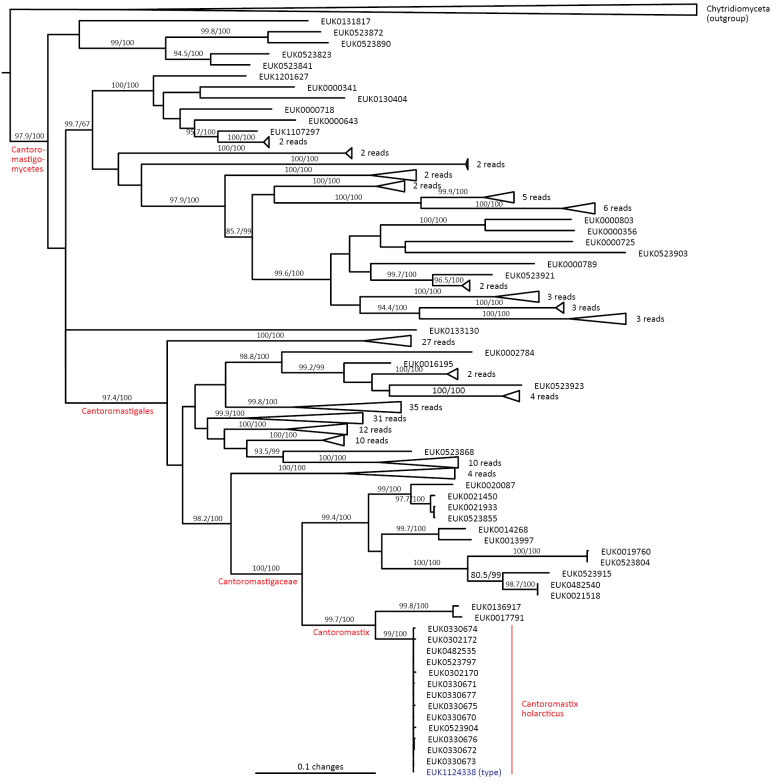
Maximum Likelihood SSU-ITS-LSU phylogram indicating the position of *Cantoromastix holarctica* within Cantoromastigomycetes, with ultra-rapid bootstrap values indicated (for higher-level classifications mainly). Other genus-level groups are collapsed. Neocallimastigomycota spp. were used as an outgroup.

Distinguishable from other fungi based on diagnostic nucleotide signatures in ITS2 (positions 123–137 in type species ctagtcatctttaag; two mismatches allowed) and SSU 3’ end (positions 1796–1800 in *S. cerevisiae* and 5 bases of ITS: cattagctta; no mismatch allowed). Forms a monophyletic, least inclusive clade in Cantoromastigaceae, covering sequences EUK1124338, EUK0136917, and EUK0017791 (Figs 1, 19).

##### Notes.

Recognized based on eDNA sequences only. Comprises 2–3 potential species represented by sequences EUK0136917 (forest soil in Costa Rica) and EUK0017791 (forest soil in Guadeloupe).

#### Cantoromastix holarctica

Taxon classificationFungiCantoromastigalesCantoromastigaceae

Tedersoo sp. nov.

EF7FD2A0-32A5-58FD-9811-37C6BD3A9DBB

858959

##### Diagnosis.

**Figure 20.**
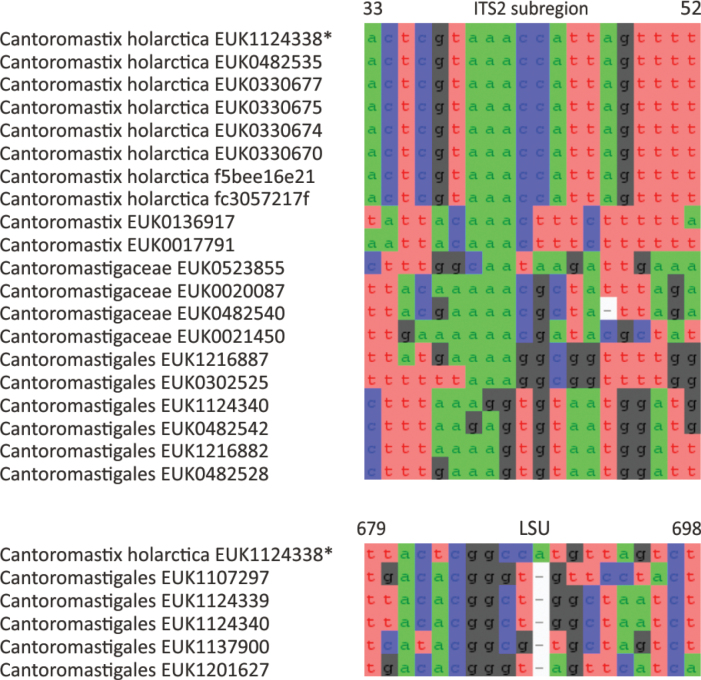
Diagnostic nucleotide sequences of *Cantoromastix holarctica* relative to the closest related species in ITS2 and LSU. Numbers indicate positions in the legitype (marked with an asterisk).

Separation from other species of *Cantoromastix* based on ITS2 (positions 33–52 actcgtaaaccattagtttt; one mismatch allowed) and LSU D2 (positions 679–698 ttactcggccatgttagtct; one mismatch allowed) as indicated in Fig. 20. Intraspecific variation up to 1.1% in ITS2. Interspecific distance > 20% in ITS2 and > 15% in LSU.

##### Type.

Vouchered soil sample TUE000915 (**holotype**); eDNA sequence EUK1124338 = OZ253795 (**legitype**); eDNA sample TUE100915 (**nucleotype**); GSMc plot S383, urban park soil in Tartu, Estonia, 58.3889°N, 26.7031°E.

##### Description.

Other sequences: EUK0482535 (temperate fallow soil in Haava, Estonia, 58.4611°N, 26.7738°E); EUK0330677 (*Populus tremula* forest soil in Vasula, Estonia, 58.4699°N, 26.7266°E); and EUK0330675 (GSMc plot G5923, *Malus domestica* cropland soil in Kalnabeites, Latvia, 57.1333°N, 24.8567°E); EUK0330674 (GSMc plot G5920, temperate grassland soil in Viinamärdi, Estonia, 58.2497°N, 26.5394°E); EUK0330670 (temperate grassland soil in Kihnu, Estonia, 58.1467°N, 23.9852°E); EUK0330673 (GSMc plot G5930, *Zea mays* cropland soil in Saulkalne, Latvia, 56.8442°N, 24.4072°E); and EUK0330671 (coppiced fallow soil in Lombi, Estonia, 58.4551°N, 26.7451°E).

##### Etymology.

*Cantor* (Latin) refers to singers, which reflects the origin of the type material at the song festival grounds, and *mastix* refers to *Neocallimastix*; and *holos* and *arcticos* (Greek) refer to the entire northern (holarctic) distribution of the type species.

##### Notes.

Found in eight soil samples in Estonia and Latvia. GlobalFungi records (n = 263) suggest a broader distribution in temperate North American and East Asian soils and a preference for treeless habitats.

#### Dobrisimastigomycetes

Taxon classificationFungiChytridiomycetaNeocallimastigomycota

Tedersoo class. nov.

AAF9817A-5E40-59C9-9E5D-2E966D2EDE8E

858961

##### Type order.

Dobrisimastigales Tedersoo.

##### Diagnosis.

Distinguishable from other fungi based on a diagnostic nucleotide signature in LSU D1 (positions 124–138 in the type species and 122–136 in *S. cerevisiae* tgggtaggttacctg; three mismatches allowed). Forms a monophyletic, least inclusive clade in Neocallimastigomycota, covering sequences EUK1189296, EUK1138904, EUK0534669, EUK0534670, and EUK0534680 (Fig. 1).

##### Notes.

Recognized based on eDNA sequences only. Labelled as clade GS93B in EUKARYOME v1.9. Currently harbors Dobrisimastigales (ord. nov.). Comprises 10–12 species. Detected in soil (91.7% out of 36 records) and sediments (8.3%) in tundra to tropical biomes across all continents except Antarctica.

#### Dobrisimastigales

Taxon classificationFungiNeocallimastigomycotaDobrisimastigomycetes

Tedersoo ord. nov.

1A475CDE-D4F6-5629-8498-3968787F95B5

858963

##### Type family.

Dobrisimastigaceae Tedersoo.

##### Diagnosis.

Distinguishable from other fungi based on diagnostic nucleotide signatures in SSU V4 (positions 700–719 in *S. cerevisiae* ctggtgaatcatcgtgctct; one mismatch allowed) and LSU D1 (positions 124–138 in the type species and 122–136 in *S. cerevisiae* tgggtaggttacctg; two mismatches allowed). Forms a monophyletic, least inclusive clade in Dobrisimastigomycetes, covering sequences EUK1189296, EUK1138904, EUK0534669, EUK0534670, and EUK0534680 (Fig. 1).

##### Notes.

Recognized based on eDNA sequences only. Currently includes Dobrisimastigaceae (fam. nov.).

#### Dobrisimastigaceae

Taxon classificationFungiDobrisimastigomycetesDobrisimastigales

Tedersoo fam. nov.

37CDD1AD-61AB-5A4E-8C97-14769B597B86

858964

##### Type genus.

*Dobrisimastix* Tedersoo.

##### Diagnosis.

Distinguishable from other fungi based on diagnostic nucleotide signatures in the ITS2 region (positions 2–18 in type species taaaatrtcacaaccac; three mismatches allowed), SSU V4 (positions 700–719 in *S. cerevisiae* ctggtgaatcatcgtgctct; one mismatch allowed), and LSU D1 (positions 124–138 in the type species and 122–136 in *S. cerevisiae* tgggtaggttacctg; two mismatches allowed). Forms a monophyletic, least inclusive clade in Dobrisimastigales, covering sequences EUK1189296, EUK1138904, EUK0534669, EUK0534670, and EUK0534680 (Fig. 1).

##### Notes.

Recognized based on eDNA sequences only. Comprises *Dobrisimastix* (gen. nov.).

#### Dobrisimastix

Taxon classificationFungiDobrisimastigalesDobrisimastigaceae

Tedersoo gen. nov.

5851DC34-9643-5533-AA1B-B3D9852890C2

858966

##### Type species.

*Dobrisimastix vlkii* Tedersoo.

##### Diagnosis.

**Figure 21.**
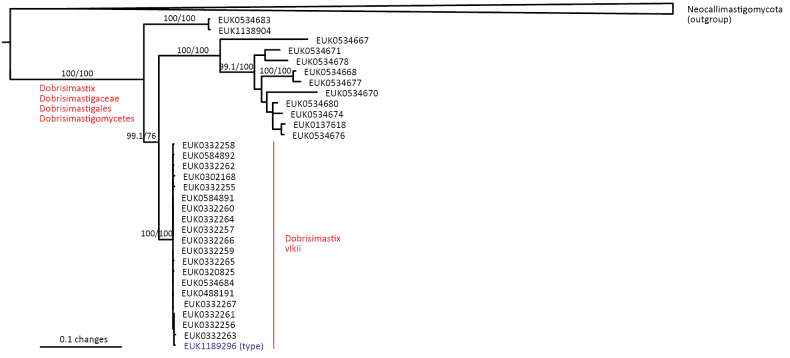
Maximum Likelihood SSU-ITS-LSU phylogram indicating the position of *Dobrisimastix vlkii* within Dobrisimastigomycetes, with ultra-rapid bootstrap values indicated. Other genus-level groups are collapsed. Neocallimastigomycota spp. were used as an outgroup.

Distinguishable from other fungi based on diagnostic nucleotide signatures in ITS2 (positions 2–18 in type species taaaatrtcacaaccac; one mismatch allowed), SSU V4 (positions 700–719 in *S. cerevisiae* ctggtgaatcatcgtgctct; no mismatch allowed), LSU D1 (positions 124–138 in the type species and 122–136 in *S. cerevisiae* tgggtaggttacctg; one mismatch allowed), and LSU D2 (positions 507–526 in type species and 452–471 in *S. cerevisiae* tgtataagaggcttcgcttg; one mismatch allowed). Forms a monophyletic, least inclusive clade in Dobrisimastigaceae, covering sequences EUK1189296, EUK1138904, EUK0534669, EUK0534670, and EUK0534680 (Figs 1, 21).

##### Notes.

Recognized based on eDNA sequences only. Harbors 10–12 potential species represented by sequences EUK1138904 (forest soil in New Zealand), EUK0534669 (forest soil in Guatemala), EUK0534670 (forest soil in Colombia), EUK0534680 (forest soil in Colombia), EUK0534676 (greenhouse soil in Estonia), EUK0534677 (forest soil in Mexico), and EUK0534667 (forest soil in Tanzania).

#### Dobrisimastix vlkii

Taxon classificationFungiDobrisimastigalesDobrisimastigaceae

Tedersoo sp. nov.

F78E705D-5FAE-5C63-B2DD-BA25C603BD81

858968

##### Diagnosis.

**Figure 22.**
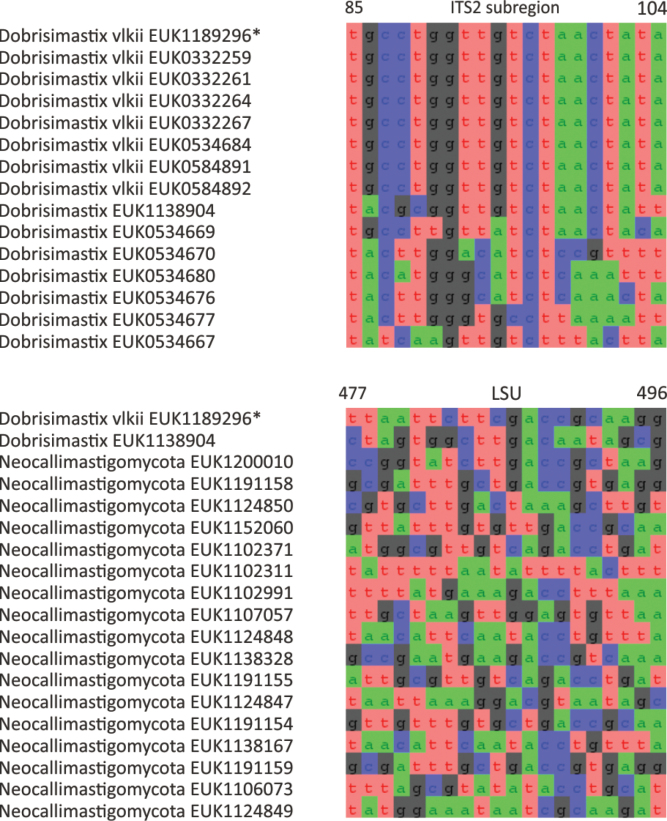
Diagnostic nucleotide sequences of *Dobrisimastix vlkii* relative to the closest related species in ITS2 and LSU. Numbers indicate positions in the legitype (marked with an asterisk).

Separation from other species of *Dobrisimastix* based on ITS2 (positions 85–104 tgcctggttgtctaactata; one mismatch allowed) and LSU D2 (positions 477–496 ttaattcttcgaccgcaagg; one mismatch allowed) as indicated in Fig. 22. Intraspecific variation up to 1.5% in ITS2. Interspecific distance at least 8.4% in ITS2.

##### Type.

Vouchered soil sample TUE003459 (**holotype**); eDNA sequence EUK1189296 = OZ253796 (**legitype**); eDNA sample TUE103459 (**nucleotype**); GSMc plot S961, temperate deciduous forest in Dobříš, Czechia, 49.7776°N, 14.1815°E.

##### Description.

Other sequences: EUK0332259 (GSMc plot S947, boreal forest soil in Malyi Tigirek, Altai, Russian Federation, 51.1247°N, 83.0368°E); EUK0332261 (GSMc plot S149, temperate *Pinus* forest soil in Stanislaus, CA, USA, 37.8138, –119.8926); EUK0332264 (GSMc plot IH.ME29, temperate *Fagus orientalis* forest soil in Mestia, Georgia, 42.9764°N, 42.5429°E); EUK0332267 (GSMc plot G2629, temperate mixed forest soil in Nigula, Estonia, 58.0458°N, 24.7119°E); EUK0534684 (GSMc plot S431, Arctic tundra soil in Toolik Lake, AK, USA, 68.622, –149.5977); EUK0584891 (FunAqua sediment sample W0220s, Triefenbach, Germany, 49.2812°N, 8.1135°E); and EUK0584892 (FunAqua sediment sample W0525s, Novaki, Croatia, 45.6573°N, 15.6345°E).

##### Etymology.

*Dobříš* (Czech) and *mastix* (Greek) refer to the type locality and *Neocallimastix*, respectively, and *Vlk* (Czech) refers to Lukáš Vlk, who collected the type material.

##### Notes.

Found in soil samples (88.9%) and sediments of lakes (11.1%) in tundra to warm temperate forests in the Northern Hemisphere (n = 18 localities). GlobalFungi reveals 227 additional records in soil (91.6%), roots (5.7%), and sediments (1.3%) in Europe, North America, and Asia, with occasional findings from tropical forests in Kenya and South America.

#### Palomastigomycetes

Taxon classificationFungiChytridiomycetaNeocallimastigomycota

Tedersoo class. nov.

BF5B1710-4026-529F-9F06-727027061BDD

858969

##### Type order.

Palomastigales Tedersoo.

##### Diagnosis.

Distinguishable from other fungi based on a diagnostic nucleotide signature in SSU V8 (positions 1664–1678 in *S. cerevisiae* tttagtgaggactcg; one mismatch allowed). Forms a monophyletic, least inclusive clade in Neocallimastigomycota, covering sequences EUK1124846, EUK1123686, EUK0320705, and EUK0320700 (Fig. 1).

##### Notes.

Recognized based on eDNA sequences only. Encoded as clade GS38Y in EUKARYOME v1.9. Currently harbors Palomastigales (ord. nov.). Comprises potentially seven species. Detected in sediment (89.5% out of 19 records) and soil (10.5%) samples in boreal to tropical biomes in Eurasia and North America. Relative commonness in sediments suggests that members of this class are facultative anaerobes.

#### Palomastigales

Taxon classificationFungiNeocallimastigomycotaPalomastigomycetes

Tedersoo ord. nov.

8E313381-A6E2-58B9-94F9-17D790EFCD1F

858970

##### Type family.

Palomastigaceae Tedersoo.

##### Diagnosis.

Distinguishable from other fungi based on diagnostic nucleotide signatures in SSU V8 (positions 1664–1678 in *S. cerevisiae* tttagtgaggactcg; no mismatch allowed) and 5.8S (positions 116–135 in type species and *S. cerevisiae* gcctcccggtattccaggag or gcttcatggtattccgtga; one mismatch allowed). Forms a monophyletic, least inclusive clade in Palomastigomycetes, covering sequences EUK1124846, EUK1123686, EUK0320705, and EUK0320700 (Fig. 1).

##### Notes.

Recognized based on eDNA sequences only. Currently includes Palomastigaceae (fam. nov.).

#### Palomastigaceae

Taxon classificationFungiPalomastigomycetesPalomastigales

Tedersoo fam. nov.

07FBEC08-4A26-5224-B840-BD08BB850074

858971

##### Type genus.

*Palomastix* Tedersoo.

##### Diagnosis.

Distinguishable from other fungi based on diagnostic nucleotide signatures in SSU V9 (positions 1664–1678 in *S. cerevisiae* tttagtgaggactcg; no mismatch allowed) and 5.8S (positions 116–135 in type species and *S. cerevisiae* gcctcccggtattccaggag or gcttcatggtattccgtga; one mismatch allowed). Forms a monophyletic, least inclusive clade in Palomastigales, covering sequences EUK1124846, EUK1123686, EUK0320705, and EUK0320700 (Fig. 1).

##### Notes.

Recognized based on eDNA sequences only. Harbors *Palomastix* (gen. nov.) and potential genus-level taxa represented by sequences EUK0574070 (marine sediment in the Philippines), EUK0137263 (forest soil in Mexico), and EUK1124846 (wasteland soil in Estonia).

#### Palomastix

Taxon classificationFungiPalomastigalesPalomastigaceae

Tedersoo gen. nov.

880F0DA5-7439-5ACD-9D69-7205386B0EC4

858972

##### Type species.

*Palomastix lacustris* Tedersoo.

##### Diagnosis.

**Figure 23.**
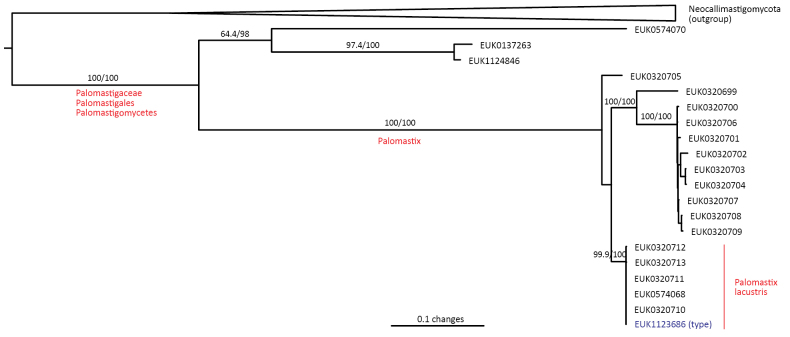
Maximum Likelihood SSU-ITS-LSU phylogram indicating the position of *Palomastix lacustris* within Palomastigomycetes, with ultra-rapid bootstrap values indicated. Other genus-level groups are collapsed. Neocallimastigomycota spp. were used as an outgroup.

Distinguishable from other fungi based on diagnostic nucleotide signatures in 5.8S (positions 116–135 in type species and *S. cerevisiae* gcctcccggtattccaggag; one mismatch allowed), SSU V9 (positions 1691–1710 in *S. cerevisiae* tgttgggctcacgccctcct; one mismatch allowed), and ITS2 (positions 129–148 in type species tctcaagttaagtgattggt; two mismatches allowed). Forms a monophyletic, least inclusive clade in Palomastigaceae, covering sequences EUK1123686, EUK0320705, and EUK0320700 (Figs 1, 23).

##### Notes.

Recognized based on eDNA sequences only. Comprises four potential species represented by sequences EUK0320699 (river sediment in Portugal), EUK0320700 (lake sediment in Estonia), EUK0320701 (lake sediment in Lithuania), EUK0320702 (lake sediment in Spain), and EUK0320705 (lake sediment in Norway).

#### Palomastix lacustris

Taxon classificationFungiPalomastigalesPalomastigaceae

Tedersoo sp. nov.

7151F516-6669-54FC-A9E5-A571EF62631F

858973

##### Diagnosis.

**Figure 24.**
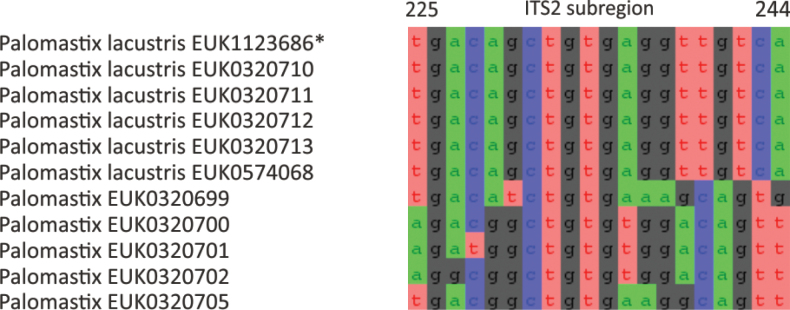
Diagnostic nucleotide sequences of *Palomastix lacustris* relative to the closest related species in ITS2. Numbers indicate positions in the legitype (marked with an asterisk).

Separation from other species of *Palomastix* based on ITS2 (positions 225–244 ctaaaagtcgggtttgattt; one mismatch allowed) and ITS1 (positions 464–483 cagcaggtcttgactgactt; one mismatch allowed) as indicated in Fig. 24. Intraspecific variation up to 1.4% in ITS2. Interspecific distance at least 9.2% in ITS2.

##### Type.

Vouchered sediment sample TUE030088 (**holotype**); eDNA sequence EUK1123686 = OZ253797 (**legitype**); eDNA sample TUE130088 (**nucleotype**); FunAqua lake sediment sample W0021s; Palojärv, Estonia, 58.0830°N, 26.9143°E.

##### Description.

Other sequences: EUK0320710 and EUK0574068 (type locality); EUK0320711, EUK0320713 (FunAqua lake sediment sample in Viitna Pikkjärv, Estonia, 59.4469°N, 26.0107°E); and EUK0320712 (FunAqua lake sediment sample W0992s in Svartkulpen, Norway, 59.9741°N, 10.7373°E).

##### Etymology.

*>Palo* (Estonian) refers to the type locality, Lake Palojärv; *lacustris* refers to habitat in lakes.

##### Notes.

Found in sediment samples in Northern Europe (n = 3). There are no records in GlobalFungi.

#### Sedimentomastigomycetes

Taxon classificationFungiChytridiomycetaNeocallimastigomycota

Tedersoo class. nov.

8156049A-2BED-5A05-B833-C8AC1677D7F6

858975

##### Type order.

Sedimentomastigales Tedersoo.

##### Diagnosis.

Distinguishable from other fungi based on a diagnostic nucleotide signature in SSU V9 (positions 1672–1686 in *S. cerevisiae* ggcctccggattgat; no mismatch allowed). Forms a monophyletic, least inclusive clade in Neocallimastigomycota, covering sequences EUK0319782, EUK0574067, EUK0574066, EUK1152060, and EUK1191154 (Fig. 1).

##### Notes.

Recognized based on eDNA sequences only. Encoded as clade GS38X in EUKARYOME v1.9. Currently harbors Sedimentomastigales (ord. nov.). Comprises 5–6 potential species. Detected in sediments (50% out of 10 records), freshwater (30%), soil (10%), and rotting algae (10%) in tundra to tropical biomes in Eurasia, North America, and South America. The many records from sediments and flooded soils suggest that members of this class are facultative anaerobes.

#### Sedimentomastigales

Taxon classificationFungiNeocallimastigomycotaSedimentomastigomycetes

Tedersoo ord. nov.

250D47DE-7559-50B0-83F1-EAC510C5E3BE

858976

##### Type family.

Sedimentomastigaceae Tedersoo.

##### Diagnosis.

Distinguishable from other fungi based on diagnostic nucleotide signatures in SSU V9 (positions 1672–1686 in *S. cerevisiae* ggcctccggattgat; no mismatch allowed) and LSU D2 (positions 524–538 in the type species and 460–474 in *S. cerevisiae* ttggtattttgggtg; three mismatches allowed). Forms a monophyletic, least inclusive clade in Sedimentomastigomycetes, covering sequences EUK0319782, EUK0574067, EUK0574066, EUK1152060, and EUK1191154 (Fig. 1).

##### Notes.

Recognized based on eDNA sequences only. Currently includes Sedimentomastigaceae (fam. nov.).

#### Sedimentomastigaceae

Taxon classificationFungiSedimentomastigomycetesSedimentomastigales

Tedersoo fam. nov.

055F6432-250F-57D3-BED3-25D94E8A684C

858977

##### Type genus.

*Sedimentomastix* Tedersoo.

##### Diagnosis.

Distinguishable from other fungi based on diagnostic nucleotide signature in SSU V9 (positions 1672–1686 in *S. cerevisiae* ggcctccggattgat; no mismatch allowed) and LSU D2 (positions 524–538 in type species and 460–474 in *S. cerevisiae* ttggtattttgggtg; three mismatches allowed). Forms a monophyletic, least inclusive clade in Sedimentomastigales, covering sequences EUK0319782, EUK0574067, EUK0574066, EUK1152060, and EUK1191154 (Fig. 1).

##### Notes.

Recognized based on eDNA sequences only. Currently harbors *Sedimentomastix* (gen. nov.).

#### Sedimentomastix

Taxon classificationFungiSedimentomastigalesSedimentomastigaceae

Tedersoo gen. nov.

EC39DD3C-A5E4-5017-BB47-4111732DEEF5

858978

##### Type species.

*Sedimentomastix tueriensis* Tedersoo.

##### Diagnosis.

**Figure 25.**
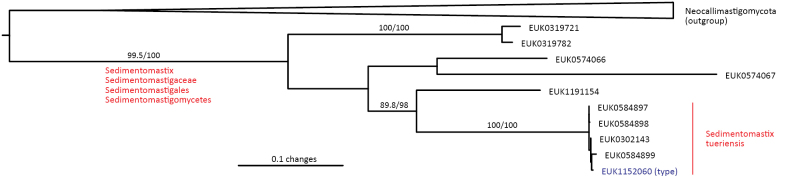
Maximum Likelihood SSU-ITS-LSU phylogram indicating the position of *Sedimentomastix tueriensis* within Sedimentomastigomycetes, with ultra-rapid bootstrap values indicated (for higher-level classifications only). Other genus-level groups are collapsed. Neocallimastigomycota spp. were used as an outgroup.

Distinguishable from other fungi based on diagnostic nucleotide signatures in SSU V9 (positions 1672–1686 in *S. cerevisiae* ggcctccggattgat; no mismatch allowed) and LSU D2 (positions 524–538 in the type species and 460–474 in *S. cerevisiae* ttggtattttgggtg; two mismatches allowed). Forms a monophyletic, least inclusive clade in Sedimentomastigaceae, covering sequences EUK0319782, EUK0574067, EUK0574066, EUK1152060, and EUK1191154 (Figs 1, 25).

##### Notes.

Recognized based on eDNA sequences only. Comprises 5–6 potential species represented by sequences EUK1191154 (rotting algae in Estonia), EUK0319721 (river sediment in Germany), EUK0319782 (lake sediment in Estonia), EUK0574066 (lake sediment in Tibet), and EUK0574067 (river sediment in Brazil).

#### Sedimentomastix tueriensis

Taxon classificationFungiSedimentomastigalesSedimentomastigaceae

Tedersoo sp. nov.

81A33060-ABF4-555C-81C3-ACA7380CDD24

858980

##### Diagnosis.

**Figure 26.**
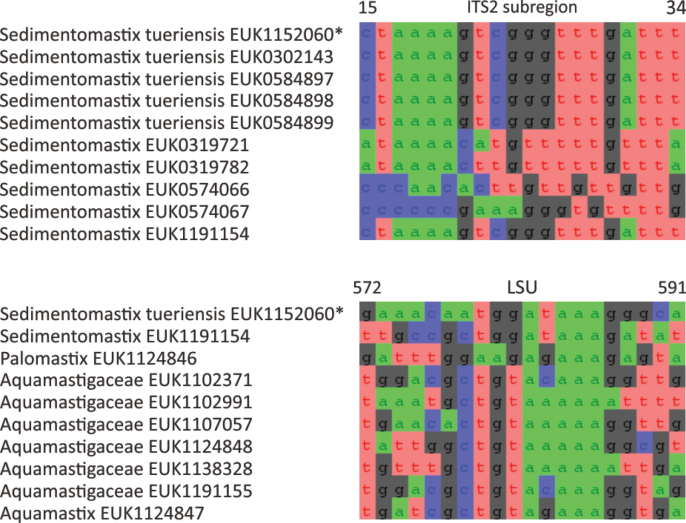
Diagnostic nucleotide sequences of *Sedimentomastix tueriensis* relative to the closest related species in ITS2 and LSU. Numbers indicate positions in the legitype (marked with an asterisk).

Separation from other species of *Sedimentomastix* based on ITS2 (positions 15–34 ctaaaagtcgggtttgattt; one mismatch allowed) and LSU D2 (positions 572–591 gaaacaatggataaagggca; one mismatch allowed) as indicated in Fig. 26. Intraspecific variation up to 1.7% in ITS2. Interspecific distance > 15% in ITS2.

##### Type.

Wastewater sample TUE032723 (**holotype**); eDNA sequence EUK1152060 = OZ253798 (**legitype**); eDNA sample TUE132723 (**nucleotype**), Türi, Estonia, 58.8156°N, 25.4067°E.

##### Description.

Other sequences: EUK0302143 (*Populus tremula* forest soil in Soinaste, Estonia, 58.3408°N, 26.6864°E); EUK0584897 (FunAqua stream water sample in Kangilleq, Greenland, 60.8571, –46.4233); EUK0584898 (FunAqua river water sample W0597w in Aveleda, Portugal, 41.8919, –6.6972); and EUK0584899 (FunAqua lake sediment sample W0307s in Goldwin, ND, USA, 47.0996, –99.0916).

##### Etymology.

*Sedimentomastix* refers to *sedimentum* (the Latin term for sediment) and *Neocallimastix*; the epithet refers to *Türi* (Estonian), the type locality.

##### Notes.

Found in water, sediment, and soil samples in Europe and North America (n = 5 records). GlobalFungi includes nine additional records, mainly from wetland soils in Europe, Asia, and North America.

#### Mucoromycota

Taxon classificationFungiFungiFungi

 Tedersoo, Sanchez-Ramirez, Kõljalg, Bahram, M. Döring, Schigel, T.W. May, M. Ryberg & Abarenkov, Fungal Diversity 90: 151 (2018)

9AAB646E-F816-588A-9ADF-4674F62EE7BE

554016

##### Type phylum.

Mucoromycota Doweld.

##### Description.

As in Tedersoo et al. (2018)

##### Notes.

Currently harbors Calcarisporiellomycota, Glomeromycota, Mucoromycota, Mortierellomycota, and Curlevskiomycota (phyl. nov.).

#### Calcarisporiellomycota

Taxon classificationFungiFungiMucoromycota

Tedersoo, Sanchez-Ramirez, Kõljalg, Bahram, M. Döring, Schigel, T.W. May, M. Ryberg & Abarenkov, Fungal Diversity 90: 152 (2018)

18D6DA70-537B-5ABD-B849-0BB0CEF094F9

554019

##### Type class.

Calcarisporiellomycetes Tedersoo, Sanchez-Ramirez, Kõljalg, Bahram, M. Döring, Schigel, T.W. May, M. Ryberg & Abarenkov.

##### Description.

As in Tedersoo et al. (2018)

#### Calcarisporiellomycetes

Taxon classificationFungiMucoromycotaCalcarisporiellomycota

Tedersoo, Sanchez-Ramirez, Kõljalg, Bahram, M. Döring, Schigel, T.W. May, M. Ryberg & Abarenkov, Fungal Diversity 90: 152 (2018)

6422C218-6276-52E2-A2B7-7C965D54FD6A

554020

##### Type order.

Calcarisporiellales Tedersoo, Sanchez-Ramirez, Kõljalg, Bahram, M. Döring, Schigel, T.W. May, M. Ryberg & Abarenkov.

##### Description.

As in Tedersoo et al. (2018).

##### Notes.

Recognized based on eDNA sequences only. Currently includes Calcarisporiellales and Terrincolales (ord. nov.).

#### Terrincolales

Taxon classificationFungiCalcarisporiellomycotaCalcarisporiellomycetes

Tedersoo & Esmaeilzadeh-Salestani ord. nov.

BCA3DB63-0FC9-5F7D-9479-CA4F0F8FB536

859027

##### Type family.

Terrincolaceae Tedersoo & Esmaeilzadeh-Salestani.

##### Diagnosis.

Distinguishable from other fungi based on diagnostic nucleotide signature in 5.8S (positions 6–26 in type species and *S. cerevisiae* ttcaacaatggatccctcg; no mismatch allowed), LSU D1 (positions 4–23 in type species and *S. cerevisiae* tcctcaaatcaagcaagagt; no mismatch allowed), LSU D2 (positions 255–264 in type species and 244–253 in *S. cerevisiae* ttggtagtgg; one mismatch allowed), and SSU V3 (positions 647–651 ggcttg in *S. cerevisiae*; no mismatch allowed). Forms a monophyletic, least inclusive clade in Calcarisporiellomycetes, covering sequences MW791967, EUK1138132, EUK1123677, EUK0332618, EUK1123675, EUK1604147, EUK1604155, and EUK1123676 (Fig. 1).

##### Notes.

Recognized based on eDNA sequences only. Encoded as clade GS94 in EUKARYOME v1.9. Currently includes Terrincolaceae (fam. nov.) and a potentially family-level group represented by sequences EUK1604147 (tundra soil in AK, USA) and EUK1604155 (forest soil in LO, USA). Terrincolales comprises potentially 50–70 species. Detected exclusively in soil (100% out of the 249 records) in tundra to hot tropical biomes across all continents, excluding Antarctica.

#### Terrincolaceae

Taxon classificationFungiCalcarisporiellomycetesTerrincolales

Tedersoo & Esmaeilzadeh-Salestani fam. nov.

E4BE1CA3-D6EC-5C59-8491-277CCC6A32FF

859028

##### Type genus.

*Terrincola* Tedersoo & Esmaeilzadeh-Salestani.

##### Diagnosis.

Distinguishable from other members of Calcarisporiellomycota based on diagnostic nucleotide signature in ITS2 (positions 285–292 aaaatrtt; one mismatch allowed). Forms a monophyletic, least inclusive clade in Terrincolales, covering sequences MW791967, EUK1138132, EUK1123677, EUK0332618, EUK1123675, and EUK1123676 (Fig. 1).

##### Notes.

Recognized based on eDNA sequences only. Includes *Terrincola* (gen. nov.) and several potentially genus-level groups represented by sequences EUK1123677 (forest soil in Estonia), EUK0332618 (forest soil in Colombia), EUK1123675 (wasteland soil in Estonia), and EUK1123676 (garden soil in Estonia).

#### Terrincola

Taxon classificationFungiTerrincolalesTerrincolaceae

Tedersoo & Esmaeilzadeh-Salestani gen. nov.

92F02C2B-343D-5015-8C61-02449E3D9EF9

859029

##### Type species.

*Terrincola waldropii* Tedersoo & Esmaeilzadeh-Salestani.

##### Diagnosis.

**Figure 27.**
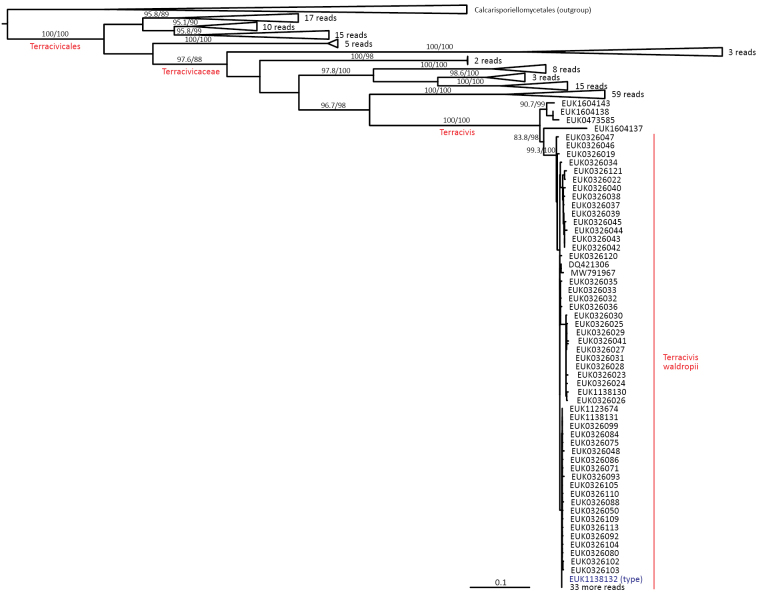
Maximum Likelihood SSU-ITS-LSU phylogram indicating the position of *Terrincola waldropii* within Terrincolales with ultra-rapid bootstrap values indicated (for higher-level classifications mainly). Other genus-level groups are collapsed. Calcarisporiellomycetes spp. were used as an outgroup.

Distinguishable from other fungi based on diagnostic nucleotide signature in ITS2 (positions 23–32 ggccgtacgg; one mismatch allowed). Forms a monophyletic, least inclusive clade in Terrincolaceae, covering sequences MW791967, EUK0473585, and EUK1138132 (Figs 1, 27).

##### Notes.

Recognized based on eDNA sequences only. Comprises potentially four species represented by sequences EUK0473585 (shrubland soil in Uyghur Autonomous Region, China), EUK1604143 (forest soil in VT, USA), and EUK1604137 (forest soil in South Africa).

#### Terrincola waldropii

Taxon classificationFungiTerrincolalesTerrincolaceae

Tedersoo & Esmaeilzadeh-Salestani sp. nov.

75FB2191-61D0-507C-8807-A426187E95D5

859031

##### Diagnosis.

**Figure 28.**
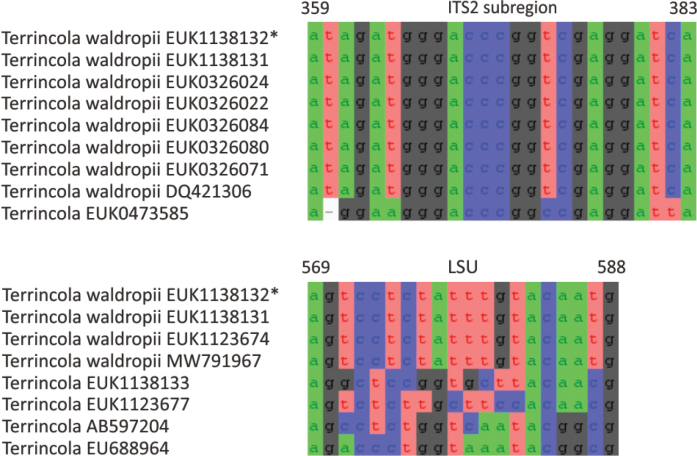
Diagnostic nucleotide sequences of *Terrincola waldropii* relative to the closest related species in ITS2 and LSU. Numbers indicate positions in the legitype (marked with an asterisk).

Separation from other species of *Terrincola* based on diagnostic nucleotide signatures in ITS2 (positions 359–383 atagatgggacccggtcgaggatca; one mismatch allowed) and LSU D2 (positions 569–588 agtcctctatttgtacaatg; one mismatch allowed) as indicated in Fig. 28. Intraspecific variation up to 1.2% in ITS2 and up to 0.5% in LSU sequences. Interspecific distance at least 4.9% in ITS2 and 3.9% in LSU.

##### Type.

Vouchered soil sample TUE000813 (**holotype**); DNA sequence EUK1138132 = OZ253800 (**legitype**); eDNA sample TUE100813 (**nucleotype**); GSMc plot S281, *Quercus robur* alley in Tartu, Estonia, 58.379°N, 26.706°E.

##### Description.

Other sequences: EUK1138131 (GSMc plot G5295, *Pinus mugo* plantation soil in Märja, Estonia, 58.3592°N, 26.6443°E); EUK0326024 (GSMc plot S1087, tundra soil in Zackenberg, Greenland, 74.4682, –20.6142); EUK0326022 (GSMc plot G5038, tropical dry forest soil in West End, British Virgin Islands, 18.3907, –64.7073); EUK0326084 (GSMc plot S639, subtropical forest soil in Platbos, South Africa, –34.5676, 19.4461); EUK0326080 (GSMc plot S618, montane desert soil in Tanglang La, India, 33.5051°N, 77.7655°E); EUK0326071 (GSMc plot G5769, temperate grassland soil in Rõõmu, Estonia, 58.3877°N, 26.7770°E); and DQ421306 (temperate grassland soil in Cedar Creek, MN, USA, 45.40, –93.19).

##### Etymology.

*Terra* and *incola* (Latin) refer to the soil habitat, and *Waldrop* (English) refers to the last name of Mark P. Waldrop, who was the first to collect material of this species and order (DQ421306; Waldrop et al. 2006).

##### Notes.

All 109 records originate from soil. This is supported by GlobalFungi data, where > 98% of 732 records are from soil and 1% from roots. Found in all biomes and continents, excluding Antarctica.

#### Curlevskiomycota

Taxon classificationFungiFungiMucoromycota

Tedersoo phyl. nov.

8AA271D4-AAE9-52EF-B2B4-E3DDF66B55D9

859032

##### Type class.

Curlevskiomycetes Tedersoo.

##### Diagnosis.

Distinguishable from other fungi based on diagnostic nucleotide signatures in the LSU 5’ end (positions 52–73 in the type species and *S. cerevisiae* ccgaggaaaagaaactaacaag or tggaggaaaagaaaaaaacatt; no mismatch allowed). Forms a monophyletic, least inclusive clade in fungi, covering sequences EUK1124408, EUK1103576, MG664460, EUK1700038, EUK1700047, EUK1124407, EUK1631674, EUK1103826, EUK1103868, EUK1700102, EUK1603989, EUK1603990, KF849654, EUK1103703, EUK1603986, EUK1602443, EUK1603988, EUK1124409, and EUK1630897 (Fig. 1).

##### Notes.

Recognized based on eDNA sequences only. Encoded as clade GS50 in EUKARYOME v1.9. Currently harbors Curlevskiomycetes (class. nov.) and potentially several class-level groups represented by sequences EUK1124408 (wetland soil in Estonia), EUK1103576 (forest soil in Puerto Rico), MG664460 (cropland soil in China), EUK1700038 (woodland soil in NT, Australia), EUK1700047 (desert soil in Saudi Arabia), EUK1124407 (wasteland soil in Estonia), and EUK1631674 (forest soil in Estonia). Comprises potentially 100–120 species. Detected in soil (97.5% out of the 163 records) and plant roots (1.8%) in boreal forest to hot tropical biomes across all continents except Antarctica.

#### Curlevskiomycetes

Taxon classificationFungiMucoromycotaCurlevskiomycota

Tedersoo class. nov.

5FB81302-0D3D-5E6B-B94E-2B2D09832961

859033

##### Type order.

Curlevskiales Tedersoo.

##### Diagnosis.

Distinguishable from other fungi based on diagnostic nucleotide signatures in LSU D2 (positions 549–559 in type species and 494–504 in *S. cerevisiae* tcagcgtcagc; one mismatch allowed). Forms a monophyletic, least inclusive clade in Curlevskiomycota, covering sequences EUK1103826, EUK1103868, EUK1700102, EUK1603989, EUK1603990, KF849654, EUK1103703, EUK1603986, EUK1602443, EUK1603988, EUK1124409, and EUK1630897 (Fig. 1).

##### Notes.

Recognized based on eDNA sequences only. Currently harbors Curlevskiales (ord. nov.) and potentially 1–2 order-level groups represented by sequences EUK1103826 (forest soil in Puerto Rico) and EUK1700078 (forest soil in Gabon).

#### Curlevskiales

Taxon classificationFungiCurlevskiomycotaCurlevskiomycetes

Tedersoo ord. nov.

639BAD86-7874-5BE9-8628-B740BFE547C6

859034

##### Type family.

Curlevskiaceae Tedersoo.

##### Diagnosis.

Distinguishable from other fungi based on diagnostic nucleotide signatures in ITS2 (positions 88–97 tcgcgaatcc or tcgcaaaacg; one mismatch allowed) and LSU D2 (positions 541–550 in type species and 486–495 in *S. cerevisiae* cacgcaggtc; one mismatch allowed). Forms a monophyletic, least inclusive clade in Curlevskiomycetes, covering sequences EUK1700102, EUK1603989, EUK1603990, KF849654, EUK1103703, EUK1603986, EUK1602443, EUK1603988, EUK1124409, and EUK1630897 (Fig. 1).

##### Notes.

Recognized based on eDNA sequences only. Currently includes Curlevskiaceae (fam. nov.).

#### Curlevskiaceae

Taxon classificationFungiCurlevskiomycetesCurlevskiales

Tedersoo fam. nov.

266F3791-24AF-5BDF-B449-CC127074AB0E

859035

##### Type genus.

*Curlevskia* Tedersoo.

##### Diagnosis.

Distinguishable from other fungi based on diagnostic nucleotide signatures in ITS2 (positions 88–97 tcgcgaatcc or tcgcaaaacg; one mismatch allowed) and LSU D2 (positions 541–550 in type species and 486–495 in *S. cerevisiae* cacgcaggtc; one mismatch allowed). Forms a monophyletic, least inclusive clade in Curlevskiales, covering sequences EUK1700102, EUK1603989, EUK1603990, KF849654, EUK1103703, EUK1603986, EUK1602443, EUK1603988, EUK1124409, and EUK1630897 (Fig. 1).

##### Notes.

Recognized based on eDNA sequences only. Includes *Curlevskia* and another potentially genus-level group represented by sequences EUK1700102 (forest soil in VIC, Australia).

#### Curlevskia

Taxon classificationFungiCurlevskialesCurlevskiaceae

Tedersoo gen. nov.

1CE152A9-2496-5A72-A72A-43EC0B83B291

859036

##### Type species.

*Curlevskia holarctica* Tedersoo.

##### Diagnosis.

**Figure 29.**
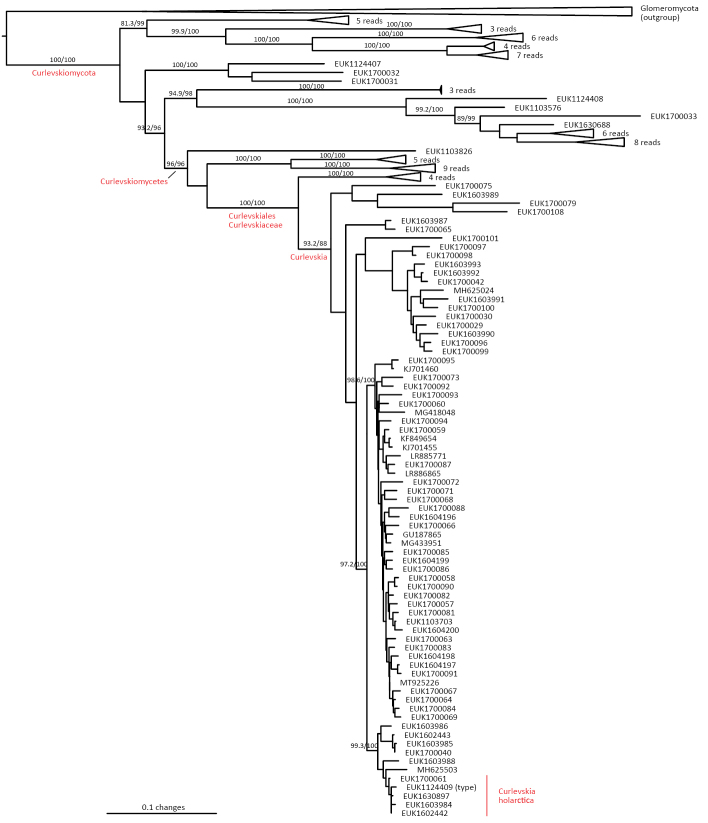
Maximum Likelihood SSU-ITS-LSU phylogram indicating the position of *Curlevskia holarctica* within Curlevskiomycota, with ultra-rapid bootstrap values indicated (for higher-level classifications mainly). Other genus-level groups are collapsed. Glomeromycota spp. were used as an outgroup.

Distinguishable from other fungi based on diagnostic nucleotide signatures in ITS2 (positions 88–97 in type species tcgcgaatcc; one mismatch allowed) and LSU D2 (positions 565–574 in type species and 509–518 in *S. cerevisiae* atcgcgggaa; one mismatch allowed). Forms a monophyletic, least inclusive clade in Curlevskiaceae, covering sequences EUK1603989, EUK1603990, KF849654, EUK1103703, EUK1603986, EUK1602443, EUK1603988, EUK1124409, and EUK1630897 (Figs 1, 29).

##### Notes.

Recognized based on eDNA sequences only. Comprises 40–60 species represented by sequences EUK1603985 (wasteland soil in Estonia), EUK1603986 (cropland soil in Estonia), EUK1603987 (grassland soil in Estonia), EUK1603988 (wasteland soil in Estonia), KJ701460, KJ701455, and KF849654 (all from plant roots in China), GU187865 (woodland soil in VIC, Australia), and EUK1103703 (forest soil in Puerto Rico).

#### Curlevskia holarctica

Taxon classificationFungiCurlevskialesCurlevskiaceae

Tedersoo sp. nov.

0FEC9C80-9B77-5CE8-88DB-4FA58FBB5613

859037

##### Diagnosis.

**Figure 30.**
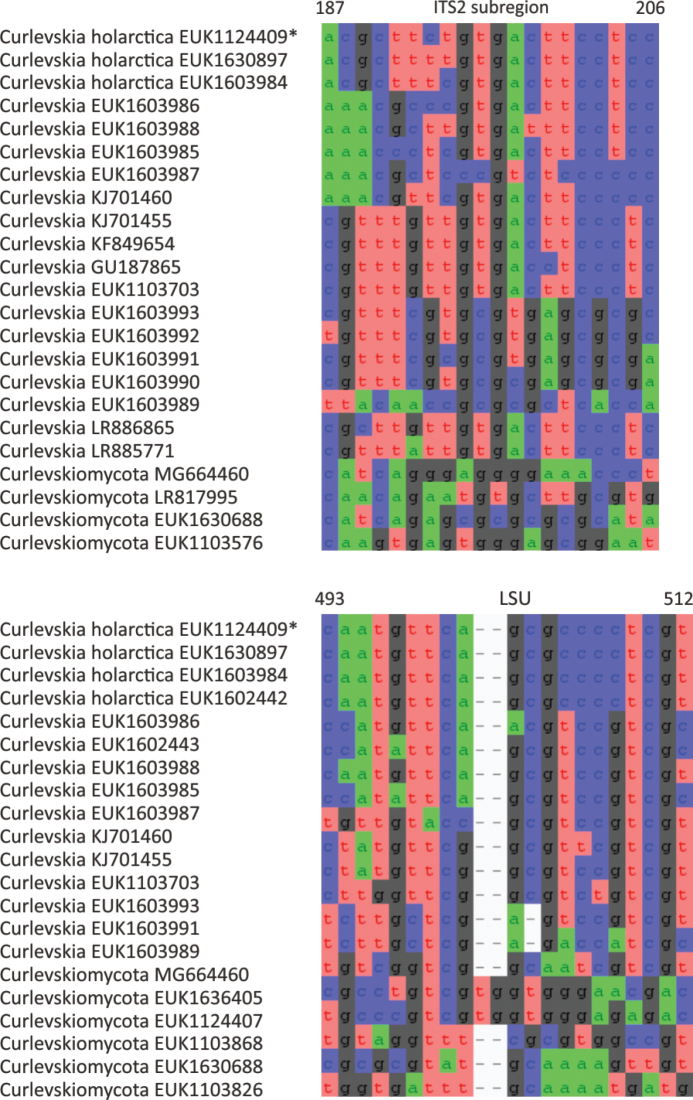
Diagnostic nucleotide sequences of *Curlevskia holarctica* relative to the closest related species in ITS2 and LSU. Numbers indicate positions in the legitype (marked with an asterisk).

Separation from other species of *Curlevskia* based on ITS2 (positions 187–206 acgcttytgtgacttcctcc; two mismatches allowed) and LSU D2 (positions 493–512 caatgttcagcgcccctcgt; no mismatch allowed) as indicated in Fig. 30. Intraspecific variation up to 2.1% in ITS2 and 1.3% in LSU. Interspecific distance at least 4.4% in ITS2 and 2.8% in LSU.

##### Type.

Vouchered soil sample TUE002212 (**holotype**); eDNA sequence EUK1124409 = OZ253801 (**legitype**); eDNA sample TUE102212 (**nucleotype**); GSMc plot G5235, *Larix* sp. plantation in Rõõmu, Estonia, 58.3835°N, 26.7742°E.

##### Description.

Other sequences: EUK1630897 (GSMc plot G4803, *Ulmus-Alnus* forest soil in Meegaste, Estonia, 58.0563°N, 26.3355°E); EUK1603984 (GSMc plot G5821, gravel quarry soil in Siimusti, Estonia, 58.7306°N, 26.3198°E); EUK1602442 (GSMc plot G4128, *Quercus robur*woodland soil in Ööriku, Estonia, 58.5831°N, 22.9322°E); and EUK1700061 (GSMc plot IH.ME05, *Abies* forest soil in Mestia, Georgia, 43.0209°N, 42.7325°E).

##### Etymology.

*Curlevski* refers to Nathalie J. A. Curlevski, who was the first to collect material of this genus (GU187865; Curlevski et al. 2014), and *holarctica* refers to its distribution.

##### Notes.

Found in soil in four contrasting sites in Estonia and once in Georgia. The 11 additional records in GlobalFungi point to a broader distribution in Eurasian and North American soils.

#### Mortierellomycota

Taxon classificationFungiFungiMucoromycota

Tedersoo, Sanchez-Ramirez, Kõljalg, Bahram, M. Döring, Schigel, T.W. May, M. Ryberg & Abarenkov, Fungal Diversity 90: 152 (2018)

442E8421-2AC5-59A5-BD6D-A20D463FBDC3

554018

##### Type class.

Mortierellomycetes Doweld.

##### Description.

As in Tedersoo et al. (2018).

##### Notes.

Currently harbors Mortierellomycetes, Maerjamycetes (class. nov.), and Ruderaliomycetes (class. nov.).

#### Mortierellomycetes

Taxon classificationFungiMucoromycotaMortierellomycota

Doweld Index Fungorum 46: 1 (2014)

D5EDF9C6-663F-522A-9FBC-3CDDFCEFCC7A

550332

##### Type order.

Mortierellales Caval.-Sm.

##### Description.

As in Doweld (2014).

##### Notes.

Currently harbors Mortierellales and Mycosocceriales (ord. nov.).

#### Mycosocceriales

Taxon classificationFungiMortierellomycotaMortierellomycetes

Tedersoo, Bahram & Esmaeilzadeh-Salestani ord. nov.

80E2C6E7-22AC-5D49-8845-C73A33A25A0E

859039

##### Type family.

Mycosocceriaceae Tedersoo, Bahram & Esmaeilzadeh-Salestani.

##### Diagnosis.

Distinguishable from other species of Mortierellomycota based on diagnostic nucleotide signature in SSU V9 (positions 1654–1663 in *S. cerevisiae* gattgaacgg; no mismatch allowed) and from all fungi in LSU D2 (positions 573–92 in the type species and 521–540 in *S. cerevisiae* aagttggaggaatgtggctc; two mismatches allowed). Forms a monophyletic, least inclusive clade in Mortierellomycetes, covering sequences EUK0531595, EUK1102426, EUK1202279, EUK0531631, EUK1008618, and EUK1124462 (Fig. 1).

##### Notes.

Recognized based on eDNA sequences only. Encoded as clade GS61 in EUKARYOME v1.9. Currently includes Mycosocceriaceae (fam. nov.). Comprises potentially 20–30 species. Detected in soil (93.2% out of the 74 records) and sediments (6.8%) in cold temperate to hot tropical biomes across all continents except Antarctica.

#### Mycosocceriaceae

Taxon classificationFungiMortierellomycetesMycosocceriales

Tedersoo, Bahram & Esmaeilzadeh-Salestani fam. nov.

0082E1A2-F121-5F05-BEC5-A7528320CB27

859040

##### Type genus.

*Mycosocceria* Tedersoo, Bahram & Esmaeilzadeh-Salestani.

##### Diagnosis.

Distinguishable from other species of Mortierellomycetes based on diagnostic nucleotide signatures in SSU V9 (positions 1654–1663 in *S. cerevisiae* gattgaacgg; no mismatch allowed), 5.8S (positions 90–99 in type species and *S. cerevisiae* tcatcaaatc; no mismatch allowed), and LSU D2 (positions 573–592 in type species and 521–540 in *S. cerevisiae* aagttggaggaatgtggctc; two mismatches allowed). Forms a monophyletic, least inclusive clade in Mycosocceriales, covering sequences EUK0531595, EUK1102426, EUK1202279, EUK0531631, EUK1008618, and EUK1124462 (Fig. 1).

##### Notes.

Recognized based on eDNA sequences only. Currently includes *Mycosocceria* (gen. nov.) and other potentially genus-level groups represented by sequences EUK1102426 (forest soil in Puerto Rico), EUK0531595 (orchard soil in Estonia), and EUK1202279 (forest soil in Italy).

#### Mycosocceria

Taxon classificationFungiMycosoccerialesMycosocceriaceae

Tedersoo, Bahram & Esmaeilzadeh-Salestani gen. nov.

79531C1D-48A1-5E80-BC77-64DCEEF403F5

859041

##### Type species.

*Mycosocceria estonica* Tedersoo, Bahram & Esmaeilzadeh-Salestani.

##### Diagnosis.

**Figure 31.**
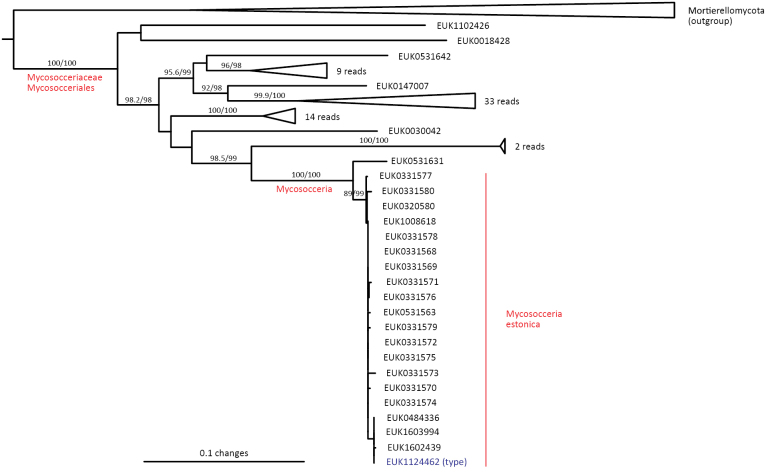
Maximum Likelihood SSU-ITS-LSU phylogram indicating the position of *Mycosocceria estonica* within Mycosocceriales, with ultra-rapid bootstrap values indicated (for higher-level classifications mainly). Other genus-level groups are collapsed. Mortierellomycota spp. were used as an outgroup.

Distinguishable from other species of fungi based on diagnostic nucleotide signatures in 5.8S (positions 120–129 in type species and *S. cerevisiae* cccggtaggc). Forms a monophyletic, least inclusive clade in Mycosocceriaceae, covering sequences EUK1008618, EUK0531631, and EUK1124462 (Figs 1, 31).

##### Notes.

Recognized based on eDNA sequences only. Includes *M. estonica* and another species represented by sequence EUK0531631 (savanna soil in Uganda).

#### Mycosocceria estonica

Taxon classificationFungiMycosoccerialesMycosocceriaceae

Tedersoo, Bahram & Esmaeilzadeh-Salestani sp. nov.

F1A3F556-0B4B-522C-B7E6-A5F769B5FBDE

859042

##### Diagnosis.

**Figure 32.**
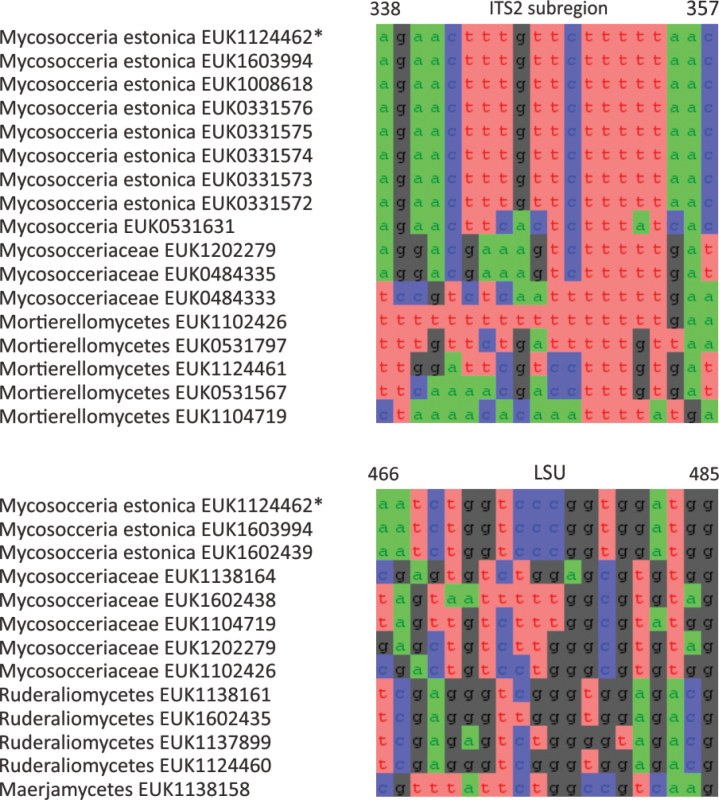
Diagnostic nucleotide sequences of *Mycosocceria estonica* relative to the closest related species in ITS2 and LSU. Numbers indicate positions in the legitype (marked with an asterisk).

Separation from other species of *Mycosocceria* based on ITS2 (positions 338–357 agaactttgttctttttaac; one mismatch allowed) and LSU D2 (positions 466–485 aatctggtcccggtggatgg; one mismatch allowed) as indicated in Fig. 32. Intraspecific variation up to 2.3% in ITS2 and 0.2% in LSU. Interspecific distance at least 10.2% in ITS2 and 10.3% in LSU.

##### Type.

Vouchered soil sample TUE028391 (**holotype**); eDNA sequence EUK1124462 = OZ253802 (**legitype**); eDNA sample TUE128391 (**nucleotype**); GSMc plot G5802, football field in Veeriku, Estonia, 58.3745°N, 26.6855°E.

##### Description.

Other sequences: EUK1603994 (GSMc plot G5816, *Trifolium pratense* cropland soil in Hermani, Estonia, 58.8071°N, 25.7564°E); EUK1008618 (GSMc plot G4599, *Ulmus laevis* forest soil in Liutsepa, Estonia, 58.0791°N, 26.0094°E); EUK0331576 (GSMc plot G5820, *Acer-Fraxinus-Ulmus*woodland soil in Pajusi, Estonia, 58.7052°N, 25.9389°E); EUK0331575 (GSMc plot G5422, *Pinus strobus*woodland soil in Tartu, Estonia, 58.3909°N, 26.6973°E); EUK0331574 (GSMc plot G5765y, grassland soil in Rebaste, Estonia, 58.41°N, 25.93°E); EUK0331573 (urban park soil in Slovenia); and EUK0331572 (GSMc plot S950, forest tundra soil in Mt. Mayak, Altai kray, Russian Federation, 51.0474°N, 82.9718°E).

##### Etymology.

*Soccer* (English) refers to the football field where the type specimen was collected; and *estonica* (Latin) refers to Estonia, where this species’ type and most additional materials originate.

##### Notes.

All but one of the 40 records and all five GlobalFungi records are derived from soil. Found mainly in North Eurasia, with occasional records elsewhere.

#### Maerjamycetes

Taxon classificationFungiMucoromycotaMortierellomycota

Tedersoo & Esmaeilzadeh-Salestani class. nov.

D8D9CB68-17E6-564F-B7D0-E91E00A1A572

859043

##### Type order.

Maerjamycetales Tedersoo & Esmaeilzadeh-Salestani.

##### Diagnosis.

Distinguishable from other fungi based on diagnostic nucleotide signatures in SSU V9 (positions 1684–1690 in *S. cerevisiae* gatgcat; no mismatch allowed) and LSU D1 (positions 114–122 in type species and 115–123 in *S. cerevisiae* cactttctg; no mismatch allowed). Forms a monophyletic, least inclusive clade in Mortierellomycota, covering sequences EUK1200032, EUK1217336, EUK1009005, EUK0484311, EUK0484301, and EUK1138158 (Fig. 1).

##### Notes.

Recognized based on eDNA sequences only. Encoded as clade GS48 in EUKARYOME v1.9. Currently harbors Maerjamycetales (ord. nov.). Comprises around five species. Detected in soil (90.6% out of the 276 records), sediments (7.6%), water (1.1%), and old paper (0.7%) in high arctic to hot tropical biomes across all continents except Antarctica.

#### Maerjamycetales

Taxon classificationFungiMortierellomycotaMaerjamycetes

Tedersoo & Esmaeilzadeh-Salestani ord. nov.

9773BE4E-9564-5CC9-8079-EE6582AC5F4B

859044

##### Type family.

Maerjamycetaceae Tedersoo & Esmaeilzadeh-Salestani.

##### Diagnosis.

Distinguishable from other fungi based on diagnostic nucleotide signatures in SSU V9 (positions 1684–1690 in *S. cerevisiae* gatgcat; no mismatch allowed) and LSU D1 (positions 114–122 in type species and 115–123 in *S. cerevisiae* cactttctg; no mismatch allowed). Forms a monophyletic, least inclusive clade in Maerjamycetes, covering sequences EUK1200032, EUK1217336, EUK1009005, EUK0484311, EUK0484301, and EUK1138158 (Fig. 1).

##### Notes.

Recognized based on eDNA sequences only. Currently includes Maerjamycetaceae (fam. nov.) and another potentially family-level group represented by sequences EUK1200032, EUK1217336, and EUK1009005 (all forest soil in Estonia).

#### Maerjamycetaceae

Taxon classificationFungiMaerjamycetesMaerjamycetales

Tedersoo & Esmaeilzadeh-Salestani fam. nov.

2432328F-B50A-5622-8BD7-D02B31126714

859045

##### Type genus.

*Maerjamyces* Tedersoo & Esmaeilzadeh-Salestani.

##### Diagnosis.

Distinguishable from other fungi based on diagnostic nucleotide signatures in 5.8S (positions 77–86 in type species and 78–87 in *S. cerevisiae* agagtacgtg; one mismatch allowed). Forms a monophyletic, least inclusive clade in Maerjamycetales, covering sequences EUK1138158, EUK0484301, and EUK0484311 (Fig. 1).

##### Notes.

Recognized based on eDNA sequences only. Maerjamycetaceae is currently monogeneric.

#### Maerjamyces

Taxon classificationFungiMaerjamycetalesMaerjamycetaceae

Tedersoo & Esmaeilzadeh-Salestani gen. nov.

2A849F7A-1A0A-5727-9854-362E7BD51733

859046

##### Type species.

*Maerjamyces jumpponenii* Tedersoo & Esmaeilzadeh-Salestani.

##### Diagnosis.

**Figure 33.**
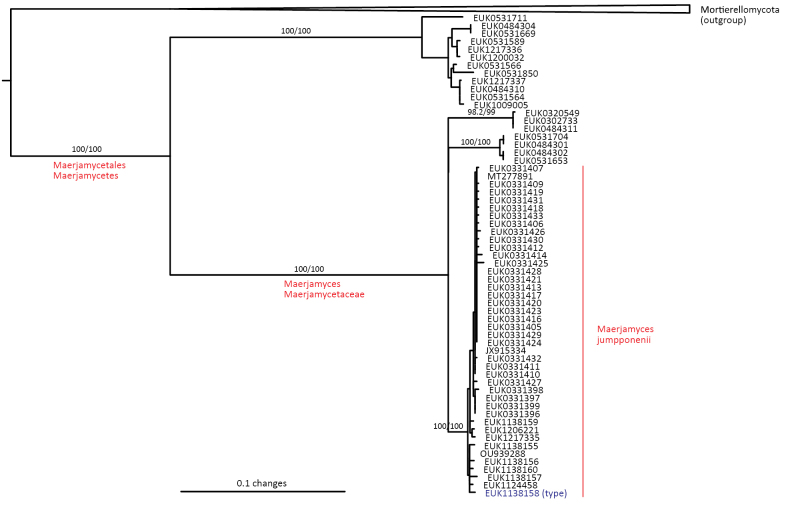
Maximum Likelihood SSU-ITS-LSU phylogram indicating the position of *Maerjamyces jumpponenii* within Maerjamycetes, with ultra-rapid bootstrap values indicated. Other genus-level groups are collapsed. Mortierellomycota spp. were used as an outgroup.

Distinguishable from other fungi based on diagnostic nucleotide signatures in 5.8S (positions 77–86 in type species and 78–87 in *S. cerevisiae* agagtacgtg; one mismatch allowed). Forms a monophyletic, least inclusive clade in Maerjamycetaceae, covering sequences EUK1138158, EUK0484301, and EUK0484311 (Figs 1, 33).

##### Notes.

Recognized based on eDNA sequences only. Comprises potentially three species, represented by sequences EUK0484301 (tundra soil in Svalbard) and EUK0484311 (forest soil in OR, USA).

#### Maerjamyces jumpponenii

Taxon classificationFungiMaerjamycetalesMaerjamycetaceae

Tedersoo & Esmaeilzadeh-Salestani sp. nov.

235650E0-26F0-5220-9505-EBB267468D4A

859047

##### Diagnosis.

**Figure 34.**
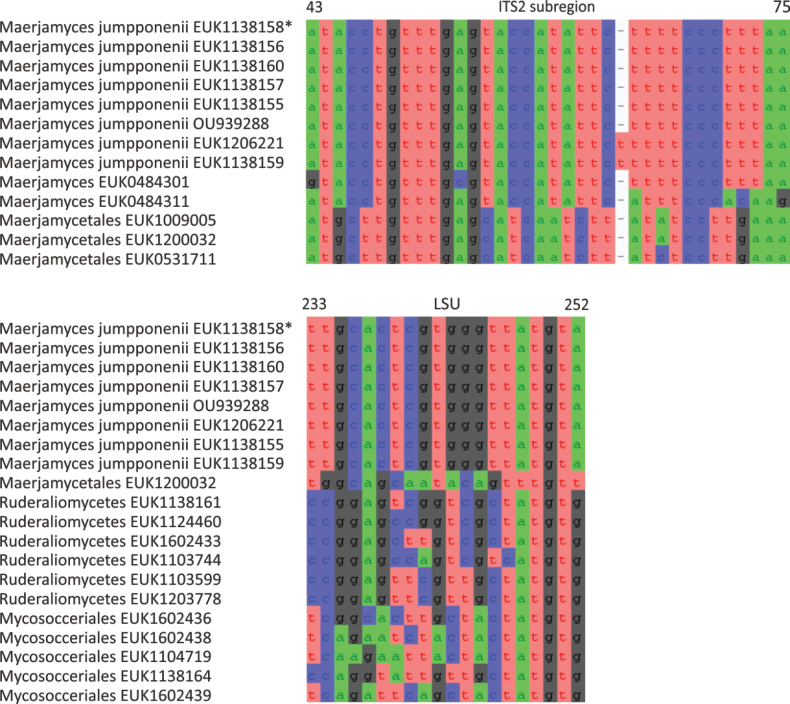
Diagnostic nucleotide sequences of *Maerjamyces jumpponenii* relative to the closest related species in ITS2 and LSU. Numbers indicate positions in the legitype (marked with an asterisk).

Separation from other species of *Maerjamyces* based on ITS2 (positions 43–75 atacctgtttgagtaccatattcttttcccttt; one mismatch allowed) and LSU D1 (positions 233–252 ttgcactcgtgggttatgta; one mismatch allowed) as indicated in Fig. 34. Intraspecific variation up to 5.4% in ITS2 and up to 1.6% in LSU. Closest species differ by at least 7.4% in ITS2 and 5.0% in LSU.

##### Type.

Vouchered soil sample TUE002272 (**holotype**); eDNA sequence EUK1138158 = OZ253803 (**legitype**); eDNA sample TUE102272 **(nucleotype**); GSMc plot G5295, *Pinus mugo* plantation soil in Märja, Estonia, 58.3592°N, 26.6443°E.

##### Description.

Other sequences: EUK1138156 (GSMc plot G5235, *Larix decidua* plantation soil in Rõõmu, Estonia, 58.3835°N, 26.7742°E); EUK1138160 (GSMc plot G5803, urban park soil in Toomemägi, Estonia, 58.3786°N, 26.7185°E); EUK1138157 (GSMc plot G5283, *Quercus robur* plantation in Rahinge, Estonia, 58.3845°N, 26.5943°E); MT277862 (book paper in Turin, Italy); OU939288 (Kungsängen, Sweden, 59.837°N, 17.661°E); (GSMc plot G4800, *Ulmus laevis* forest soil in Tuhkja, Estonia, 58.4159°N, 25.2326°E); and EUK1138159 (urban soil in Tartu, Estonia, 58.3913°N, 26.6965°E).

##### Etymology.

*Maerjamyces* refers to the type locality in Märja (Estonian), and *mykos* (Greek) stands for a fungus; *Jumpponen* (Finnish) refers to Ari Jumpponen, who was the first to collect material of this species (FJ780627; Jumpponen et al. 2010).

##### Notes.

Found mainly in soil (90.4%) but also from sediments, water, and paper samples (260 total records). Occurs on all continents, but > 95% of records originate from the temperate and Mediterranean biomes of the Northern Hemisphere. Out of 244 GlobalFungi records, 98.4% are derived from soil.

#### Ruderaliomycetes

Taxon classificationFungiMucoromycotaMortierellomycota

Tedersoo class. nov.

D98BD966-18B4-5B31-8C9F-3AC3F9B47D2D

859048

##### Type order.

Ruderaliales Tedersoo.

##### Diagnosis.

Distinguishable from other fungi based on a diagnostic nucleotide signature in LSU D2 (positions 631–640 in the type species and 564–573 in *S. cerevisiae* acggatacgg; one mismatch allowed). Forms a monophyletic, least inclusive clade in Mortierellomycota, covering sequences EUK1138161, EUK1137899, EUK1124460, EUK0531800, EUK1103744, EUK1103025, EUK1103555, EUK1103599, EUK1203462, and EUK1700231 (Fig. 1).

##### Notes.

Recognized based on eDNA sequences only. Encoded as clade GS49 in EUKARYOME v1.9. Currently harbors the single order Ruderaliales (ord. nov.). Comprises potentially 40–50 species. Detected in soil (98.5% out of the 329 records) and sediments (1.5%) in cold temperate to hot tropical biomes across all continents except Antarctica.

#### Ruderaliales

Taxon classificationFungiMortierellomycotaRuderaliomycetes

Tedersoo ord. nov.

40AEC11B-E10C-559B-9B01-5AD535B19F17

859049

##### Type family.

Ruderaliaceae Tedersoo.

##### Diagnosis.

Distinguishable from other fungi based on a diagnostic nucleotide signature in LSU D2 (positions 631–640 in the type species and 564–573 in *S. cerevisiae* acggatacgg; one mismatch allowed). Forms a monophyletic, least inclusive clade in Ruderaliomycetes, covering sequences EUK1138161, EUK1137899, EUK0531800, EUK1124460, EUK1103744, EUK1103025, EUK1103555, EUK1103599 and EUK1203462 (Fig. 1).

##### Notes.

Recognized based on eDNA sequences only. Currently includes Ruderaliaceae and another potentially family-level group represented by sequences EUK1103744, EUK1103555, and EUK1103599 (all forest soil in Puerto Rico) and EUK1203462 (lake sediment in Croatia).

#### Ruderaliaceae

Taxon classificationFungiRuderaliomycetesRuderaliales

Tedersoo fam. nov.

9DB42ADB-8BE2-52F2-9DDF-699CF5AD0277

859050

##### Type genus.

*Ruderalia* Tedersoo.

##### Diagnosis.

Distinguishable from other fungi based on a diagnostic nucleotide signature in ITS2 (positions 209–222 in type species aacgatagtgaagt; two mismatches allowed). Forms a monophyletic, least inclusive clade in Ruderaliales, covering sequences EUK1138161, EUK1137899, EUK0531800, and EUK1124460 (Fig. 1).

##### Notes.

Recognized based on eDNA sequences only. Ruderaliaceae is currently monogeneric.

#### Ruderalia

Taxon classificationFungiRuderalialesRuderaliaceae

Tedersoo gen. nov.

2FD5110D-0131-591B-9339-C1E8BDB57A89

859051

##### Type species.

*Ruderalia cosmopolita* Tedersoo.

##### Diagnosis.

**Figure 35.**
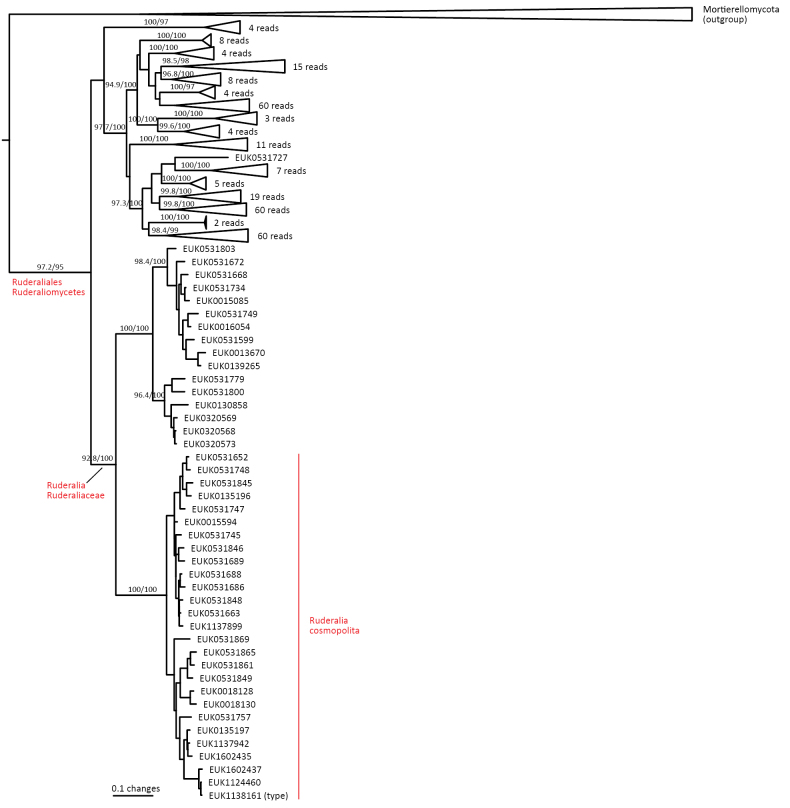
Maximum Likelihood SSU-ITS-LSU phylogram indicating the position of *Ruderalia cosmopolita* within Ruderaliomycetes, with ultra-rapid bootstrap values indicated (for higher-level classifications only). Other genus-level groups are collapsed. Mortierellomycota spp. were used as an outgroup.

Distinguishable from other fungi based on a diagnostic nucleotide signature in ITS2 (positions 209–222 aacgatagtgaagt; two mismatches allowed). Forms a monophyletic, least inclusive clade in Ruderaliaceae, covering sequences EUK1138161, EUK1137899, EUK0531800, and EUK1124460 (Figs 1, 35).

##### Notes.

Recognized based on eDNA sequences only. Comprises potentially three species represented by sequences EUK0531803 (cropland soil in Benin) and EUK0531800 (forest soil in Ghana).

#### Ruderalia cosmopolita

Taxon classificationFungiRuderalialesRuderaliaceae

Tedersoo sp. nov.

A73530CC-E521-5BD5-874C-FF06EA9B533E

859052

##### Diagnosis.

**Figure 36.**
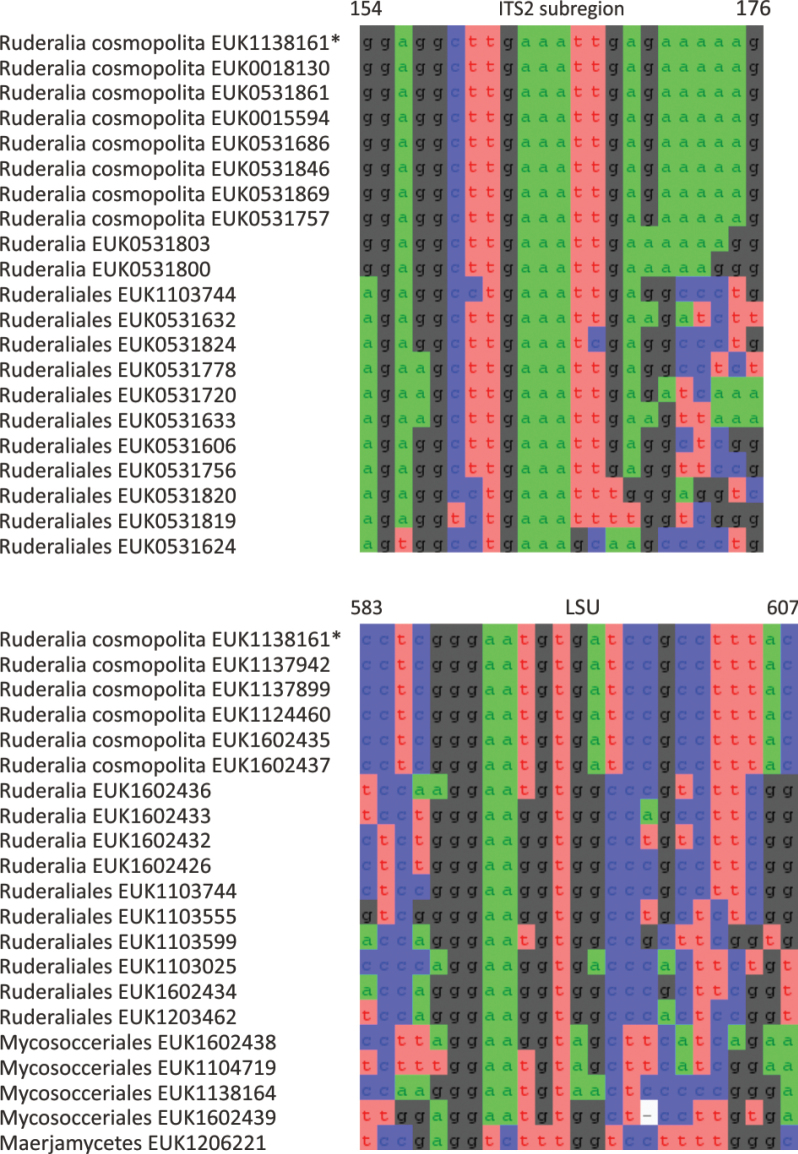
Diagnostic nucleotide sequences of *Ruderalia cosmopolita* relative to the closest related species in ITS2 and LSU. Numbers indicate positions in the legitype (marked with an asterisk).

Separation from other species of *Ruderalia* based on ITS2 (positions 154–176 ggaggcttgaaattgagaaaaag; one mismatch allowed) and LSU D2 (positions 583–607 cctcgggaatgtgatccgcctttac; one mismatch allowed) as indicated in Fig. 36. Intraspecific variation up to 9.8% (including up to 7% in the type locality) in ITS2 due to multiple microsatellite-like repeats and long homopolymers and up to 1.5% in LSU. Interspecific distance > 20% in ITS2.

##### Type.

Vouchered soil sample TUE028393 (**holotype**); eDNA sequence EUK1138161 = OZ253804 (**legitype**); eDNA sample TUE128393 (**nucleotype**); GSMc plot G5804, wasteland soil in Tartu, Estonia, 58.3816°N, 26.6916°E.

##### Description.

Other sequences: EUK1137942, EUK1137899 and EUK1124460 (type locality); EUK0018130 (GSMc plot S150, *Quercus woodland* soil in Jamestown, CA, USA, 37.8489, –120.581); EUK0531861 (temperate grassland soil in Murrietta, CA, USA, 33.5319, –117.2492°E), EUK0015594 (GSMc plot EO077, *Cistus* shrubland soil in Essouira, Morocco, 31.5136, –9.6542°E); EUK0531686 (GSMc plot G6066, subtropical *Vachellia* desert soil in Al Mudawih, Saudi Arabia, 25.8411°N, 39.2793°E); EUK0531846 (GSMc plot G6106, cropland soil in Betas, Iraqi Kurdistan, 37.0588°N, 42.7622°E); EUK0531869 (GSMc plot G5748, subtropical forest soil in Cebollati, Uruguay, –33.8292, –54.7672°E); and EUK0531757 (subtropical shrubland soil in Los Panguiles, Chile, –33.3822, –70.9636°E).

##### Etymology.

*Rudus* (Latin) refers to a common habitat in early successional land, and *cosmopolites* (Greek) refers to the global distribution.

##### Notes.

Found exclusively in soil in multiple habitats of Europe, Asia, North America, South America, and North Africa, with most records from anthropogenic and semidry shrubland habitats (n = 25 records). The 22 additional GlobalFungi records (21 from soil) support these findings.

#### Olpidiomyceta

Taxon classificationFungiRuderalialesRuderaliaceae

Tedersoo, Sanchez-Ramirez, Kõljalg, Bahram, M. Döring, Schigel, T.W. May, M. Ryberg & Abarenkov, Fungal Diversity 90: 150 (2018)

BF2354CF-BCC2-5551-B465-7944C8CE358D

554007

##### Type phylum.

Olpidiomycota Doweld.

##### Description.

As in Tedersoo et al. (2018).

##### Notes.

Currently harbors Olpidiomycota.

#### Olpidiomycota

Taxon classificationFungiFungiMucoromycota

Doweld, Index Fungorum 42: 1 (2013)

F3574A17-3B0A-5E59-AC01-304343BE78C7

550327

##### Type class.

Olpidiomycetes Doweld.

##### Description.

As in Doweld (2013).

##### Notes.

Currently harbors Olpidiomycetes, Bryolpidiomycetes (class. nov.), Chthonolpidiomycetes (class. nov.), and Savannolpidiomycetes (class. nov.).

#### Bryolpidiomycetes

Taxon classificationFungiMucoromycotaOlpidiomycota

Tedersoo class. nov.

8B01EF6B-B680-5E1B-B72F-DF9EBA853814

859053

##### Type order.

Bryolpidiales Tedersoo.

##### Diagnosis.

Distinguishable from other fungi based on a diagnostic nucleotide signature in LSU D1 (positions 169–183 in the type species and 167–181 in *S. cerevisiae* cgcggctgccgaagt or ggtcgcgaccgcggt; one mismatch allowed). Forms a monophyletic, least inclusive clade in Olpidiomycota, covering sequences EUK1124873, EUK1608195, EUK1186288, and EUK1186289 (Fig. 1).

##### Notes.

Recognized based on eDNA sequences only. Encoded as clade GS93G in EUKARYOME v1.9. Currently harbors Bryolpidiales (ord. nov.) and another potentially order-level group represented by sequences EUK1186288 and EUK1186289 (both forest soil in Altay Kray, Russian Federation). Comprises potentially 10–11 species. Detected in soil (88.5% out of the 26 records) and sediments (11.5%) in tundra to hot tropical biomes across all continents, including Sub-Antarctic islands.

#### Bryolpidiales

Taxon classificationFungiOlpidiomycotaBryolpidiomycetes

Tedersoo ord. nov.

40A362F4-82A0-5E77-8C70-FD24395E2B23

859056

##### Type family.

Bryolpidiaceae Esmaeilzadeh-Salestani.

##### Diagnosis.

Distinguishable from other fungi based on diagnostic nucleotide signatures in LSU D1 (positions 169–183 in type species and 167–181 in *S. cerevisiae* cgcggctgccgaagt or ggtcgcgaccgcggt; one mismatch allowed) and ITS2 (positions 102–113 in type species agngaacagcgg or aggcacggcagt; one mismatch allowed). Forms a monophyletic, least inclusive clade in Bryolpidiomycetes, covering sequences EUK1124873 and EUK1608195 (Fig. 1).

##### Notes.

Recognized based on eDNA sequences only. Currently includes Bryolpidiaceae (fam. nov.).

#### Bryolpidiaceae

Taxon classificationFungiBryolpidiomycetesBryolpidiales

Tedersoo fam. nov.

EFE68EAB-30B5-5D6E-99FB-A142E79AA7E8

859057

##### Type genus.

*Bryolpidium* Tedersoo.

##### Diagnosis.

Distinguishable from other fungi based on diagnostic nucleotide signatures in SSU V9 (positions 1706–1719 in *S. cerevisiae* gtcgagaagttatc; one mismatch allowed), ITS2 (positions 102–113 in type species agngaacagcgg; one mismatch allowed), and LSU D1 (positions 169–183 in type species and 167–181 in *S. cerevisiae* cgcggctgccgaagt; one mismatch allowed). Forms a monophyletic, least inclusive clade in Bryolpidiales, covering sequences EUK1124873 and EUK1608195 (Fig. 1).

##### Notes.

Recognized based on eDNA sequences only. Comprises *Bryolpidium* (gen. nov.).

#### Bryolpidium

Taxon classificationFungiBryolpidialesBryolpidiaceae

Tedersoo gen. nov.

CB3E75E1-6D0D-5435-8DD5-57003FB3FF6A

859058

##### Type species.

*Bryolpidium mundanum* Tedersoo.

##### Diagnosis.

**Figure 37.**
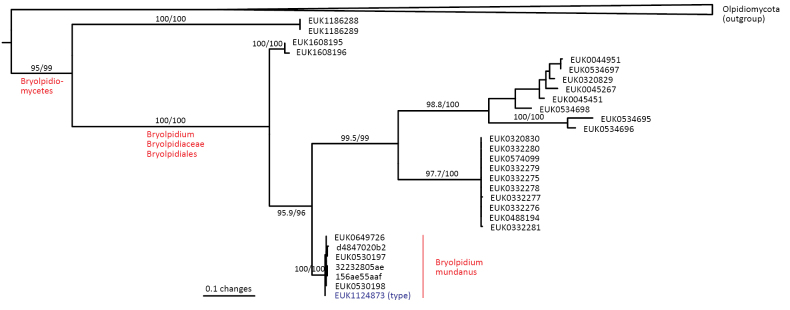
Maximum Likelihood SSU-ITS-LSU phylogram indicating the position of *Bryolpidium mundanum* within Bryolpidiomycetes, with ultra-rapid bootstrap values indicated. Other genus-level groups are collapsed. Olpidiomycota spp. were used as an outgroup. Abbreviations for GlobalFungi accessions: d4847020b2, d4847020b222e5b7830540d220e20499; 32232805ae, 32232805aef1d4510876e11cba753f7d; and 156ae55aaf, 156ae55aafdd258247d24e567a23cdf3.

Distinguishable from other fungi based on diagnostic nucleotide signatures in SSU V9 (positions 1706–1719 in *S. cerevisiae* gtcgagaagttatc; one mismatch allowed), ITS2 (positions 102–113 in type species agngaacagcgg; one mismatch allowed), and LSU D1 (positions 169–183 in type species and 167–181 in *S. cerevisiae* cgcggctgccgaagt; one mismatch allowed). Forms a monophyletic, least inclusive clade in Bryolpidiaceae, covering sequences EUK1124873 and EUK1608195 (Figs 1, 37).

##### Notes.

Recognized based on eDNA sequences only. Comprises 9–10 species represented by sequences EUK1608195 (forest soil in Morocco), EUK0044951 (grassland soil in Kyrgyzstan), EUK0534697 (forest soil in Pakistan), EUK0045451 (tundra soil in Leonie Island, Antarctica), EUK0320829 (lake sediment in Germany), EUK0534698 (grassland soil in Kyrgyzstan), EUK0574099 (river sediment in Scotland), and EUK0534695 (forest soil in Turkey).

#### Bryolpidium mundanum

Taxon classificationFungiBryolpidialesBryolpidiaceae

Tedersoo sp. nov.

DD346D1B-A97F-5C6B-A0E9-80B5DEEC1FB6

859060

##### Diagnosis.

**Figure 38.**
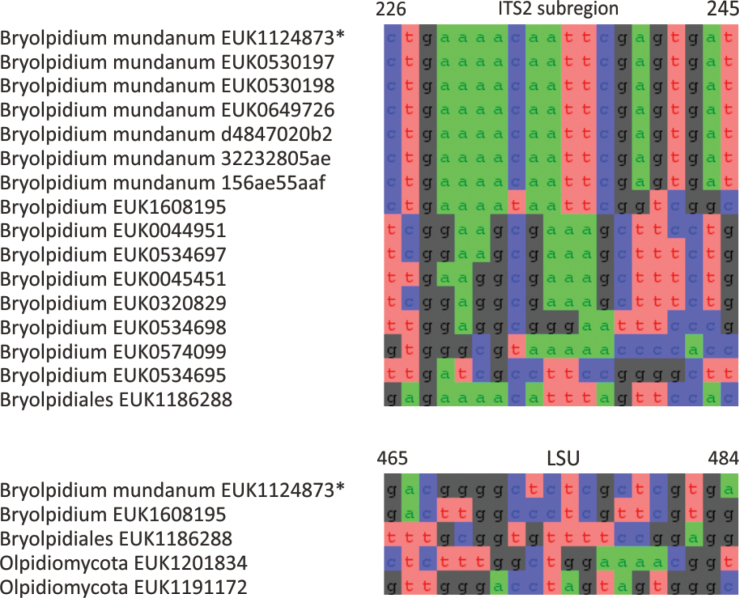
Diagnostic nucleotide sequences of *Bryolpidium mundanum* relative to the closest related species in ITS2 and LSU. Numbers indicate positions in the legitype (marked with an asterisk). Abbreviations for GlobalFungi accessions: d4847020b2, d4847020b222e5b7830540d220e20499; 32232805ae, 32232805aef1d4510876e11cba753f7d; and 156ae55aaf, 156ae55aafdd258247d24e567a23cdf3.

Separation from other species of *Bryolpidium* based on ITS2 (positions 226–245 ctgaaaacaattcgagtgat; no mismatch allowed) and LSU (positions 465–494 gacggggctctcgctcgtga; no mismatch allowed) as indicated in Fig. 38. Intraspecific variation up to 4.4% in ITS2. Interspecific distance > 20% in ITS2.

##### Type.

Vouchered soil sample TUE028510 (**holotype**); eDNA sequence EUK1124873 = OZ253805 (**legitype**); eDNA sample TUE128510 (**nucleotype**); GSMc plot G5911, wasteland soil in Tartu, Estonia, 58.3809°N, 26.6917°E.

##### Description.

Other sequences: EUK0530197 (GSMc plot G6091, subtropical desert soil in Al Zita, Saudi Arabia, 28.9243°N, 35.4438°E); EUK0530198 (urban park soil in Mildura, VIC, Australia, –34.1854°N, 142.1696°E); EUK0649726 (urban soil in Põlva, Estonia, 58.0666°N, 27.0939°E); and GlobalFungi records d4847020b222e5b7830540d220e20499 (subtropical woodland soil in El Tepeyac, San Luis Potosi, Mexico, 57.7165°N, 27.0549°E); 32232805aef1d4510876e11cba753f7d (temperate shrubland soil in Elche, Spain, 38.30, –0.72); and 156ae55aafdd258247d24e567a23cdf3 (temperate forest soil in Ait Tamlil, Morocco, 31.56, –6.99).

##### Etymology.

*>Bryum* (Greek and Latin) refers to its common habitat amongst mosses, and *mundanum* (Latin) refers to cosmopolitan distribution.

##### Notes.

Found in soil in urban (3 out of 4 records) and natural environments in Europe, the Arab Peninsula, and Australia. The soil habitat is supported by three additional GlobalFungi records from natural habitats in Spain, Morocco, and Mexico.

#### Chthonolpidiomycetes

Taxon classificationFungiMucoromycotaOlpidiomycota

Tedersoo class. nov.

636BADF2-5750-5821-8262-8E5AEE2541A5

859061

##### Type order.

Chthonolpidiales Tedersoo.

##### Diagnosis.

Distinguishable from other fungi based on diagnostic nucleotide signature in LSU D2 (positions 695–714 in type species and 604–623 in *S. cerevisiae* gactgcttgcaggctgcata; three mismatches allowed). Forms a monophyletic, least inclusive clade in Olpidiomycota, covering sequences EUK1124876, EUK0534818, EUK0534797, EUK1191212, and EUK1138033 (Fig. 1).

##### Notes.

Recognized based on eDNA sequences only. Encoded as clade GS93K in EUKARYOME v1.9. Currently harbors Chthonolpidiales (ord. nov.). Comprises potentially 25–30 species. Detected in soil (95.9% out of the 73 records) and mosses (4.1%) in tundra to hot tropical biomes across all continents except Antarctica.

#### Chthonolpidiales

Taxon classificationFungiOlpidiomycotaChthonolpidiomycetes

Tedersoo ord. nov.

1C91CB5B-5110-5017-92CB-DBA1E69B6915

859062

##### Type family.

Chthonolpidiaceae Tedersoo.

##### Diagnosis.

Distinguishable from other fungi based on a diagnostic nucleotide signature in LSU D2 (positions 695–714 in type species and 604–623 in *S. cerevisiae* gactgcttgcaggctgcata; two mismatches allowed). Forms a monophyletic, least inclusive clade in Chthonolpidiomycetes, covering sequences EUK1124876, EUK0534797, EUK0534798, EUK0534818, EUK1191212, and EUK1138033 (Fig. 1).

##### Notes.

Recognized based on eDNA sequences only. Currently includes Chthonolpidiaceae (fam. nov.).

#### Chthonolpidiaceae

Taxon classificationFungiChthonolpidiomycetesChthonolpidiales

Tedersoo fam. nov.

95D6714B-F697-50D9-A4C1-2A7B31B32C69

859063

##### Type genus.

*Chthonolpidium* Tedersoo.

##### Diagnosis.

Distinguishable from other fungi based on diagnostic nucleotide signatures in 5.8S (positions 142–154 in type species and 143–155 in *S. cerevisiae* tgttcgacaycc; one mismatch allowed) and LSU D2 (positions 695–714 in type species and 604–623 in *S. cerevisiae* gactgcttgcaggctgcata; one mismatch allowed). Forms a monophyletic, least inclusive clade in Chthonolpidiales, covering sequences EUK1124876, EUK0534797, EUK0534798, EUK0534818, EUK1191212, and EUK1138033 (Fig. 1).

##### Notes.

Recognized based on eDNA sequences only. Includes *Chthonolpidium* (gen. nov.) and potentially other genera represented by sequences EUK1191212 (forest soil in Puerto Rico), EUK0534797 (urban soil in China), EUK0534798 (grassland soil in Norway), and EUK0534818 (forest soil in Colombia).

#### Chthonolpidium

Taxon classificationFungiChthonolpidialesChthonolpidiaceae

Tedersoo gen. nov.

C235C11C-77EC-5A90-941A-59E7D337C4F3

859064

##### Type species.

*Chthonolpidium enigmatum* Tedersoo.

##### Diagnosis.

**Figure 39.**
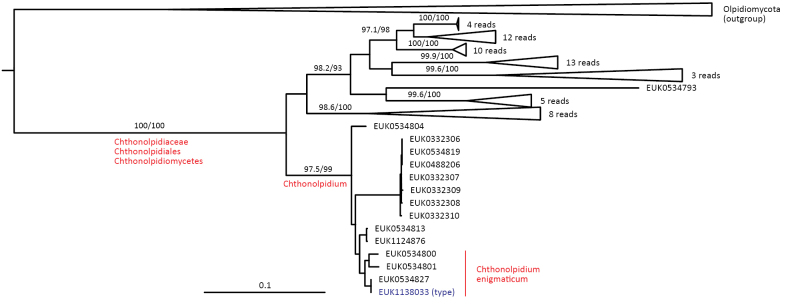
Maximum Likelihood SSU-ITS-LSU phylogram indicating the position of *Chthonolpidium enigmatum* within Chthonolpidiomycetes, with ultra-rapid bootstrap values indicated (for higher-level classifications only). Other genus-level groups are collapsed. Olpidiomycota spp. were used as an outgroup.

Distinguishable from other fungi based on diagnostic nucleotide signatures in ITS2 (positions 60–74 gggccaagctggtta; one mismatch allowed) and LSU D2 (positions 672–686 in type species and 604–618 in *S. cerevisiae* ttgcagttgggcgcc; one mismatch allowed). Forms a monophyletic, least inclusive clade in Chthonolpidiaceae, covering sequences EUK1124876 and EUK1138033 (Figs 1, 39).

##### Notes.

Recognized based on eDNA sequences only. Comprises potentially four species represented by sequences EUK1124876 (mosses in Estonia), EUK0534819 (tundra soil in AK, USA), and EUK0534804 (grassland soil in Tibet).

#### Chthonolpidium enigmatum

Taxon classificationFungiChthonolpidialesChthonolpidiaceae

Tedersoo sp. nov.

3303B4C9-4531-52FF-9F96-525C1F11041B

859065

##### Diagnosis.

**Figure 40.**
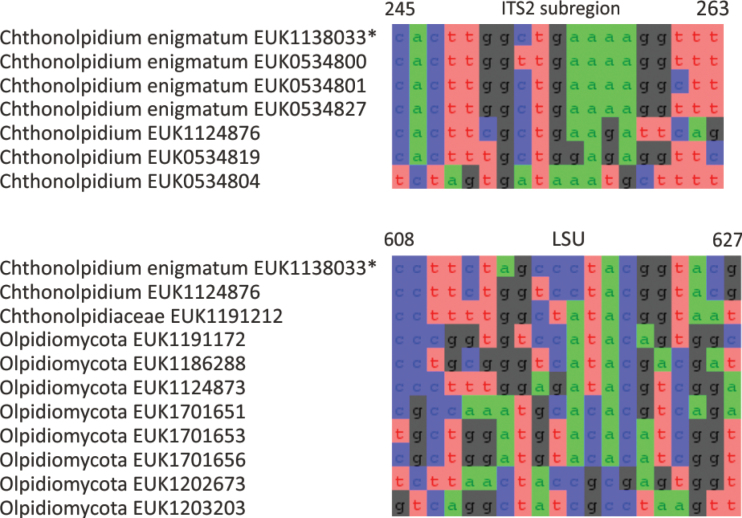
Diagnostic nucleotide sequences of *Chthonolpidium enigmatum* relative to the closest related species in ITS2 and LSU. Numbers indicate positions in the legitype (marked with an asterisk).

Separation from other species of *Chthonolpidium* based on ITS2 (positions 245–264 cacttggctgaaaaggttt; one mismatch allowed) and LSU (positions 608–627 ccttctagccctacggtacg; no mismatch allowed) as indicated in Fig. 40. Intraspecific variation up to 4.8% in ITS2. Interspecific distance at least 10.3% in ITS2.

##### Type.

Vouchered soil sample TUE028510 (**holotype**); eDNA sequence EUK1138033 = OZ253806 (**legitype**); eDNA sample TUE128510 (**nucleotype**); moss-dominated wasteland soil in Tartu, Estonia, 58.3808°N, 26.6917°E.

##### Description.

Other sequences: EUK0534827 (type location); EUK0534801 (temperate shrubland soil in Bliss, MI, USA); and EUK0534800 (GSMc plot G6167, subtropical shrubland soil in Al Hiwayb, Oman, 23.2140°N, 57.3337°E); and from GlobalFungi: 3949559c2adbb6dd485ee858c50aa75b (shrubland soil in Morocco, 33.9766, –3.3735°E); 170f6c5254bf08a8d3be2909670b3d85 (coniferous woodland soil in Utah, USA, 37.5819, –109.91); 3f89b0fffeb9329f6db10351b45fd923 (woodland rhizosphere soil in Spain, 37.888, –3.634); and 03a49631062976236cecf37723044ad8 (shrubland soil in Tunisia, 35.1678°N, 8.6738°E).

##### Etymology.

*>Khthonios* (Greek) refers to the common underground habitat, and *enigma* (Greek) means puzzling or mysterious.

##### Notes.

All four EUKARYOME and eight GlobalFungi records are derived from soil. Found in dry habitats in North Africa, Estonia, Spain, Oman, and the USA, indicating a cosmopolitan distribution.

#### Savannolpidiomycetes

Taxon classificationFungiMucoromycotaOlpidiomycota

Tedersoo & Esmaeilzadeh-Salestani class. nov.

E3DB8A35-7707-5D3B-9FA7-A468A5225D7B

859066

##### Type order.

Savannolpidiales Tedersoo & Esmaeilzadeh-Salestani.

##### Diagnosis.

Distinguishable from other fungi based on diagnostic nucleotide signatures in SSU V8 (positions 1546–1565 in *S. cerevisiae* gagcattgcaactattgctc; one mismatch allowed) and LSU D2 (positions 525–534 in the type species and 472–481 in *S. cerevisiae* gtgcactttt; one mismatch allowed). Forms a monophyletic, least inclusive clade in Olpidiomycota, covering sequences EUK1191172, EUK1191209, EUK1191210, EUK0534704, EUK1701673, EUK1701672, EUK1124874, and EUK1124875 (Fig. 1).

##### Notes.

Recognized based on eDNA sequences only. Encoded as clade GS93J in EUKARYOME v1.9. Currently harbors Savannolpidiales (ord. nov.) and potentially an order-level group represented by sequence EUK1191172 (forest soil in Taiwan). Comprises potentially 50–70 species, which are difficult to delimit because of multiple indels rather than substitutions in the ITS region and no clear barcoding gap. Detected in soil (94.8% out of the 191 records) and sediments (5.2%) in tundra to hot tropical biomes across all continents except Antarctica. Relatively common in Europe (51.8% of records) but less common in tropical biomes (15.7%).

#### Savannolpidiales

Taxon classificationFungiOlpidiomycotaSavannolpidiomycetes

Tedersoo & Esmaeilzadeh-Salestani ord. nov.

8308B450-488C-50FE-B24A-E534FED886B0

859068

##### Type family.

Savannolpidiaceae Tedersoo & Esmaeilzadeh-Salestani.

##### Diagnosis.

Distinguishable from other fungi based on diagnostic nucleotide signatures in the ITS2-LSU interface (LSU positions –2–18 in type species and *S. cerevisiae* aagtgatctgaaatcagaca; two mismatches allowed) and SSU V8 (positions 1589–1608 in *S. cerevisiae* atgattcatcagatcatgct; two mismatches allowed). Forms a monophyletic, least inclusive clade in Savannolpidiomycetes, covering sequences EUK1191209, EUK1191210, EUK0534704, EUK1124874, EUK1701673, EUK1701672, and EUK1124875 (Fig. 1).

##### Notes.

Recognized based on eDNA sequences only. Currently includes Savannolpidiaceae (fam. nov.).

#### Savannolpidiaceae

Taxon classificationFungiSavannolpidiomycetesSavannolpidiales

Tedersoo & Esmaeilzadeh-Salestani fam. nov.

17B16EC9-CD0F-57F7-BBB4-4B77104B0317

859069

##### Type genus.

*Savannolpidium* Tedersoo & Esmaeilzadeh-Salestani.

##### Diagnosis.

Distinguishable from other fungi based on diagnostic nucleotide signatures in the ITS2-LSU interface (LSU positions –2–18 in type species and *S. cerevisiae* aagtgatctgaaatcagaca; one mismatch allowed) and ITS2 (positions 137–151 in type species gcgtactccttgtcc; two mismatches allowed) and SSU V8 (positions 1589–1608 in *S. cerevisiae* atgattcatcagatcatgct; one mismatch allowed). Forms a monophyletic, least inclusive clade in Savannolpidiales, covering sequences EUK1191209, EUK1191210, EUK0534704, EUK1124874, EUK1701673, EUK1701672, and EUK1124875 (Fig. 1).

##### Notes.

Recognized based on eDNA sequences only. Savannolpidiaceae includes *Savannolpidium* (gen. nov.) and other potential genera represented by sequences EUK1701673 (forest soil in Madeira), EUK1701672 (woodland soil in Benin), EUK0534781 (forest soil in Iraqi Kurdistan), EUK1124875 (forest soil in Estonia), EUK1191209 (forest soil in Puerto Rico), and EUK0534704 (forest soil in Turkey).

#### Savannolpidium

Taxon classificationFungiSavannolpidialesSavannolpidiaceae

Tedersoo & Esmaeilzadeh-Salestani gen. nov.

A04943B3-E22E-52C7-8C86-1297A3BE7DCA

859070

##### Type species.

*Savannolpidium raadiense* Tedersoo & Esmaeilzadeh-Salestani.

##### Diagnosis.

**Figure 41.**
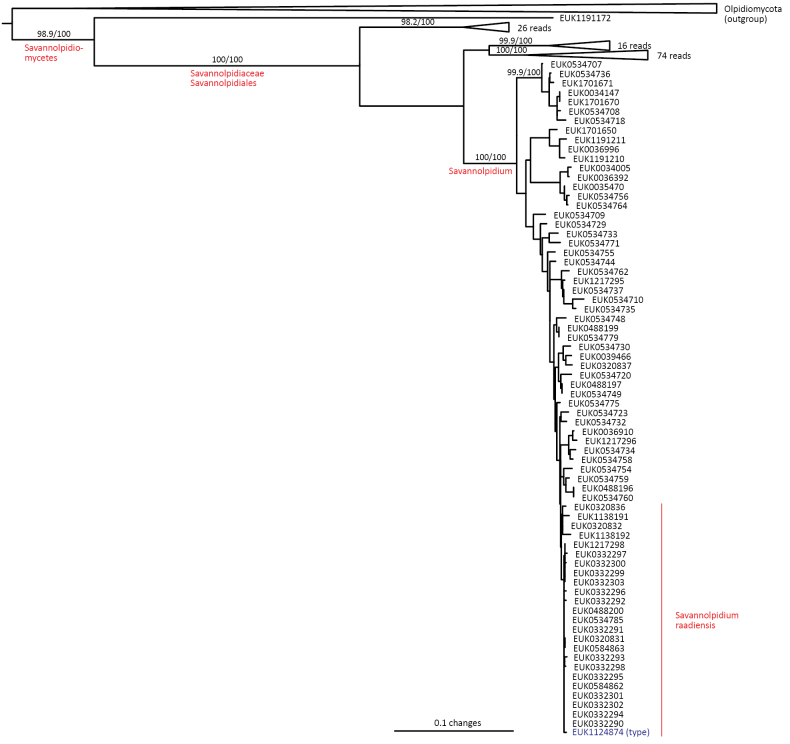
Maximum Likelihood SSU-ITS-LSU phylogram indicating the position of *Savannolpidium raadiense* within Savannolpidiomycetes, with ultra-rapid bootstrap values indicated (for higher-level classifications mainly). Other genus-level groups are collapsed. Olpidiomycota spp. were used as an outgroup.

Distinguishable from other fungi based on diagnostic nucleotide signatures in ITS2 (positions 121–135 in type species grtagtaaaagtagc; one mismatch allowed), SSU V9 (positions 1684–1693 in *S. cerevisiae* ccttttttyg; one mismatch allowed), and LSU D2 (positions 719–728 in type species and 604–613 in *S. cerevisiae* caaaaggatt; one mismatch allowed). Forms a monophyletic, least inclusive clade in Savannolpidiaceae, covering sequences EUK1124874 and EU1191210 (Figs 1, 41).

##### Notes.

Recognized based on eDNA sequences only. Comprises about 15–25 species represented by sequences EUK1217296 (grassland soil in Austria), EUK0034005 (forest soil in South Africa), EUK0036392 (forest soil in South Africa), EUK0034147 (woodland soil in New Caledonia), EUK0036996 (forest soil in Puerto Rico), EUK0534723 (forest soil in South Africa), EUK0534729 (forest soil in Spain), EUK0036910 (forest soil in Estonia), and EUK0534732 (forest soil in Iran).

#### Savannolpidium raadiense

Taxon classificationFungiSavannolpidialesSavannolpidiaceae

Tedersoo & Esmaeilzadeh-Salestani sp. nov.

532E9F06-099A-53DA-BA96-D10380A0B08B

859071

##### Diagnosis.

**Figure 42.**
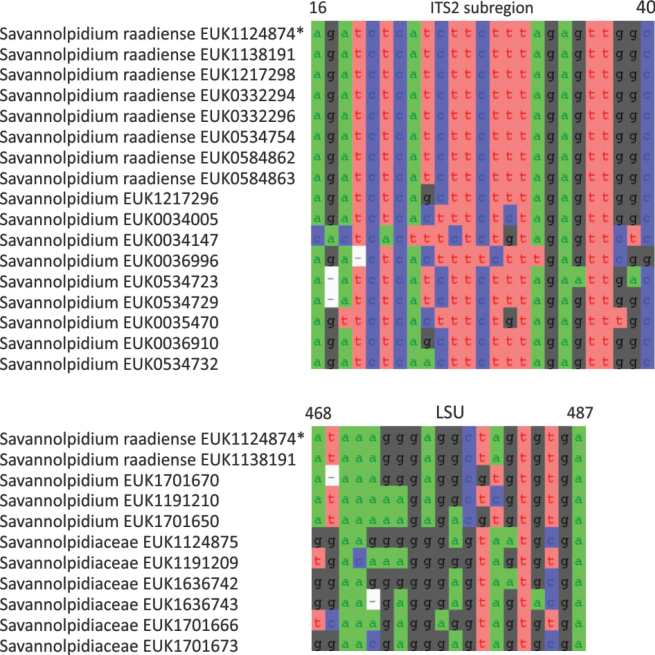
Diagnostic nucleotide sequences of *Savannolpidium raadiense* relative to the closest related species in ITS2 and LSU. Numbers indicate positions in the legitype (marked with an asterisk).

Separation from other species of *Savannolpidium* based on ITS2 (positions 16–40 agatctcatcttctttagagttggc; no mismatch allowed) and LSU (positions 468–492 tataaagggaggctagtgtgagcgc; no mismatch allowed) as indicated in Fig. 42. Intraspecific variation up to 1.6% in ITS2 and 0.9% in LSU. Interspecific distance at least 2.8% in ITS2.

##### Type.

Vouchered soil sample TUE002210 (**holotype**); eDNA sequence EUK1124874 = OZ253807 (**legitype**); eDNA sample TUE102210 (**nucleotype**); GSMc plot G5233, *Populus balsamifera*-dominated wasteland soil in Raadi, Estonia, 58.3972°N, 26.7693°E.

##### Description.

Other sequences: EUK1138191 (type locality); EUK1217298 (GSMc plot G4777, flooded grassland soil Suur-Pakri Härs-hämani, Estonia, 59.3310°N, 23.9272°E); EUK0332294 (GSMc plot G5899, dry *Juniperus* shrubland soil in Virtsu, Estonia, 58.5775°N, 23.5547°E); EUK0332296 (flooded grassland soil in Haanja, Estonia, 57.7165°N, 27.0549°E); EUK0534754 (GSMc plot G6107, Quercus woodland soil in Armishte, Iraqi Kurdistan, 37.0468°N, 42.8049°E); EUK0584862 (FunAqua river sediment sample W1356s, Spey, Scotland, 57.0552, –4.1276°E); EUK0584863 (FunAqua saltwater sediment sample W0938s, Laguna di Orbetello, Italy, 42.4296°N, 11.1988°E).

##### Etymology.

*Savanna* (Taino) refers to treeless grasslands, and *Raadi* (Estonian) refers to the type locality.

##### Notes.

Found in grassy and disturbed habitats and aquatic sediments in Europe and the Middle East (n = 25 records). This is supported by 18 additional GlobalFungi records from agricultural and grassland soils in Europe.

#### Rozellomyceta

Taxon classificationFungiFungiFungi

Tedersoo, Sanchez-Ramirez, Kõljalg, Bahram, M. Döring, Schigel, T.W. May, M. Ryberg & Abarenkov, Fungal Diversity 90: 147 (2018)

52332B84-FF12-5789-A6A6-76801B58DE74

553988

##### Type phylum.

Rozellomycota Doweld.

##### Description.

As in Tedersoo et al. (2018).

##### Notes.

*Rozellomyceta* currently harbors Rozellomycota.

#### Rozellomycota

Taxon classificationFungiFungiRozellomyceta

Doweld, Index Fungorum 43:1 (2013)

EDEC1953-2BAA-5A76-B1C8-DD6A00BFE92F

563383

##### Type class.

None.

##### Description.

As in Tedersoo et al. (2018).

##### Notes.

Currently harbors Rozellomycetes, *Microsporidea*, Gelotisporidiomycetes (class. nov.), and Sumavosporidiomycetes (class. nov.).

#### Gelotisporidiomycetes

Taxon classificationFungiRozellomycetaRozellomycota

Tedersoo class. nov.

844911DB-3E0F-52D7-945D-EB9DC375F6EB

859077

##### Type order.

Gelotisporidiales Tedersoo.

##### Diagnosis.

Distinguishable from other fungi based on diagnostic nucleotide signatures in SSU V8 (positions 1541–1550 in *S. cerevisiae* ggatcagtca; no mismatch allowed) and LSU D1 (positions 302–311 in type species and 305–314 in *S. cerevisiae* cgcgccatct; one mismatch allowed). Forms a monophyletic, least inclusive clade in Rozellomycota, covering sequences EUK1138731, EUK1138718, EUK1138568, EUK1138757, EUK1100925, EUK1105586, EUK1105726, EUK1105789, EUK1101184, EUK1202629, EUK1201985, EUK1104844, and EUK1123671 (Fig. 1).

##### Notes.

Recognized based on eDNA sequences only. Encoded as clade GS15 in EUKARYOME v1.9. Previously considered a lineage with phylogenetic affinities to Blastocladiomycota, but inclusive taxon sampling in Blastocladiomycota and Rozellomycota places Gelotisporidiomycetes in Rozellomycota. Currently harbors Gelotisporidiales (ord. nov.). Comprises potentially 90–110 species. Detected in soil (96.7% out of 335 records) and freshwater (2.7%). Two samples were identified from myxomycete colonies, suggesting that this group may include protist parasites. Recorded from tundra to hot tropical biomes across all continents except Antarctica.

#### Gelotisporidiales

Taxon classificationFungiRozellomycotaGelotisporidiomycetes

Tedersoo ord. nov.

4EC045F1-8621-5E86-A344-A54F79E587B4

869078

##### Type family.

Gelotisporidiaceae Tedersoo.

##### Diagnosis.

Distinguishable from other fungi based on diagnostic nucleotide signatures in SSU V8 (positions 1541–1550 in *S. cerevisiae* ggatcagtca; no mismatch allowed) and LSU D1 (positions 302–311 in the type species and 305–314 in *S. cerevisiae* cgcgccatct; one mismatch allowed). Forms a monophyletic, least inclusive clade in Gelotisporidiomycetes, covering sequences EUK1138731, EUK1138718, EUK1138568, EUK1138757, EUK1100925, EUK1105586, EUK1105726, EUK1105789, EUK1101184, EUK1202629, EUK1201985, EUK1104844, and EUK1123671 (Fig. 1).

##### Notes.

Recognized based on eDNA sequences only. Currently includes Gelotisporidiaceae (fam. nov.) and other potentially family-level groups represented by sequences EUK1100925 (unspecified soil in Tibet), EUK1105586 (lake water in Sweden), EUK1105726 (forest soil in Sweden), and EUK1105789 (forest soil in Sweden).

#### Gelotisporidiaceae

Taxon classificationFungiGelotisporidiomycetesGelotisporidiales

Tedersoo fam. nov.

B148F9AE-45E4-5E2A-927A-7BCA24039FC6

859079

##### Type genus.

*Gelotisporidium* Tedersoo.

##### Diagnosis.

Distinguishable from other fungi based on a diagnostic nucleotide signature in LSU D2 (positions 613–627 in type species and 692–696 in *S. cerevisiae* cccttgggcgcaaag; one mismatch allowed). Forms a monophyletic, least inclusive clade in Gelotisporidiales, covering sequences EUK1138568, EUK1138757, EUK1100418, EUK1138778, EUK1202629, EUK1201985, EUK1104844, EUK1101158, and EUK1123671 (Fig. 1).

##### Notes.

Recognized based on eDNA sequences only. Includes *Gelotisporidium* and several genus-level groups represented by sequences EUK1138568 (forest soil in New Zealand), EUK1138757 (forest soil in New Zealand), EUK1100418 (permafrost in Canada), EUK1138778 (forest soil in New Zealand), EUK1202629 (forest soil in Finland), and EUK1123671 (forest soil in Estonia).

#### Gelotisporidium

Taxon classificationFungiGelotisporidialesGelotisporidiaceae

Tedersoo gen. nov.

FCE220F0-32B3-5469-9805-B7C9B8AD4896

859081

##### Type species.

*Gelotisporidium boreale* Tedersoo.

##### Diagnosis.

**Figure 43.**
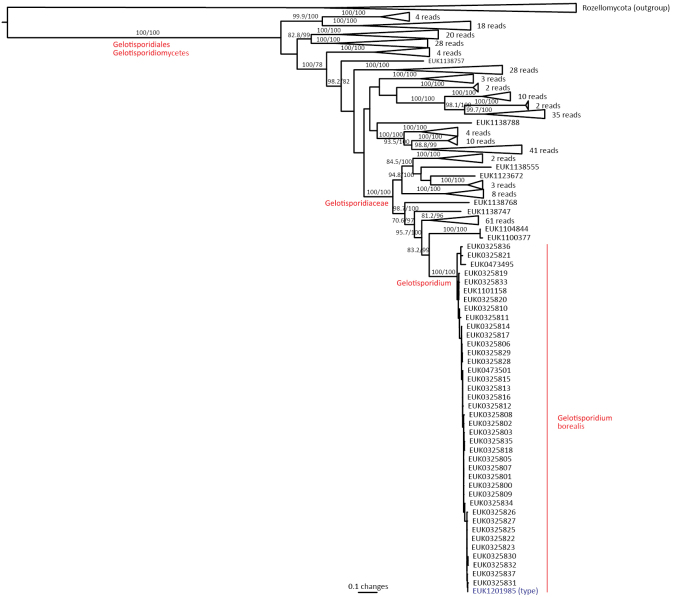
Maximum Likelihood SSU-ITS-LSU phylogram indicating the position of *Gelotisporidium boreale* within Gelotisporidiomycetes, with ultra-rapid bootstrap values indicated (for higher-level classifications only). Other genus-level groups are collapsed. Rozellomycota spp. were used as an outgroup.

Distinguishable from other fungi based on diagnostic nucleotide signatures in SSU V9 (positions 1699–1718 in *S. cerevisiae* acccgtctttcgttg; one mismatch allowed) and 5.8S-ITS2 (positions starting from 151 in type species and 153 in *S. cerevisiae* agaattgaaa; one mismatch allowed). Forms a monophyletic, least inclusive clade in Gelotisporidiaceae, covering sequences EUK1201985 and EUK1101158 (Figs 1, 43).

##### Notes.

Recognized based on eDNA sequences only. Comprises *Gelotisporidium boreale* (sp. nov.).

#### Gelotisporidium boreale

Taxon classificationFungiGelotisporidialesGelotisporidiaceae

Tedersoo sp. nov.

B8D340E7-3DEB-58D2-980E-F95EDC245443

859083

##### Diagnosis.

**Figure 44.**
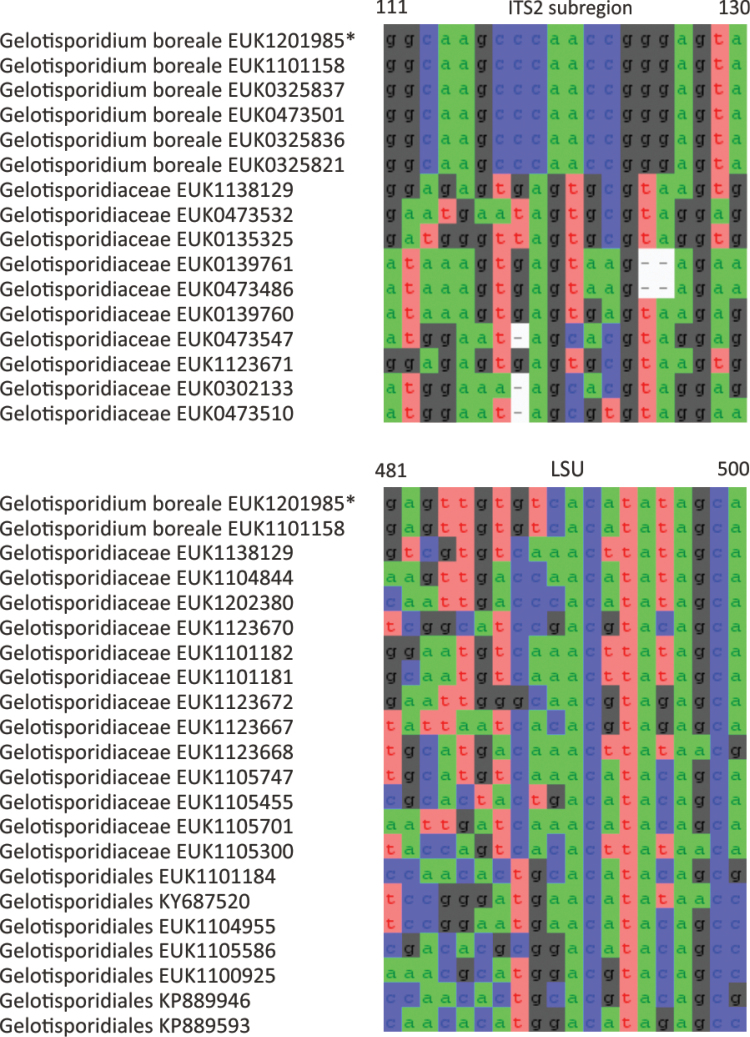
Diagnostic nucleotide sequences of *Gelotisporidium boreale* relative to the closest related species in ITS2 and LSU. Numbers indicate positions in the legitype (marked with an asterisk).

Separation from other species of Gelotisporidiaceae based on ITS2 (positions 111–130 ggcaagcccaaccgggagta; one mismatch allowed) and LSU (positions 481–500 gagttgtgtcacatatagca; one mismatch allowed) as indicated in Fig. 44. Intraspecific variation up to 4.7% in ITS2 and up to 1.0% in LSU. Interspecific distance > 15% in ITS2 and > 10% in LSU.

##### Type.

Vouchered soil sample TUE000189 (**holotype**); eDNA sequence EUK1201985 = OZ253809 (**legitype**); eDNA sample TUE100189 (**nucleotype**); GSMc plot G2836, *Betula* spp. dominated tundra soil in Gelotjávri, Finland, 68.6035°N, 21.7452°E.

##### Description.

Other sequences: EUK1101158 (coniferous forest soil in Hofors, Sweden, 60.49°N, 16.3°E); EUK0325837 (GSMc plot S1124, mixed forest soil in Zavodoukovskiy, Tyumen Oblast, Russian Federation, 56.5299°N, 66.5028°E); EUK0473501 (GSMc plot IHPR02, *Betula pubescens* tundra soil in Stora Sjöfallet, Sweden, 67.6367°N, 17.8216°E); EUK0325836 (*Betula pubescens* tundra soil at Lake Sobach’ye, Krasnoyarsk Krai, Russian Federation, 69.0033°N, 90.9875°E); and EUK0325821 (GSMc plot S1081, *Araucaria araucana* forest soil in Nahuelbuta, Chile, –37.7897, –73.0034°E).

##### Etymology.

*Gelot* (Sámi) refers to the type locality at Gelotjávri (Kelottijärvi), and *boreale* (Latin) refers to the mainly boreal habitat of the species.

##### Notes.

Found in 40 soil samples in boreal and subarctic habitats in Fennoscandia, the Northern Russian Federation, and Alaska, and once in the Chilean highlands (has unique substitutions). The 27 additional GlobalFungi records indicate habitat in soil and dead wood (11.1%) and distribution in the Holarctic realm.

#### Sumavosporidiomycetes

Taxon classificationFungiMucoromycotaRozellomycota

Tedersoo & Esmaeilzadeh-Salestani class. nov.

38C0201E-8A1C-582A-AB5F-D2F35ED529AA

859072

##### Type order.

Sumavosporidiales Tedersoo & Esmaeilzadeh-Salestani.

##### Diagnosis.

Distinguishable from other fungi based on a diagnostic nucleotide signature in SSU V6 (positions 1153–1167 gagcacaccaaragt or gacgacacaagaagt in *S. cerevisiae*; no mismatch allowed). Forms a monophyletic, least inclusive clade in Rozellomycota, covering sequences EUK1206927, EUK1202246, EUK1200658, UDB029033, EUK1105717, EUK1107386, EUK1106576, EUK1101061, and EUK1101529 (Fig. 1).

##### Notes.

Recognized based on eDNA sequences only. Encoded as clade GS01 in EUKARYOME v1.9. Previously considered a distinct phylum-level lineage, but inclusive taxon sampling in Rozellomycota places Sumavosporidiomycetes in this phylum. Currently harbors Sumavosporidiales (ord. nov.). Comprises potentially 800–1050 species. Members of this class have been detected from soil (99.5% out of 4122 records), sediments (0.3%), and water (0.1%). Recorded from high arctic to hot tropical biomes across all continents, including Antarctica.

#### Sumavosporidiales

Taxon classificationFungiRozellomycotaSumavosporidiomycetes

Tedersoo & Esmaeilzadeh-Salestani ord. nov.

3A792B94-1CDB-5E6E-A9CB-C500633FB3B4

859073

##### Type family.

Sumavosporidiaceae Tedersoo & Esmaeilzadeh-Salestani.

##### Diagnosis.

Distinguishable from other fungi based on a diagnostic nucleotide signature in SSU V6 (positions 1157–1176 in *S. cerevisiae* acaccaaaagtggattttgc or acaccaagagtggagcatgc or acacaagaagtggagcctgc; one mismatch allowed). Forms a monophyletic, least inclusive clade in Sumavosporidiomycetes, covering sequences EUK1206927, EUK1202246, EUK1200658, UDB029033, EUK1105717, EUK1107386, EUK1106576, EUK1101061, and EUK1101529 (Fig. 1).

##### Notes.

Recognized based on eDNA sequences only. Currently includes Sumavosporidiaceae (fam. nov.) and several potentially family-level groups represented by sequences EUK1206927 (marine sediment in Norway), EUK1202246 (river sediment in Slovenia), EUK1200658 (forest soil in Bulgaria), EUK1101529 (forest soil in Sweden), EUK1105717 (forest soil in Sweden), and EUK1101061 (mixed soil in Tibet).

#### Sumavosporidiaceae

Taxon classificationFungiSumavosporidiomycetesSumavosporidiales

Tedersoo & Esmaeilzadeh-Salestani fam. nov.

35296339-9E31-5BD4-A4A5-F8B5BE91F994

859074

##### Type genus.

*Sumavosporidium* Tedersoo & Esmaeilzadeh-Salestani.

##### Diagnosis.

Distinguishable from other fungi based on a diagnostic nucleotide signature in 5.8S (positions 124–138 in the type species and 125–139 in *S. cerevisiae* gcaatcygcaggcat; one mismatch allowed). Forms a monophyletic, least inclusive clade in Sumavosporidiales, covering sequences UDB029033, UDB029043, UDB029027, UDB028954, EUK1104656, EUK1106151, and EUK1104875 (Fig. 1).

##### Notes.

Recognized based on eDNA sequences only. Includes *Sumavosporidium* (gen. nov.).

#### Sumavosporidium

Taxon classificationFungiSumavosporidialesSumavosporidiaceae

Tedersoo & Esmaeilzadeh-Salestani gen. nov.

006E10AB-BF53-574B-B8D6-111E43179016

859075

##### Type species.

*Sumavosporidium sylvestre* Tedersoo & Esmaeilzadeh-Salestani.

##### Diagnosis.

**Figure 45.**
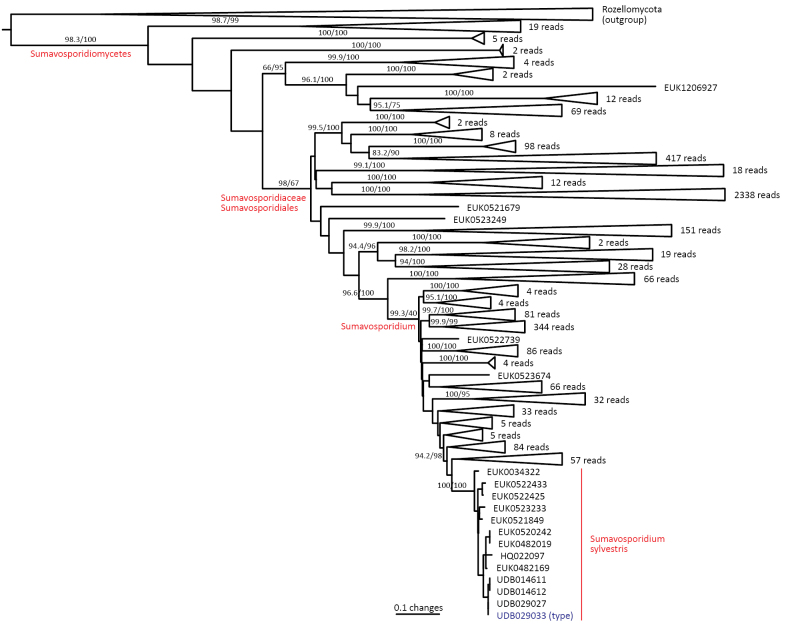
Maximum Likelihood SSU-ITS-LSU phylogram indicating the position of *Sumavosporidium sylvestre* within Sumavosporidiomycetes, with ultra-rapid bootstrap values indicated (for higher-level classifications mainly). Other genus-level groups are collapsed. Rozellomycota spp. were used as an outgroup.

Distinguishable from other fungi based on a diagnostic nucleotide signature in 5.8S (positions 124–138 in the type species and 125–139 in *S. cerevisiae* gcaatcygcaggcat; one mismatch allowed). Forms a monophyletic, least inclusive clade in Sumavosporidiaceae, covering sequences UDB029033, UDB029043, UDB029027, UDB028954, and EUK1104656 (Figs 1, 45).

##### Notes.

Recognized based on eDNA sequences only. Comprises potentially 160–180 species represented by sequences UDB029043 (forest soil in Argentina), UDB028927 (woodland soil in Greece), UDB029030 (forest soil in Scotland), EUK1104656 (forest soil in Sweden), EUK0481687 (grassland soil in Norway), EUK1106151 (peatland soil in Sweden), UDB028954 (forest soil in Argentina), EUK0481807 (forest soil in Argentina), EUK0022003 (forest soil in OR, USA), EUK0481723 (forest soil in Magadan, Russian Federation), and EUK0481554 (forest soil in Chukotka, Russian Federation).

#### Sumavosporidium sylvestre

Taxon classificationFungiSumavosporidialesSumavosporidiaceae

Tedersoo & Esmaeilzadeh-Salestani sp. nov.

2E70C63A-1BB0-53A5-B3FC-3A332E8A0A5A

859076

##### Diagnosis.

**Figure 46.**
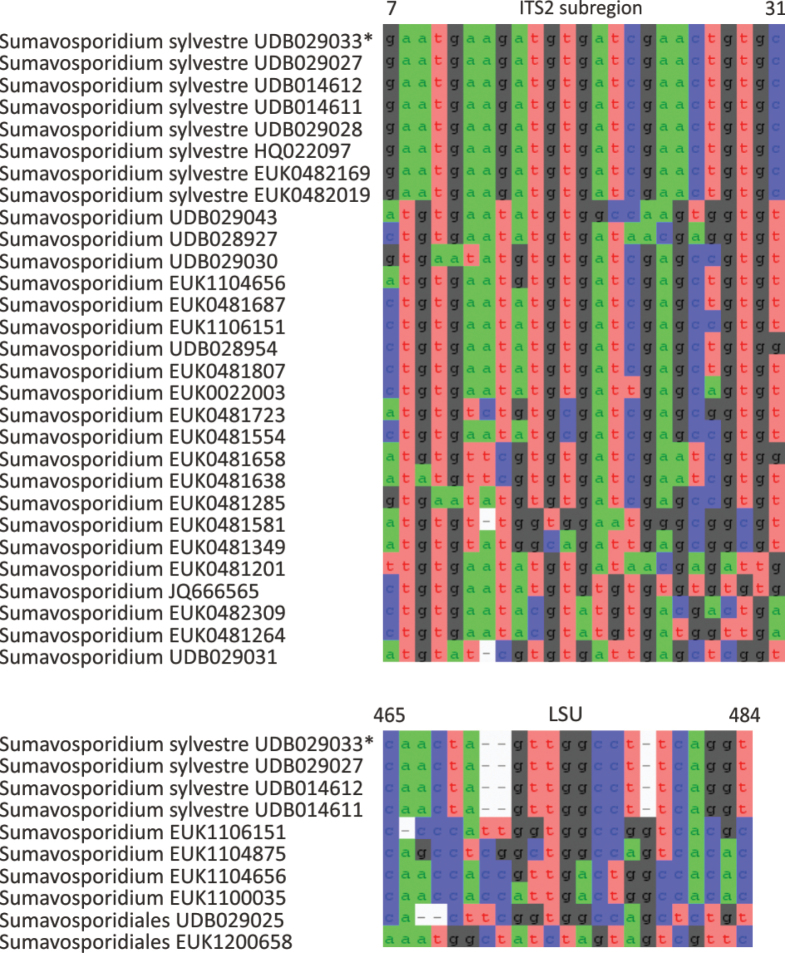
Diagnostic nucleotide sequences of *Sumavosporidium sylvestre* relative to the closest related species in ITS2 and LSU. Numbers indicate positions in the legitype (marked with an asterisk).

Separation from other species of *Sumavosporidium* based on ITS2 (positions 7–31 gaatgaagatgtgatcgaactgtgc; one mismatch allowed) and LSU (positions 465–484 caactagttggccttcaggt; one mismatch allowed) as indicated in Fig. 46. Intraspecific variation up to 2.0% in ITS2 and up to 0.2% in LSU. Interspecific distance at least 12.0% in ITS2.

##### Type.

Vouchered soil sample TUE000381 (**holotype**); eDNA sequence UDB029033 = OZ253808 (**legitype**); eDNA sample TUE100381 (**nucleotype**); GSMc plot S114, *Picea abies* dominated forest soil in *Šumava*, Czechia, 49.017°N, 13.4751°E.

##### Description.

Other sequences: UDB014611 and EUK0482169 (type locality); UDB029027, UDB029028 and UDB014612 (GSMc plot S121, *Fagus sylvatica* forest soil in Taunus, Germany, 50.1413°N, 8.2677°E); EUK0482019 and EUK0520242 (GSMc plot S426, *Fagus sylvatica* forest soil in Kistrupvang, Denmark, 56.0264°N, 12.3364°E); HQ022097 (mixed forest soil in Bartlett Experimental Forest, NH, USA, 44.06, –71.30); EUK0522425 and EUK0522433 and (GSMc plot G4145, mixed deciduous forest soil in Promised Land, PA, USA, 41.30491, –75.2021°E); EUK0523233 (GSMc plot S878, *Alnus alnobetula* tundra soil in Anadyr, Chukotka, Russia, 64.7219°N, 177.4238°E); EUK0034322 (GSMc plot G4713, *Tsuga mertensiana* forest soil in Crater Lake, OR, USA, 42.9786, –122.13); and EUK0521849 (GSMc plot S892, forest tundra soil in Arman, Magadan, Russia, 59.6972°N, 150.4118°E).

##### Etymology.

*Šumava* (Czech) refers to the type locality, and *sylva* (Latin) refers to the forest habitat.

##### Notes.

Found in eight localities in acidic temperate and boreal forest and tundra soils in Europe, Asia, and North America. GlobalFungi reveals seven additional records from forest soil in Europe and one record from an Indonesian oil palm plantation soil.

#### Zoopagomyceta

Taxon classificationFungiSumavosporidialesSumavosporidiaceae

Tedersoo, Sanchez-Ramirez, Kõljalg, Bahram, M. Döring, Schigel, T.W. May, M. Ryberg & Abarenkov, Fungal Diversity 90: 150 (2018)

F7FBB75A-57BB-5A80-9C79-3A066ED67037

554008

##### Type phylum.

Zoopagomycota Gryganskyi, M.E. Smith, Spatafora & Stajich, Mycologia 108 (5): 1035 (2016) [816300].

##### Description.

As in Tedersoo et al. (2018)

##### Notes.

Currently harbors Entomophthoromycota, Kickxellomycota, Zoopagomycota, Aldinomycota (phyl. nov.), Borikeniomycota (phyl. nov.), Mirabilomycota (phyl. nov.), Nematovomycota (phyl. nov.), Viljandiomycota (phyl. nov.), and Waitukubulimycota (phyl. nov.).

#### Kickxellomycota

Taxon classificationFungiFungiMucoromycota

Tedersoo, Sanchez-Ramirez, Kõljalg, Bahram, M. Döring, Schigel, T.W. May, M. Ryberg & Abarenkov, Fungal Diversity 90: 150 (2018)

17801D1C-D815-54A2-A913-A227945F743E

554009

##### Type class.

Kickxellomycetes Tedersoo, Sánchez-Ramirez, Kõljalg, Bahram, M. Döring, Schigel, T.W. May, M. Ryberg & Abarenkov.

##### Description.

As in Tedersoo et al. 2018.

##### Notes.

Kickxellomycota currently harbors Asellariomycetes, Barbatosporomycetes, Dimargaritomycetes, Harpellomycetes, Kickxellomycetes, Ramicandelaberomycetes, and Parakickxellomycetes (class. nov.).

#### Parakickxellomycetes

Taxon classificationFungiMucoromycotaKickxellomycota

Tedersoo class. nov.

CBF6F269-FC88-56AE-908E-FC1807888A25

859086

##### Type order.

Parakickxellales Tedersoo.

##### Diagnosis.

Separation from other fungi based on diagnostic nucleotide signatures in SSU V8 (positions 1476–1495 in *S. cerevisiae* ccaagkcaacgagtytacaa; two mismatches allowed) and LSU D1 (positions 184–198 in type species and 180–194 in *S. cerevisiae* ggtataatttgcctg; two mismatches allowed). Forms a monophyletic, least inclusive clade in Kickxellomycota, covering sequences EUK1189320, EUK1189314, EUK1100806, EUK1189309, UDB014747, EUK1107625, EUK1105293, EUK1189310, EUK1700155, EUK1189325, EUK1189316, and EUK1189321 (Fig. 1).

##### Notes.

Recognized based on eDNA sequences only. Encoded as clade GS19 in EUKARYOME v1.9. Currently harbors Parakickxellales. Comprises potentially 1150–1250 species. Detected in soil (98.6% out of 1597 records), sediments (1.2%), and water (0.2%). Found in arctic tundra to tropical biomes across all continents except Antarctica, but present on Subantarctic islands. Relatively more common and diverse in the neotropics (37.3% of records).

#### Parakickxellales

Taxon classificationFungiKickxellomycotaParakickxellomycetes

Tedersoo ord. nov.

E71D5B4E-964C-5D1E-9D0A-2B8934C66393

859087

##### Type family.

Parakickxellaceae Tedersoo.

##### Diagnosis.

Separation from other fungi based on diagnostic nucleotide signatures in SSU V8 (positions 1476–1495 in *S. cerevisiae* ccaagkcaacgagtytacaa; one mismatch allowed) and LSU D1 (positions 184–198 in type species and 180–194 in *S. cerevisiae* ggtataatttgcctg; one mismatch allowed). Forms a monophyletic, least inclusive clade in Parakickxellomycetes, covering sequences EUK1189320, EUK1189314, EUK1100806, EUK1189309, UDB014747, EUK1107625, EUK1105293, EUK1189310, EUK1700155, EUK1189325, EUK1189316, and EUK1189321 (Fig. 1).

##### Notes.

Recognized based on eDNA sequences only. Currently includes Parakickxellaceae (fam. nov.) and other potential family-level groups represented by sequences EUK1189320 (forest soil in Puerto Rico), EUK1189314 (forest soil in Dominica), EUK1100806 (agricultural soil in Great Britain), EUK1189309 (forest soil in Dominica), UDB014747 (woodland soil in Madagascar), EUK1107625 (unspecified soil in Tibet), EUK1105293 (forest soil in Puerto Rico), and EUK1189310 (forest soil in Dominica).

#### Parakickxellaceae

Taxon classificationFungiParakickxellomycetesParakickxellales

Tedersoo fam. nov.

4729255D-8B90-5E8A-A5F3-5D7EFD865685

859088

##### Type genus.

*Parakickxella* Tedersoo.

##### Diagnosis.

Separation from other fungi based on a diagnostic nucleotide signature in 5.8S (positions 117–126 in the type species and 120–129 in *S. cerevisiae* tggattactc; one mismatch allowed). Forms a monophyletic, least inclusive clade in Parakickxellales, covering sequences EUK1700155, EUK1189325, EUK1189316, and EUK1189321 (Fig. 1).

##### Notes.

Recognized based on eDNA sequences only. Includes *Parakickxella* (gen. nov.) and another potentially genus-level group represented by the sequence EUK1700155 (forest soil in Georgia).

#### Parakickxella

Taxon classificationFungiParakickxellalesParakickxellaceae

Tedersoo gen. nov.

F3551AD1-3938-5642-85D3-3636089EAA44

859089

##### Type species.

*Parakickxella borikenica* Tedersoo.

##### Diagnosis.

**Figure 47.**
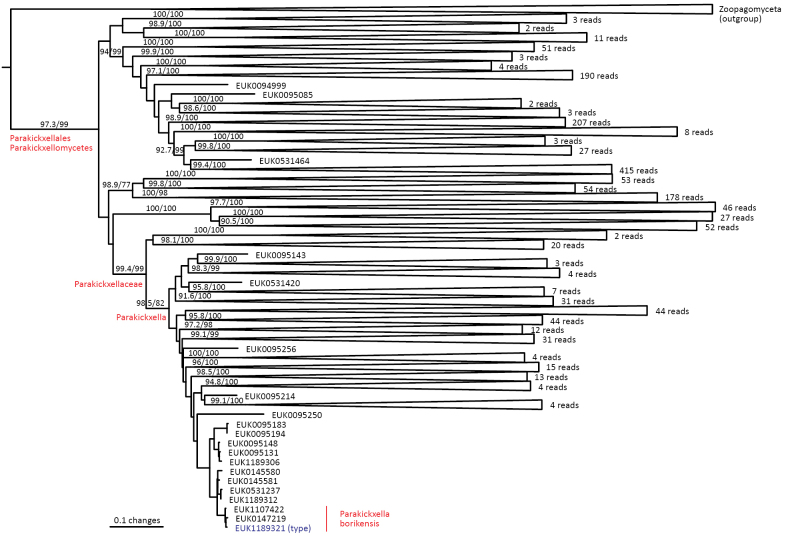
Maximum Likelihood SSU-ITS-LSU phylogram indicating the position of *Parakickxella borikenica* within Parakickxellomycetes, with ultra-rapid bootstrap values indicated (for higher-level classifications mainly). Other genus-level groups are collapsed. *Zoopagomyceta* spp. were used as an outgroup.

Separation from other fungi based on diagnostic nucleotide signatures in SSU V3 (positions 677–691 in *S. cerevisiae* gttccgcccggtctc; one mismatch allowed) and LSU D2 (positions 697–711 in the type species and 687–701 in *S. cerevisiae* cgacacgtcatggtg; one mismatch allowed). Forms a monophyletic, least inclusive clade in Parakickxellaceae, covering sequences EUK1189325, EUK1189316, and EUK1189321 (Figs 1, 47).

##### Notes.

Recognized based on eDNA sequences only. Comprises potentially about 150–170 species represented by sequences EUK1189325 (forest soil in Puerto Rico), EUK1189316 (forest soil in Dominica), EUK1189312 (forest soil in Dominica), EUK0483976 (forest soil in Guadeloupe), EUK1189319 (forest soil in Guadeloupe), EUK0530705 (forest soil in Costa Rica), EUK1189316 (forest soil in Dominica), and EUK1189306 (forest soil in Puerto Rico).

#### Parakickxella borikenica

Taxon classificationFungiParakickxellalesParakickxellaceae

Tedersoo sp. nov.

710F5E4C-87B4-5F42-97A6-51672DF63862

859090

##### Diagnosis.

**Figure 48.**
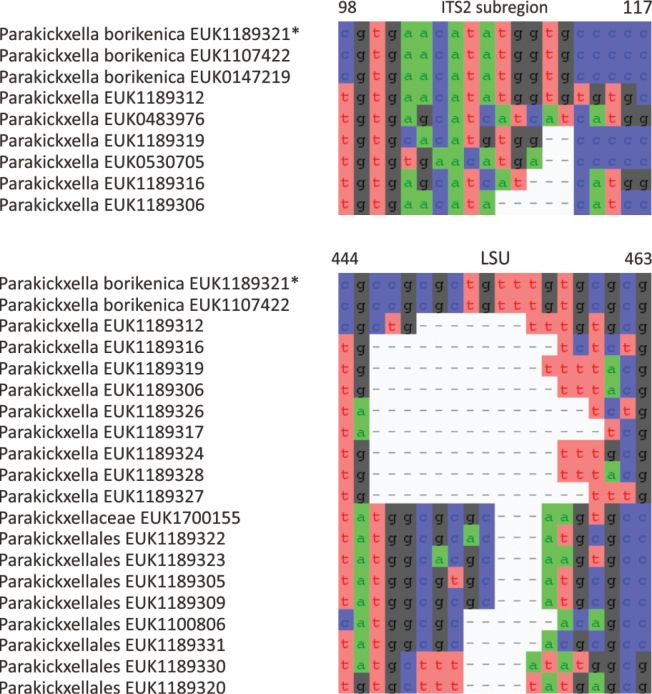
Diagnostic nucleotide sequences of *Parakickxella borikenica* relative to the closest related species in ITS2 and LSU. Numbers indicate positions in the legitype (marked with an asterisk).

Separation from other species of *Parakickxella* based on ITS2 (positions 98–117 cgtgaacatatggtgccccc; one mismatch allowed) and LSU D2 (positions 444–463 in the type species and 436–455 in *S. cerevisiae* cgccgcgctgtttgtgcgcg; one mismatch allowed) as indicated in Fig. 48. Intraspecific difference up to 1.4% in ITS2 and up to 0.5% in LSU. Interspecific distance at least 15.0% in ITS2.

##### Type.

Vouchered soil sample TUE000315 (**holotype**); eDNA sequence EUK1189321 = OZ253810 (**legitype**); eDNA sample TUE100315 (**nucleotype**); GSMc plot S045, tropical rainforest soil in El Yunque, Puerto Rico, 18.3167, –65.8167°E.

##### Description.

Other sequences: EUK1107422 (tropical rainforest soil in El Yunque, Puerto Rico, 18.29, –65.78); EUK0147219 (GSMc plot G5034, tropical rainforest soil in Los Pinos, Puerto Rico, 18.1268, –66.0724°E); and GlobalFungi accession dfbf3c41964a835260bb3a9afcdaf69a (tropical rainforest soil in Luquillo, Puerto Rico, 18.3, –65.8).

##### Etymology.

*Parakickxella* (Latin) refers to a taxon distant from *Kickxella*, and *Boriken* (Taino) refers to the native name of Puerto Rico, where the type material and other specimens originate.

##### Notes.

Found exclusively in soil in Puerto Rico, which is confirmed by an additional GlobalFungi record.

#### Aldinomycota

Taxon classificationFungiFungiMucoromycota

Tedersoo phyl. nov.

752126C0-938B-50C5-9E79-5234344031E8

859091

##### Type class.

Aldinomycetes Tedersoo.

##### Diagnosis.

Distinguishable from other fungi based on diagnostic nucleotide signatures in SSU V4 (positions 732–746 gattcaggaccttca in *S. cerevisiae*; no mismatch allowed), SSU V7 (positions 1346–1355 gttgttggtc in *S. cerevisiae*; no mismatch allowed), and LSU D3 (positions 912–926 in the type species and 771–785 in *S. cerevisiae*: ggttttgagaaaaag; one mismatch allowed). Forms a monophyletic, least inclusive clade in fungi, covering sequences EUK0320466, EUK0529888, EUK1205365, EUK1124394, EUK0529884, EUK1111390, EUK0529911, and EUK0320468 (Fig. 1).

##### Notes.

Recognized based on eDNA sequences only. Encoded as clade GS45 in EUKARYOME v1.9. Comprises potentially 65–70 species. Currently harbors Aldinomycetes (class. nov.). Detected in soil (98.2% out of 113 records) and sediments (1.8%) in tundra to wet tropical biomes across all continents except Antarctica.

#### Aldinomycetes

Taxon classificationFungiMucoromycotaAldinomycota

Tedersoo class. nov.

56AEDF14-91A8-5978-9F6E-A2E9A32479A3

859092

##### Type order.

Aldinomycetales Tedersoo.

##### Diagnosis.

Distinguishable from other fungi based on diagnostic nucleotide signatures in SSU V4 (positions 732–746 gattcaggaccttca in *S. cerevisiae*; no mismatch allowed), SSU V7 (positions 1346–1355 gttgttggtc in *S. cerevisiae*; no mismatch allowed), and LSU D3 (positions 912–926 in the type species and 771–785 in *S. cerevisiae*: ggttttgagaaaaag; one mismatch allowed). Forms a monophyletic, least inclusive clade in Aldinomycota, covering sequences EUK0320466, EUK0529888, EUK1205365, EUK1124394, EUK0529884, EUK1111390, EUK0529911, and EUK0320468 (Fig. 1).

##### Notes.

Recognized based on eDNA sequences only. Currently harbors Aldinomycetales (ord. nov.).

#### Aldinomycetales

Taxon classificationFungiAldinomycotaAldinomycetes

Tedersoo ord. nov.

0B22B2F5-324D-5725-B3E5-210289CBDDEB

959093

##### Type family.

Aldinomycetaceae Tedersoo.

##### Diagnosis.

Distinguishable from other fungi based on diagnostic nucleotide signatures in SSU V4 (positions 732–746 gattcaggaccttca in *S. cerevisiae*; no mismatch allowed), SSU V7 (positions 1346–1355 gttgttggtc in *S. cerevisiae*; no mismatch allowed), and LSU D3 (positions 912–926 in the type species and 771–785 in *S. cerevisiae*: ggttttgagaaaaag; one mismatch allowed). Forms a monophyletic, least inclusive clade in Aldinomycetes, covering sequences EUK0320466, EUK0529888, EUK1205365, EUK1124394, EUK0529884, EUK1111390, EUK0529911, and EUK0320468 (Fig. 1).

##### Notes.

Recognized based on eDNA sequences only. Currently includes Aldinomycetaceae (fam. nov.) and other potentially family-level groups represented by sequences EUK1111390 (forest soil in Sweden), EUK0320466 (river sediment in Spain), EUK0529888 (orchard soil in Estonia), and EUK0320468 (river sediment in Spain).

#### Aldinomycetaceae

Taxon classificationFungiAldinomycetesAldinomycetales

Tedersoo fam. nov.

FDED5FD6-E64C-55A6-BD6C-968396FA849E

859094

##### Type genus.

*Aldinomyces* Tedersoo.

##### Diagnosis.

Distinguishable from other fungi based on diagnostic nucleotide signatures in SSU V9 (positions 1686–1695 tagcgatagg in *S. cerevisiae*; no mismatch allowed) and ITS2 (positions 125–137 gcaacatartaat in type species; one mismatch allowed). Forms a monophyletic, least inclusive clade in Aldinomycetales, covering sequences EUK1205365, EUK1124394, EUK0529911, and EUK0529884 (Fig. 1).

##### Notes.

Recognized based on eDNA sequences only. Includes *Aldinomyces* (gen. nov.) and potentially genus-level taxa represented by sequences JX898614 (cave debris in NY, USA) and EUK0529884 (grassland soil in Estonia).

#### Aldinomyces

Taxon classificationFungiAldinomycetalesAldinomycetaceae

Tedersoo gen. nov.

C00F1B46-B22C-5C6F-B3C1-C68E3D941521

859095

##### Type species.

*Aldinomyces tarquinii* Tedersoo.

##### Diagnosis.

**Figure 49.**
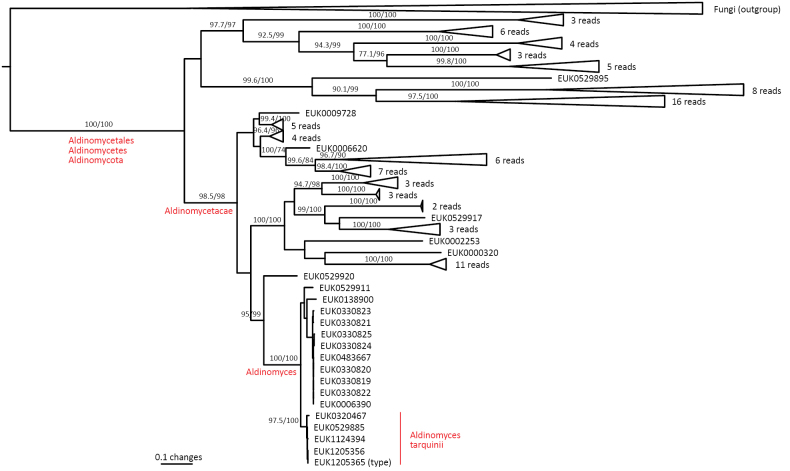
Maximum Likelihood SSU-ITS-LSU phylogram indicating the position of *Aldinomyces tarquinii* within Aldinomycota, with ultra-rapid bootstrap values indicated (for higher-level classifications mainly). Other genus-level groups are collapsed. Members of various fungal phyla were used as an outgroup.

Distinguishable from other fungi based on a diagnostic nucleotide signature in ITS2 (positions 139–158 gcggatttcgaaagatttct in type species; one mismatch allowed). Forms a monophyletic, least inclusive clade in Aldinomycetaceae, covering sequences EUK1205365, EUK1124394, and EUK0529911 (Figs 1, 49).

##### Notes.

Recognized based on eDNA sequences only. Comprises about four potential species represented by sequences EUK0483667 (forest soil in Argentina), EUK0138900 (forest soil in Norway), and EUK0529911 (woodland soil in Estonia).

#### Aldinomyces tarquinii

Taxon classificationFungiAldinomycetalesAldinomycetaceae

Tedersoo sp. nov.

C3C7C641-FE2D-52C9-AC85-EECBA36E96AF

859096

##### Diagnosis.

**Figure 50.**
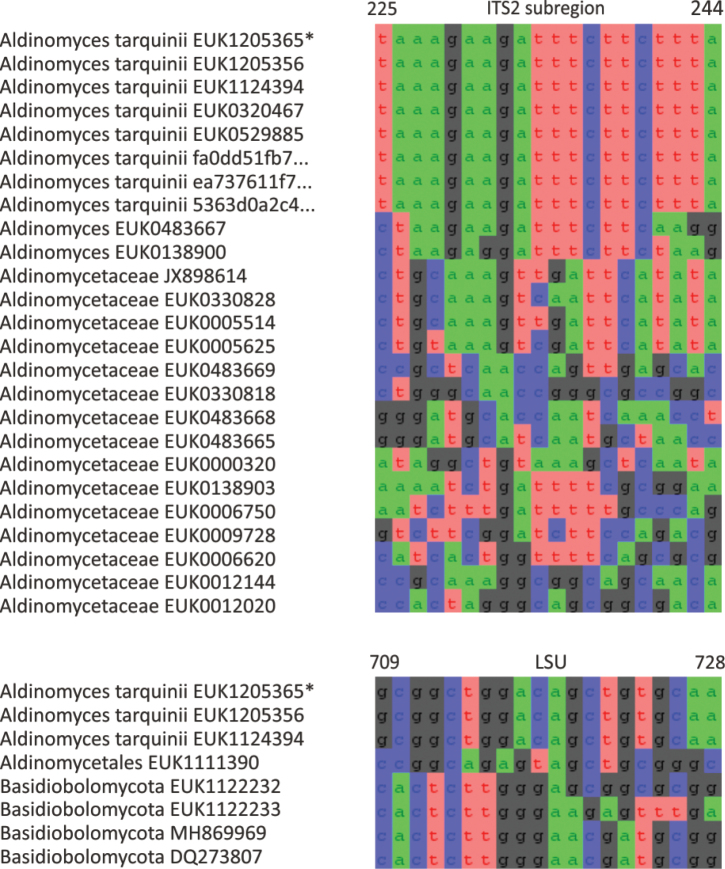
Diagnostic nucleotide sequences of *Aldinomyces tarquinii* relative to the closest related species in ITS2 and LSU. Numbers indicate positions in the legitype (marked with an asterisk). GlobalFungi abbreviations, fa0dd51fb7, fa0dd51fb77b34cf5b5659d5f4d674c1; ea737611f7, ea737611f71505778a4409d28eec463d; and 5363d0a2c4, 5363d0a2c4815da4d7fb36f7f94d6c6e.

Separation from other species of *Aldinomyces* based on ITS2 (positions 225–244 taaagaagatttcttcttta; two mismatches allowed) and LSU D2 (positions 709–728 gcggctggacagctgtgcaa; one mismatch allowed) as indicated in Fig. 50. Intraspecific variation up to 1.1% in ITS2 and up to 0.4% in LSU. Interspecific distance at least 3.9% in ITS2.

##### Type.

Vouchered soil sample TUE002655 (**holotype**); eDNA sequence EUK1205365 = OZ253811 (**legitype**); eDNA sample TUE102655 (**nucleotype**); GSMc plot S1183, mixed forest in Aldino, Italy, 46.4072°N, 11.4964°E.

##### Description.

Other sequences: EUK1205356 (type locality); EUK1124394 (GSMc plot G5912, temperate grassland soil in Rahinge, Estonia, 58.3804°N, 26.6289°E); EUK0320467 (FunAqua sediment sample W0315s in Cottonwood Lake, ND, USA, 47.1, –99.09); EUK0529885 (GSMc plot G2838X; tundra soil in Kvaenangsfjellet, Norway, 69.8972°N, 21.5778°E); and GlobalFungi accessions fa0dd51fb77b34cf5b5659d5f4d674c1 (forest soil in Yunnan, China, 27.12°N, 100.17°E); ea737611f71505778a4409d28eec463d (woodland soil in MT, USA, 45.3982, –110.704); and 5363d0a2c4815da4d7fb36f7f94d6c6e (forest soil in Austria, 47.36°N, 15.05°E).

##### Etymology.

*Aldino* (Italian) refers to the type locality, and *Tarquin* (English) refers to Tarquin Netherway, who collected the material from the type locality.

##### Notes.

Found in three soil samples and one sediment sample in the Northern Hemisphere. GlobalFungi records (n = 12) confirm the distribution in soils of the Holarctic realm.

#### Borikeniomycota

Taxon classificationFungiFungiMucoromycota

Tedersoo phyl. nov.

95C66670-7222-579A-8600-1BA54F951155

859097

##### Type class.

Borikeniomycetes Tedersoo.

##### Diagnosis.

Distinguishable from other fungi based on a diagnostic nucleotide signature in LSU D3 (positions 956–967 in type species and 761–772 in *S. cerevisiae* tcaatttattga OR ggaatttattcc; two mismatches allowed). Forms a monophyletic, least inclusive clade in fungi, covering sequences EUK1105319, EUK1189257, EUK0530094, EUK0530090, EUK1189254, EUK1189255, and EUK1189256 (Fig. 1).

##### Notes.

Recognized based on eDNA sequences only. Encoded as clade GS47 in EUKARYOME v1.9. Currently harbors Borikeniomycetes (class. nov.). Comprises potentially 15–16 species. Detected exclusively from soil (all 48 records) in warm temperate to wet tropical biomes across all continents except Antarctica. A single record is from tundra soil (EUK0530093; Russian Federation). The group is mainly distributed in the Neotropics (72.9% records), especially the Antilles and Colombia.

#### Borikeniomycetes

Taxon classificationFungiMucoromycotaBorikeniomycota

Tedersoo class. nov.

9A4688DE-4086-55E5-ADEE-2A888180984B

859098

##### Type order.

Borikeniales Tedersoo.

##### Diagnosis.

Distinguishable from other fungi based on a diagnostic nucleotide signature in LSU D3 (positions 956–967 in type species and 761–772 in *S. cerevisiae* tcaatttattga OR ggaatttattcc; two mismatches allowed). Forms a monophyletic, least inclusive clade in Borikeniomycota, covering sequences EUK1105319, EUK1189257, EUK0530094, EUK0530090, EUK1189254, EUK1189255, and EUK1189256 (Fig. 1).

##### Notes.

Recognized based on eDNA sequences only. Currently harbors Borikeniales (ord. nov.).

#### Borikeniales

Taxon classificationFungiBorikeniomycotaBorikeniomycetes

Tedersoo ord. nov.

55DBFC68-82BA-52F6-89EC-30B79A436A7C

859099

##### Type family.

Borikeniaceae Tedersoo.

##### Diagnosis.

Distinguishable from other fungi based on a diagnostic nucleotide signature in LSU D3 (positions 956–967 in type species and 761–772 in *S. cerevisiae* tcaatttattga OR ggaatttattcc; no mismatch allowed). Forms a monophyletic, least inclusive clade in Borikeniomycetes, covering sequences EUK1105319, EUK1189257, EUK0530094, EUK0530090, EUK1189254, EUK1189255, and EUK1189256 (Fig. 1).

##### Notes.

Recognized based on eDNA sequences only. Currently includes Borikeniaceae (fam. nov.) and potentially a family-level group represented by sequences EUK1189257 (forest soil in Dominica) and EUK1105319 (forest soil in Puerto Rico).

#### Borikeniaceae

Taxon classificationFungiBorikeniomycetesBorikeniales

Tedersoo fam. nov.

C3FDB6E5-0AAA-531E-ABFA-738C8E522988

859100

##### Type genus.

*Borikenia* Tedersoo.

##### Diagnosis.

Distinguishable from other fungi based on diagnostic nucleotide signatures in LSU D3 (positions 956–967 in type species and 761–772 in *S. cerevisiae* tcaatttattga; no mismatch allowed) and 5.8S (positions 130–142 in type species and 132–144 in *S. cerevisiae* aagagtatttctg; one mismatch allowed). Forms a monophyletic, least inclusive clade in Borikeniales, covering sequences EUK1189254, EUK0530094, EUK0530090, EUK1189255, and EUK1189256 (Fig. 1).

##### Notes.

Recognized based on eDNA sequences only. Includes the genus *Borikenia* (gen. nov.) and another potential genus-level group represented by sequences EUK1189255 (forest soil in the British Virgin Islands) and EUK1189256 (forest soil in Dominica).

#### Borikenia

Taxon classificationFungiBorikenialesBorikeniaceae

Tedersoo gen. nov.

97A81F34-F622-5DFF-985A-49B505AE76A4

859101

##### Type species.

*Borikenia urbinae* Tedersoo.

##### Diagnosis.

**Figure 51.**
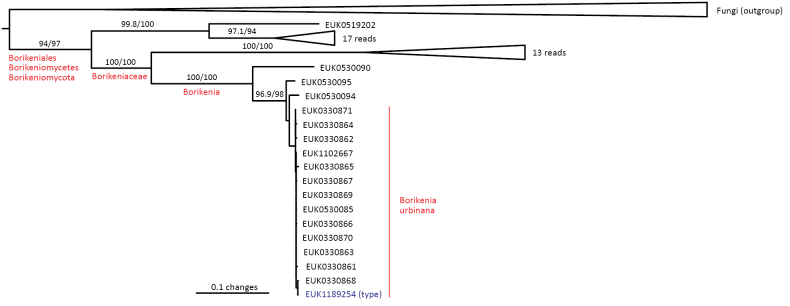
Maximum Likelihood SSU-ITS-LSU phylogram indicating the position of *Borikenia urbinae* within Borikeniomycota, with ultra-rapid bootstrap values indicated (for higher-level classifications mainly). Other genus-level groups are collapsed. Members of various fungal phyla were used as an outgroup.

Distinguishable from other fungi based on diagnostic nucleotide signatures in SSU V9 (positions 1684–1703 in *S. cerevisiae* tgcggtccacatgttggcaa; one mismatch allowed) and ITS2 (positions 26–45 in type species ttggtggacttggtcgttca; two mismatches allowed). Forms a monophyletic, least inclusive clade in Borikeniaceae, covering sequences EUK1189254, EUK0530094, and EUK0530090 (Figs 1, 51).

##### Notes.

Recognized based on eDNA sequences only. Comprises four potential species represented by sequences EUK0530090 (forest soil in India), EUK0530094 (forest soil in Colombia), and EUK0530095 (forest soil in Costa Rica).

#### Borikenia urbinana

Taxon classificationFungiBorikenialesBorikeniaceae

Tedersoo sp. nov.

41E2A976-CEEE-5A6D-B75B-CFAE71303BE2

859102

##### Diagnosis.

**Figure 52.**
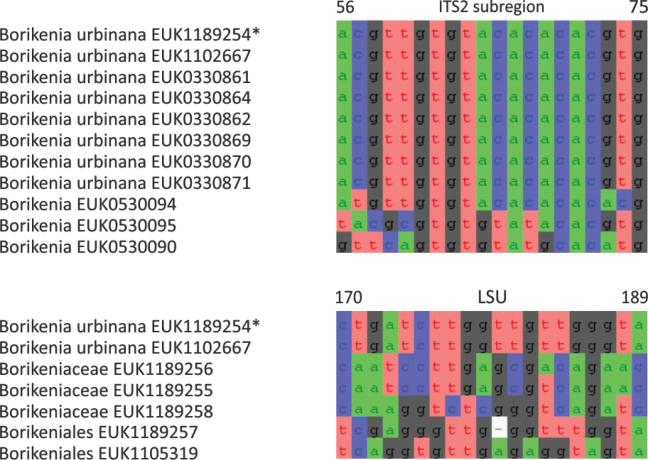
Diagnostic nucleotide sequences of *Borikenia urbinae* relative to the closest related species in ITS2 and LSU. Numbers indicate positions in the legitype (marked with an asterisk).

Separation from other species of *Borikenia* based on ITS2 (positions 56–75 acgttgtgtacacacacgtg; one mismatch allowed) and LSU (positions 170–189 ctgatcttggttgttgggta; one mismatch allowed) as indicated in Fig. 52. Intraspecific variation up to 2.3% in ITS2 and up to 0.4% in LSU. Interspecific distance at least 6.1% in ITS2.

##### Type.

Vouchered soil sample TUE002010 (**holotype**); eDNA sequence EUK1189254 = OZ253812 (**legitype**); eDNA sample TUE102010 (**nucleotype**); GSMc plot G5033, tropical rainforest soil in Luquillo, Puerto Rico, 18.3146, –65.747.

##### Description.

Other sequences: EUK1102667 (tropical rainforest soil in El Yunque, Puerto Rico, 18.29, –65.78); EUK0330861 (GSMc plot AV207, tropical rainforest soil in Puerto Santander, Colombia, –0.6161, –72.401); EUK0330863 (GSMc plot S1227, *Eucalyptus* plantation soil in Ayapel, Colombia, 8.27, –75.2); EUK0330864 (GSMc plot S1026, tropical rainforest soil in Matouta, Reunion, France, –21.3522°N, 55.7059°E); EUK0330862 (GSMc plot S003, *Uapaca* tropical forest soil in Manangotry, Madagascar, –24.745, 46.852); EUK0330869 (GSMc plot S048, tropical rainforest soil in El Yunque, Puerto Rico, 18.3167, –65.8167°E); EUK0330870 (GSMc plot JYK035, *Eucalyptus* plantation soil in Rivercess, Liberia, 5.7282, –9.629); and EUK0330871 (GSMc plot S1267, tropical rainforest soil in Khong Ngam, Thailand, 20.2433°N, 100.0981°E).

##### Etymology.

*>Boriken* (Taino) refers to Puerto Rico, where the type material originates, and *Urbina* (Spanish) refers to Hector Urbina, who was the first to collect material from this species (EUK1102667).

##### Notes.

Found in tropical grassland and forest soils in America, Africa, and Asia (11 localities). GlobalFungi reveals an additional record from subtropical forest soil in China.

#### Mirabilomycota

Taxon classificationFungiFungiMucoromycota

Tedersoo & R.H. Nilsson phyl. nov.

E44AF40A-0E80-5AEC-979E-81E0192B894D

859103

##### Type class.

Mirabilomycetes Tedersoo & R.H. Nilsson.

##### Diagnosis.

Forms a monophyletic, least inclusive clade in fungi, covering sequences EUK1101676, EUK1107181, EUK1103059, EUK1100023, EUK1103883, EUK1102753, EUK1100742, EUK1204083, EUK1200757, EUK1201873, EUK1211619, EUK1200676, EUK1201256, EUK1203033, EUK1201246, EUK1201583, EUK1107008, EUK1110728, EUK1109741, EUK1201657, EUK1109988, EUK1108787, and EUK1115028 (Fig. 1).

##### Notes.

Recognized based on eDNA sequences only. Encoded as clade GS41 in EUKARYOME v1.9. There are no diagnostic nucleotide signatures to distinguish them from other fungi due to rapid rRNA gene evolution in certain class- and order-level groups. Currently harbors Mirabilomycetes (class. nov) and potentially class-level groups represented by sequences EUK1101676 (forest soil in Puerto Rico), EUK1107181 (forest soil in Puerto Rico), EUK1103059 (forest soil in Puerto Rico), EUK1100023 (forest soil in Sweden), EUK1103883 (forest soil in Puerto Rico), and EUK1102753 (forest soil in Puerto Rico). Comprises potentially 1500–1800 species. Detected in soil (99.9% out of 6193 records) and sediments (0.1%) in tundra to wet tropical biomes across all continents except Antarctica.

#### Mirabilomycetes

Taxon classificationFungiMucoromycotaMirabilomycota

Tedersoo & R.H. Nilsson class. nov.

47A202E2-D655-5092-8B85-7154A032CC2A

859104

##### Type order.

Mirabilomycetales Tedersoo & R.H. Nilsson.

##### Diagnosis.

Distinguishable from other fungi based on a diagnostic nucleotide signature in ITS2 (positions 102–109 cttgaaat in the type species; one mismatch allowed). Forms a monophyletic, least inclusive clade in Mirabilomycota, covering sequences EUK1100742, EUK1204083, EUK1200757, EUK1201873, EUK1211619, EUK1200676, EUK1201256, EUK1203033, EUK1201246, EUK1201583, EUK1107008, EUK1110728, EUK1109741, EUK1201657, EUK1109988, EUK1108787, and EUK1115028 (Fig. 1).

##### Notes.

Recognized based on eDNA sequences only. Currently harbors Mirabilomycetales and potentially order-level groups represented by sequences EUK1107008 (forest soil in Puerto Rico), EUK1110728 (forest soil in Sweden), EUK1109741 (forest soil in Puerto Rico), EUK1201657 (forest soil in Estonia), EUK1109988 (forest soil in Puerto Rico), EUK1108787 (cropland soil in Great Britain), and EUK1115028 (unspecified soil in Tibet).

#### Mirabilomycetales

Taxon classificationFungiMirabilomycotaMirabilomycetes

Tedersoo & R.H. Nilsson

DF6AA243-BEE9-5033-B233-F98BE284745B

859105

##### Type family.

Mirabilomycetaceae Tedersoo & R.H. Nilsson.

##### Diagnosis.

Distinguishable from other fungi based on a diagnostic nucleotide signature in the LSU 5’ end (positions 1–11 in the type species and *S. cerevisiae* tcattctcaac or cggatctcaaa; one mismatch allowed). Forms a monophyletic, least inclusive clade in Mirabilomycetes, covering sequences EUK1100742, EUK1204083, EUK1200757, EUK1201873, EUK1211619, EUK1200676, EUK1201256, EUK1203033, EUK1201246, and EUK1201583 (Fig. 1).

##### Notes.

Recognized based on eDNA sequences only. Currently includes Mirabilomycetaceae (fam. nov.) and other potentially family-level groups represented by sequences EUK1100742 (unspecified soil in Tibet), EUK1204083 (tundra soil in Norway), EUK1200757 (grassland soil in Italy), EUK1201873 (forest soil in Estonia), and EUK1211619 (grassland soil in Italy).

#### Mirabilomycetaceae

Taxon classificationFungiMirabilomycetesMirabilomycetales

Tedersoo & R.H. Nilsson fam. nov.

48676594-1E59-5DEA-A5C8-315E1C70D389

859106

##### Type genus.

*Mirabilomyces* Tedersoo & R.H. Nilsson.

##### Diagnosis.

Distinguishable from other fungi based on diagnostic nucleotide signatures in the LSU 5’ end (positions 1–11 in the type species and *S. cerevisiae* tcattctcaac; one mismatch allowed) and ITS2 (positions 196–208 in type species aacaatgacttga; one mismatch allowed). Forms a monophyletic, least inclusive clade in Mirabilomycetales, covering sequences EUK1200676, EUK1201256, EUK1203033, EUK1201246, EUK1201583, EUK1201728, and EUK1124366 (Fig. 1).

##### Notes.

Recognized based on eDNA sequences only. Includes *Mirabilomyces* (gen. nov.) and other potentially genus-level groups represented by sequences EUK1201256 (grassland soil in Switzerland), EUK1203033 (forest soil in the Canary Islands), EUK1201246 (forest soil in Estonia), and EUK1201583 (grassland soil in Norway).

#### Mirabilomyces

Taxon classificationFungiMirabilomycetalesMirabilomycetaceae

Tedersoo & R.H. Nilsson gen. nov.

B3FE3A94-FDA1-5C71-A66A-46D7D33132EC

859107

##### Type species.

*Mirabilomyces abrukanus* Tedersoo & R.H. Nilsson.

##### Diagnosis.

**Figure 53.**
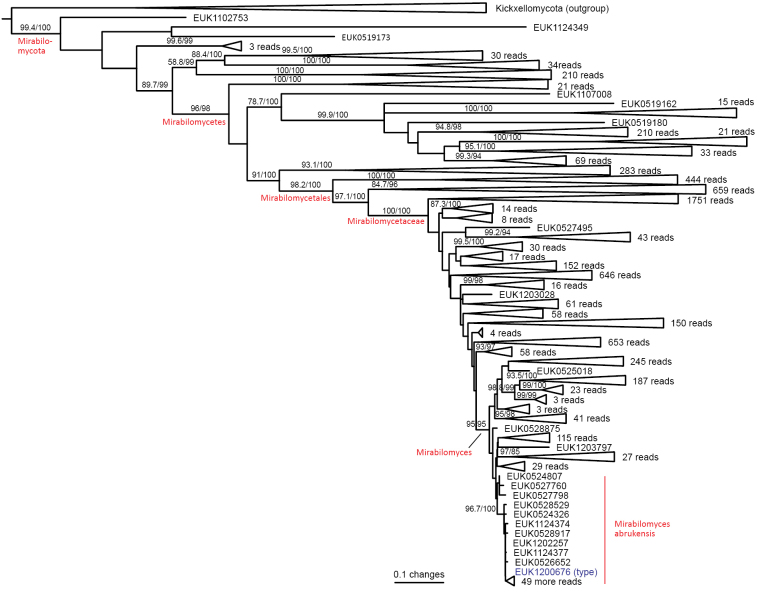
Maximum Likelihood SSU-ITS-LSU phylogram indicating the position of *Mirabilomyces abrukanus* within Mirabilomycota, with ultra-rapid bootstrap values indicated (for higher-level classifications mainly). Other genus-level groups are collapsed. Kickxellomycota spp. were used as an outgroup.

Distinguishable from other fungi based on a diagnostic nucleotide signature in ITS2 (positions 205–214 cttgattagt in type species; one mismatch allowed). Forms a monophyletic, least inclusive clade in Mirabilomycetaceae, covering sequences EUK1200676 and EUK1124366 (Figs 1, 53).

##### Notes.

Recognized based on eDNA sequences only. Comprises 170–200 potential species represented by sequences EUK1201728 (forest soil in Estonia), EUK1124366 (grassland soil in Estonia), EUK1101799 (forest soil in Puerto Rico), EUK1101831 (forest soil in Puerto Rico), EUK1101652 (forest soil in Puerto Rico), EUK1101776 (forest soil in Puerto Rico), and OU943050 (grassland soil in Sweden).

#### Mirabilomyces abrukanus

Taxon classificationFungiMirabilomycetalesMirabilomycetaceae

Tedersoo & R.H. Nilsson sp. nov.

0B9123C4-B23D-5826-B698-1673656AA253

859108

##### Diagnosis.

**Figure 54.**
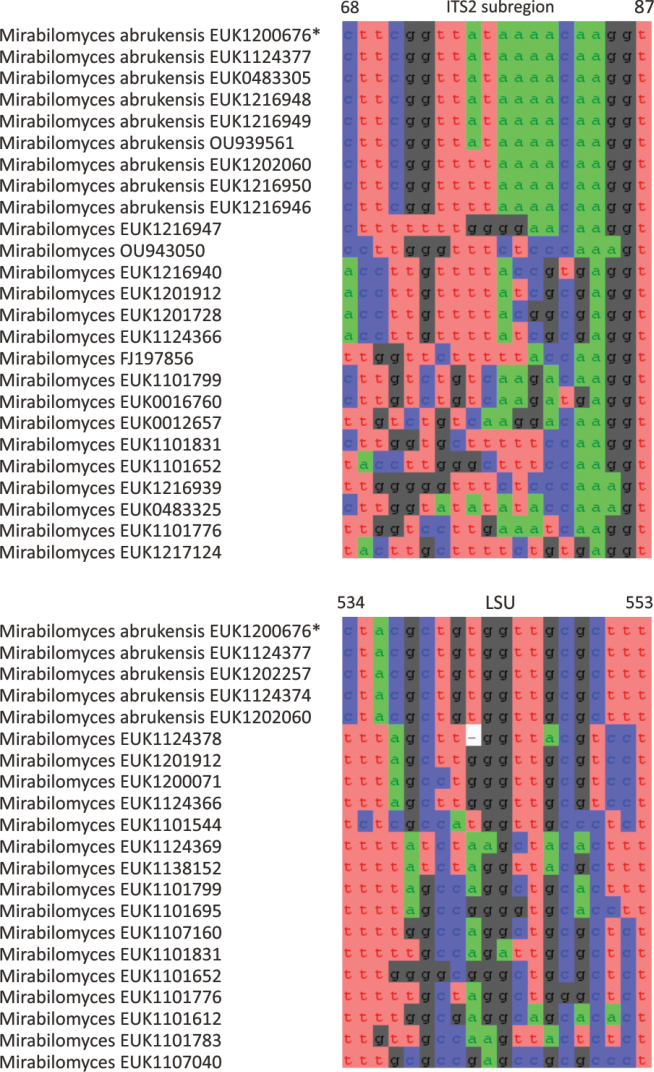
Diagnostic nucleotide sequences of *Mirabilomyces abrukanus* relative to the closest related species in ITS2 and LSU. Numbers indicate positions in the legitype (marked with an asterisk).

Separation from other species of *Mirabilomyces* based on ITS2 (positions 68–87 cttcggttwtaaaacaaggt; two mismatches allowed) and LSU (positions 534–553 ctacgctgtggttgcgcttt; one mismatch allowed) as indicated in Fig. 54. Intraspecific variation up to 11.4% in ITS2 due to length polymorphism in multiple mono- and dinucleotide repeats and up to 1.0% in LSU. Interspecific distance > 20% in ITS2 and at least 6.0% in LSU.

##### Type.

Vouchered soil sample TUE000464 (**holotype**); eDNA sequence EUK1200676 = OZ253813 (**legitype**); eDNA sample TUE100464 (**nucleotype**); GSMc plot S208, *Tilia cordata* temperate forest soil in Abruka, Estonia, 58.1568°N, 22.5004°E.

##### Description.

Other sequences: EUK0483305 (temperate broadleaf forest soil in Arcais, France, 46.3038, –0.6844°E); EUK1124377 (GSMc plot G5235, *Larix decidua* plantation soil in Rõõmu, Estonia, 58.3835°N, 26.7742°E); EUK1216948 (GSMc plot G4777, flooded grassland soil in Suur-Pakri Härs-hämani, Estonia, 59.3310°N, 23.9272°E); EUK1216949 (GSMc plot G4679, *Salix triandra* wetland soil in Prangli Rivimaa, Estonia, 59.6150°N, 24.9871°E); OU939561 (grassland soil in Kungsängen, Sweden, 59.837°N, 17.661°E); EUK1202060 (GSMc plot G4747, *Prunus-Rhamnus-Euonymus* forest soil in Tsirgumäe, Estonia, 57.5942°N, 26.3241°E); EUK1216950 (GSMc plot G4742, *Fraxinus-Ulmus* forest soil in Lüütre, Estonia, 58.1444°N, 25.2628°E); and EUK1216946 (GSMc plot S1366, temperate grassland soil in Innhavet, Norway, 67.9676°N, 15.9277°E).

##### Etymology.

*Mirabilis* (Latin) refers to the remarkable and astonishing finding of a large, unrecognized fungal lineage, and *Abruka* (Estonian) refers to the type locality of the species.

##### Notes.

Found in grassland and forest soils in North and Central Europe (n = 57 records), with single records from Asia, North America, and Africa. GlobalFungi reveals no additional information.

#### Nematovomycota

Taxon classificationFungiFungiMucoromycota

Tedersoo & Esmaeilzadeh-Salestani phyl. nov.

31C68CB9-55F6-5C2F-86CE-AC8A33D3AA3D

859109

##### Type class.

Nematovomycetes Tedersoo & Esmaeilzadeh-Salestani.

##### Diagnosis.

Distinguishable from other fungi based on a diagnostic nucleotide signature in the LSU 5´ end (positions 5–14 in the type species and *S. cerevisiae* cctgaawtta; one mismatch allowed). Forms a monophyletic, least inclusive clade in fungi, covering sequences EUK1124405, EUK1137897, EUK1138000, EUK1105583, EUK1217236, EU162639, AB971078, OL869110, EUK1217234, EUK1137920, EUK1124400, AB971072, EUK1106088, OQ702947, GQ330624, OQ702883, JN054659, JN054675, OQ702805, EUK1100016, EUK1217270, and EUK1124397 (Fig. 1).

##### Notes.

Encoded as clade GS46 in EUKARYOME v1.9. Currently harbors Nematovomycetes (class. nov.) and potentially class-level groups represented by sequences EUK1124405 (soil in Estonia), EUK1137897 (lake sediment in Germany), EUK1138000 (lake sediment in Germany), EUK1105583 (marine water near Sweden), EUK1217236 (lake sediment in Serbia), EU162639 (lake water in France), AB971078 (lake water in Japan), OL869110 (lake water in Germany), and EUK1217234 (brackish water sediment in Estonia). Nematovomycota comprises potentially 240–260 species. Detected in soil (49.3% out of 458 records), sediments (26.4%), and water (23.6%). Seto et al. (2023) revealed connections to nematode eggs (OQ702805), rotifer eggs (OQ702883), or rotifers (OQ702947), suggesting parasitism on microfauna in contrasting environments. Recorded from high arctic to wet tropical biomes across all continents, including Antarctica.

#### Nematovomycetes

Taxon classificationFungiMucoromycotaNematovomycota

Tedersoo & Esmaeilzadeh-Salestani class. nov.

3E348D7C-A3D0-57D6-B529-35DB51798B69

859110

##### Type order.

Nematovomycetales Tedersoo & Esmaeilzadeh-Salestani.

##### Diagnosis.

Distinguishable from other fungi based on a diagnostic nucleotide signature in 5.8S (positions 127–146 in *N. soinasteënsis* and *S. cerevisiae*: atccggyaggtatacctatt or gcctgcaggtatacctattt or acgtgcaagtatacctattt or atccaaagagtatacttgtt; one mismatch allowed). Forms a monophyletic, least inclusive clade in Nematovomycota, covering sequences EUK1217270, EUK1137920, EUK1124402, EUK1124400, AB971072, EUK1106088, OQ702947, GQ330624, OQ702883, JN054659, JN054675, OQ702805, EUK1100016, EUK1107129, EUK1102228, EUK1204135, EUK1124398, EUK1124395, EUK1124396, EUK1200775, and EUK1124397 (Fig. 1).

##### Notes.

Nematovomycetes currently harbors Nematovomycetales (ord. nov.) and a potentially order-level group represented by the sequence EUK1217270 (lake sediment in Portugal).

#### Nematovomycetales

Taxon classificationFungiNematovomycotaNematovomycetes

Tedersoo & Esmaeilzadeh-Salestani ord. nov.

986895C5-F6DA-59F7-B958-013848DF120C

859111

##### Type family.

Nematovomycetaceae Tedersoo & Esmaeilzadeh-Salestani.

##### Diagnosis.

Distinguishable from other fungi based on diagnostic nucleotide signatures in 5.8S (positions 127–146 in *N. soinasteënsis* and *S. cerevisiae* atccggyaggtatacctatt or gcctgcaggtatacctattt or acgtgcaagtatacctattt; one mismatch allowed) and in LSU D3 (positions 905–919 in *N. soinasteënsis* and 748–762 in *S. cerevisiae*: acccgatcctagctc; two mismatches allowed). Forms a monophyletic, least inclusive clade in Nematovomycetes, covering sequences EUK1137920, EUK1124402, EUK1124400, AB971072, EUK1106088, OQ702947, GQ330624, OQ702883, JN054659, JN054675, OQ702805, EUK1100016, EUK1107129, EUK1102228, EUK1204135, EUK1124398, EUK1124395, EUK1124396, EUK1200775, and EUK1124397 (Fig. 1).

##### Notes.

Currently includes Nematovomycetaceae and another potentially family-level group represented by sequences EUK1137920 (forest soil in Estonia), EUK1202819 (grassland soil in Estonia), EUK1113339 (lake water in Sweden), and EUK1124400 (lake sediment in Estonia).

#### Nematovomycetaceae

Taxon classificationFungiNematovomycetesNematovomycetales

Tedersoo & Esmaeilzadeh-Salestani fam. nov.

3AC66B43-6BE4-54D7-849B-0E398F1BAB74

859112

##### Type genus.

*Nematovomyces* Tedersoo & Esmaeilzadeh-Salestani.

##### Diagnosis.

Distinguishable from other fungi based on a diagnostic nucleotide signature in ITS2 (positions 68–77 aacaatgtct or atcaatggtt in *N. soinasteënsis*; one mismatch allowed). Forms a monophyletic, least inclusive clade in Nematovomycetales, covering sequences AB971072, EUK1106088, OQ702947, GQ330624, OQ702883, JN054659, JN054675, OQ702805, EUK1100016, EUK1107129, EUK1102228, EUK1204135, EUK1124398, EUK1124395, EUK1124396, and EUK1124397 (Fig. 1).

##### Notes.

Includes *Nematovomyces* (gen. nov.) and another potentially genus-level group represented by sequences AB971072 (lake water in Japan) and EUK1106088 (peatland soil in Sweden).

#### Nematovomyces

Taxon classificationFungiNematovomycetalesNematovomycetaceae

Tedersoo & Esmaeilzadeh-Salestani gen. nov.

ED79EBD6-4927-5F33-A626-0BE13B115E36

859113

##### Type species.

*Nematovomyces vermicola* (G.L. Barron & Szuarto) Tedersoo & Esmaeilzadeh-Salestani.

##### Diagnosis.

**Figure 55.**
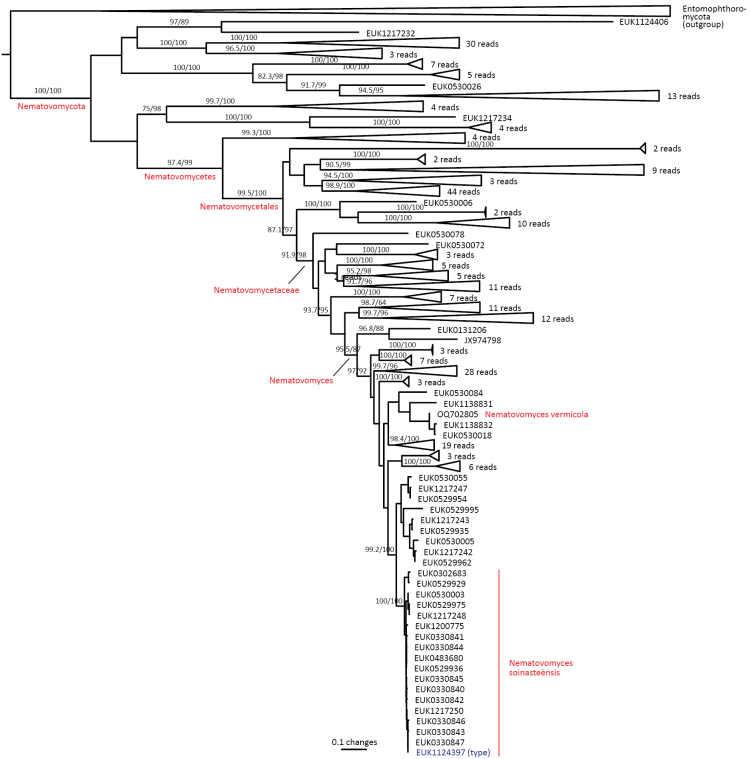
Maximum Likelihood SSU-ITS-LSU phylogram indicating the position of *Nematovomyces vermicola* and *N. soinasteënsis* within Nematovomycota, with ultra-rapid bootstrap values indicated. Other genus-level groups are collapsed. Entomophthoromycota spp. were used as an outgroup.

Separated from the vascular plant-associated species and algae-associated species of *Olpidium* s. stricto based on reticulate to spiky ornamentation in resting spores instead of star-like shapes. *Nematovomyces* spp. infect nematodes, rotifers, and their eggs. Distinguishable from other fungi based on diagnostic nucleotide signatures in SSU V4 (positions 729–743 aaccgggtgtggcct in *S. cerevisiae*; no mismatch allowed) and ITS2 (positions 68–77 aacaatgtct in *N. soinasteënsis*; one mismatch allowed) and LSU D2 (positions 679–698 in *N. soinasteënsis* and 595–614 in *S. cerevisiae*: gttgtctttgttattttcca; one mismatch allowed). Forms a monophyletic, least inclusive clade in Nematovomycetaceae, covering sequences OQ702947, GQ330624, OQ702883, JN054659, JN054675, OQ702805, EUK1100016, EUK1107129, EUK1102228, EUK1204135, EUK1124398, EUK1124395, EUK1124396, and EUK1124397 (Figs 1, 55).

##### Description.

Sporangia in the host cell singly or in rows, spherical or pyriform, 15–35 µm diam., with a smooth wall and curved exit tube. Zoospores spherical, 2.5–3.5 µm diam, with one posterior flagellum. Flagellum arched near the insertion point to the zoospore body. Thallus produces a single evacuation tube that leaves a narrow exit tube. Resting spores spherical or oblong, with surface ornamented by delicate reticular pattern or linear or branched spines, arranged in chains outside the animal cuticle or in culture. Infects nematodes, rotifers, and their eggs internally.

##### Notes.

Includes species parasitizing on nematodes, rotifers, and their eggs. Comprises about 50 potential species represented by sequences OQ702947 (peatland rotifer in MI, USA), GQ330624 (peatland water in Switzerland), OQ702883 (rotifer egg in lake water in ONT, Canada), JN054659 (activated sludge in NSW, Australia), JN054675 (activated sludge in Canada), EUK1100016 (permafrost in Canada), EUK1107129 (lake water in Sweden), EUK1102228 (forest soil in Puerto Rico), EUK1204135 (lake sediment in Lithuania), EUK1124398 (forest soil in Estonia), EUK1124395 (grassland soil in Estonia), and EUK1200775 (forest soil in Italy).

#### Nematovomyces vermicola

Taxon classificationFungiNematovomycetalesNematovomycetaceae

(G.L. Barron & Szuarto) Tedersoo & Esmaeilzadeh-Salestani comb. nov.

989CDAF5-1A78-5DB9-B37C-C5543904D488

859114

##### Basionym.

*Olpidium vermicola* G.L. Barron & Szuarto, Mycologia 78 (6): 972 (1986) [128304].

##### Diagnosis.

Separated from other species of *Nematovomyces* by echinulate resting spores and parasitism exclusively on nematode eggs.

##### Type.

Microscope slide OAC 10841 (**holotype**), rotting wood at Lake Manitowabing, Ontario, Canada, 45.5, –79.9; eDNA sequence OQ702805 (**legitype**) from the type locality.

##### Description.

As in Barron and Szuarto (1986).

##### Etymology.

*Nematoda* and *ovum* (Latin) refer to roundworms and their eggs, respectively, and describe the specific association with nematode eggs, indicating parasitic relationship with these structures.

##### Notes.

There are no ITS sequences or other eDNA sequences matching *N. vermicola*.

#### Nematovomyces

Taxon classificationFungiNematovomycetalesNematovomycetaceae

soinasteënsis Tedersoo sp. nov.

BD51D3A4-2BE2-537A-82E0-78190BC23410

Mycobank No: 859115

##### Diagnosis.

**Figure 56.**
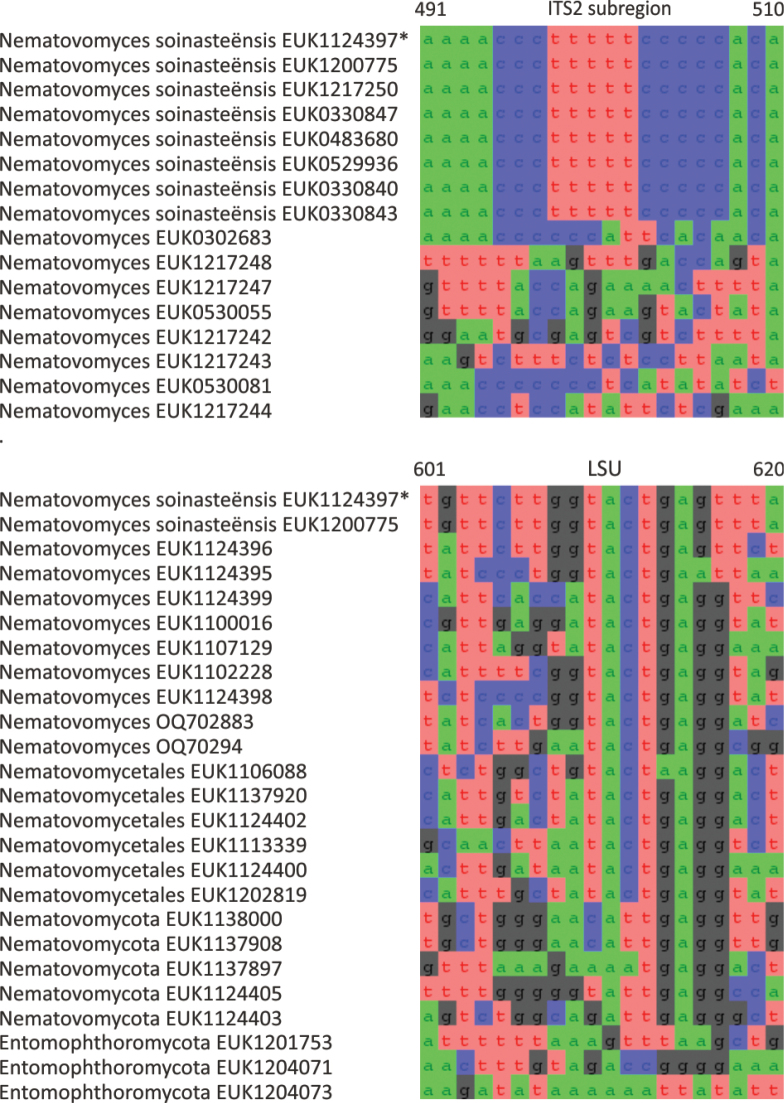
Diagnostic nucleotide sequences of *Nematovomyces soinasteënsis* relative to the closest related species in ITS2 and LSU. Numbers indicate positions in the legitype (marked with an asterisk).

Separation from other species of *Nematovomyces* based on ITS2 (positions 491–510 aaaaccctttttcccccaca; one mismatch allowed) and LSU (positions 601–620 tgttcttggtactgagttta; one mismatch allowed) as indicated in Fig. 56. Intraspecific variation up to 2.8% in ITS2 and up to 0.3% in LSU. Interspecific distance at least 8.0% in ITS2.

##### Type.

Vouchered soil sample TUE000860 (**holotype**); eDNA sequence EUK1124397 = OZ253814 (**legitype**); eDNA sample TUE100860 (**nucleotype**); GSMc plot S328, *Betula pendula* dominated forest in Soinaste, Estonia, 58.3322°N, 26.7678°E.

##### Description.

Other sequences: EUK1200775 (GSMc plot S1183, mixed forest soil in Aldino, Italy, 46.4072°N, 11.4964°E); EUK1217250 (GSMc plot G4679, *Salix triandra* swamp soil in Prangli Rivimaa, Estonia, 59.6151°N, 24.9871°E); EUK0330847 (GSMc plot S141, *Carpinus-Quercus-Alnus* forest soil in Shirgah, Iran, 36.2122°N, 52.8243°E); EUK0483680 (GSMc plot G4196, mixed forest soil in Kahvena, Estonia, 58.2799°N, 25.2316°E); EUK1217249 (GSMc plot G4800, *Ulmus-Alnus* temperate forest soil in Tuhkja, Estonia, 58.4159°N, 25.2327°E); EUK033840 (GSMc plot S939, tropical rainforest soil in Parotania, Bolivia, –17.5815, –66.3443°E); and EUK0330843 (GSMc plot G4030, *Quercus-Arbutus* forest soil in Ain Boumahdi, Morocco, 34.0096, –4.2858, 24.9871°E).

##### Etymology.

*Soinaste* (Estonian) refers to the type locality.

##### Notes.

Found in forest soils in Eurasia, North Africa, Central Asia, and South America (n = 18 records). The 16 additional GlobalFungi records indicate occurrence in soil and root samples across various ecosystems and biomes in Spain, China, and the USA.

#### Viljandiomycota

Taxon classificationFungiFungiMucoromycota

Tedersoo phyl. nov.

300045A0-CCFA-5CA5-9F9B-6FBA33BF5541

859115

##### Type class.

Viljandiomycetes Tedersoo.

##### Diagnosis.

Distinguishable from other fungi based on diagnostic nucleotide signatures in LSU D2 (positions 464–478 in type species and 490–504 in *S. cerevisiae* ctggccaacatcagt; one mismatch allowed). Forms a monophyletic, least inclusive clade in fungi, covering sequences EUK1699905, EUK1104555, EUK1124343, EUK1124346, EUK1104962, EUK1124344, EUK1202387, EUK1100361, EUK1201679, EUK1105441, EUK1124341, and EUK1124345 (Fig. 1).

##### Notes.

Recognized based on eDNA sequences only. Encoded as clade GS40 in EUKARYOME v1.9. Currently harbors Viljandiomycetes (class. nov.). Comprises 60–90 potential species. Detected in soil (98.2% out of 265 records), freshwater (0.7%), sediments (0.4%), and plant roots (0.4%) in high arctic to wet tropical biomes across all continents, including Antarctica.

#### Viljandiomycetes

Taxon classificationFungiMucoromycotaViljandiomycota

Tedersoo class. nov.

DC6147AF-12A7-51DA-B935-8A4435A43014

859117

##### Type order.

Viljandiales Tedersoo.

##### Diagnosis.

Distinguishable from other fungi based on a diagnostic nucleotide signature in LSU D2 (positions 464–478 in type species and 490–504 in *S. cerevisiae* ctggccaacatcagt, one mismatch allowed). Forms a monophyletic, least inclusive clade in Viljandiomycota, covering sequences EUK1699905, EUK1104555, EUK1124343, EUK1124346, EUK1104962, EUK1124344, EUK1202387, EUK1100361, EUK1201679, EUK1105441, EUK1124341, and EUK1124345 (Fig. 1).

##### Notes.

Recognized based on eDNA sequences only. Currently harbors Viljandiales (ord. nov.).

#### Viljandiales

Taxon classificationFungiViljandiomycotaViljandiomycetes

Tedersoo ord. nov.

96478BBA-FDF2-500A-9442-5145D82A76FC

859118

##### Type family.

Viljandiaceae Tedersoo.

##### Diagnosis.

Distinguishable from other fungi based on a diagnostic nucleotide signature in LSU D2 (positions 464–478 in type species and 490–504 in *S. cerevisiae* ctggccaacatcagt, one mismatch allowed). Forms a monophyletic, least inclusive clade in Viljandiomycetes, covering sequences EUK1699905, EUK1104555, EUK1124343, EUK1124346, EUK1104962, EUK1124344, EUK1202387, EUK1100361, EUK1201679, EUK1105441, EUK1124341, and EUK1124345 (Fig. 1).

##### Notes.

Recognized based on eDNA sequences only. Currently includes Viljandiaceae (fam. nov.) and several potentially family-level taxa represented by sequences EUK1699905 (forest soil in Ethiopia), EUK1104555 (forest soil in Sweden), EUK1124343 (wasteland soil in Estonia), EUK1124346 (urban soil in Estonia), EUK1104962 (forest soil in Puerto Rico), EUK1124344 (urban soil in Estonia), EUK1202387 (tundra soil in Finland), EUK1100361 (lake water in Sweden), and EUK1201679 (forest soil in Sweden).

#### Viljandiaceae

Taxon classificationFungiViljandiomycetesViljandiales

Tedersoo fam. nov.

45A0007C-DA43-544A-BFB4-9B0D76769315

859118

##### Type genus.

*Viljandia* Tedersoo.

##### Diagnosis.

Distinguishable from other fungi based on diagnostic nucleotide signatures in SSU V9 (positions 1695–1709 in *S. cerevisiae* gccagcaatggcagc; one mismatch allowed). Forms a monophyletic, least inclusive clade in Viljandiales, covering sequences EUK1105441, EUK1124341, EUK1124345, and EUK0524033 (Fig. 1).

##### Notes.

Recognized based on eDNA sequences only. Includes *Viljandia* (gen. nov.) and potentially another genus-level taxon represented by the sequence EUK0524033 (forest soil in India).

#### Viljandia

Taxon classificationFungiViljandialesViljandiaceae

Tedersoo gen. nov.

325473CB-7AED-52A8-A79A-36AA5398D88B

859120

##### Type species.

*Viljandia globalis* Tedersoo.

##### Diagnosis.

**Figure 57.**
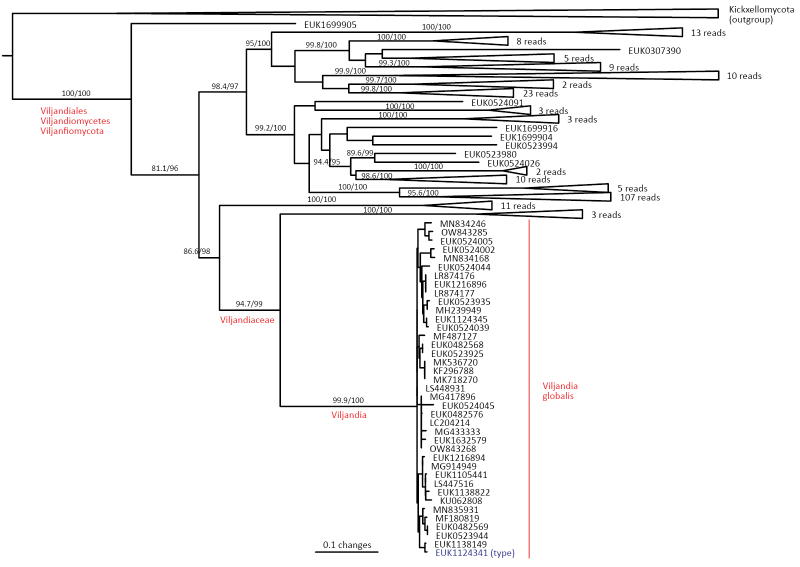
Maximum Likelihood SSU-ITS-LSU phylogram indicating the position of *Viljandia globalis* within Viljandiomycota, with ultra-rapid bootstrap values indicated (for higher-level classifications only). Other genus-level groups are collapsed. Kickxellomycota spp. were used as an outgroup.

Distinguishable from other species of Viljandiaceae based on diagnostic nucleotide signatures in SSU V9 (positions 1695–1709 in *S. cerevisiae* ggcttccggcagcca; one mismatch allowed) and 5.8S (positions 126–135 in the type species and 126–134 in *S. cerevisiae* cactctaagg; one mismatch allowed). Forms a monophyletic, least inclusive clade in Viljandiaceae, covering sequences EUK1105441, EUK1124341, and EUK1124345 (Figs 1, 57).

##### Notes.

Recognized based on eDNA sequences only. Currently comprises *Viljandia globalis* (sp. nov.).

#### Viljandia globalis

Taxon classificationFungiViljandialesViljandiaceae

Tedersoo sp. nov.

3AE386D8-257B-5AF8-A7DA-8D20FE40BC6C

859121

##### Diagnosis.

**Figure 58.**
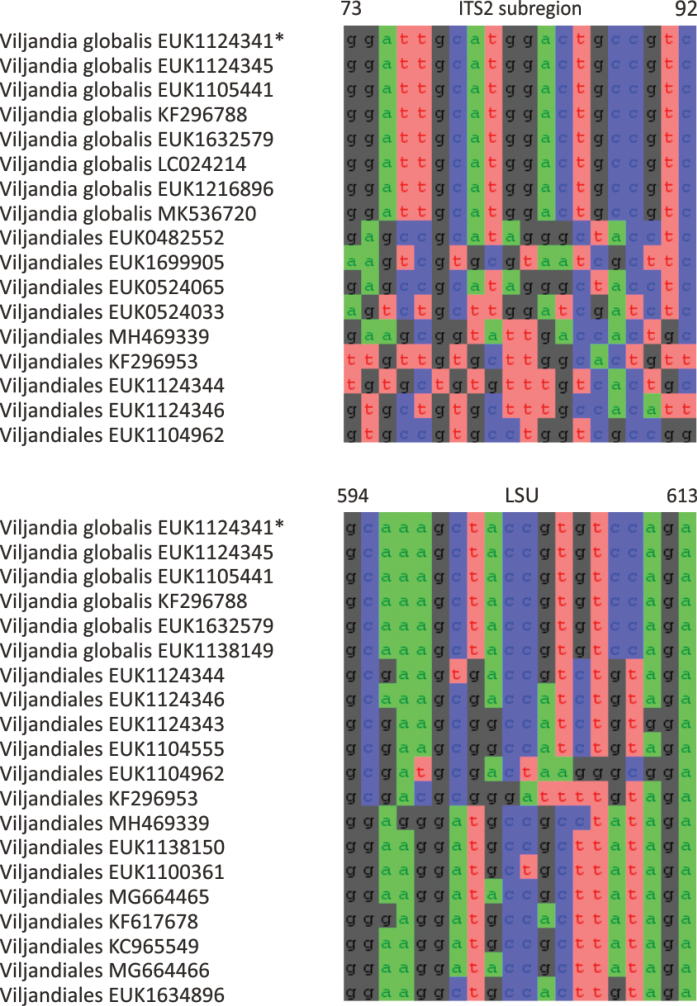
Diagnostic nucleotide sequences of *Viljandia globalis* relative to the closest related species in ITS2 and LSU. Numbers indicate positions in the legitype (marked with an asterisk).

Separation from other species of *Viljandia* based on ITS2 (positions 73–92 ggattgcatggactgccgtc; one mismatch allowed) and LSU (positions 594–613 gcaaagctaccgtgtccaga; one mismatch allowed) as indicated in Fig. 58. Intraspecific variation up to 7.5% in ITS2 and up to 2.2% in LSU. Interspecific distance > 20% in ITS2.

##### Type.

Vouchered soil sample TUE028497 (**holotype**); eDNA sequence EUK1124341 = OZ253815 (**legitype**); eDNA sample TUE128497 (**nucleotype**); GSMc plot G5902, irrigated stadium lawn in Viljandi, Estonia, 58.3611°N, 25.6068°E.

##### Description.

Other sequences: EUK1632579 (GSMc plot G4506, woodland soil in Terikeste Hiiepärna, Estonia, 58.2972°N, 27.0681°E); LC204214 (*Picea crassifolia* temperate forest soil in Inner Mongolia, China, 38.77°N, 105.89°E); EUK1124345 (GSMc plot G5901, *Aesculus hippocastanum* alley soil in Tartu, Estonia, 58.3676°N, 26.7255°E); EUK1105441 (boreal coniferous forest soil near Hofors, Sweden, 60.49°N, 16.3°E); EUK1216896 (GSMc plot G4796, *Acer platanoides* forest soil in Alavere, Estonia, 58.7562°N, 26.5109°E); KF296788 (tundra soil in Prince Patrick Island, Canada, 76.23, –119.3); and MK536720 (soil crust in Victoria Land, Antarctica).

##### Etymology.

*Viljandi* (Estonian) refers to the type locality, and *globus* (Latin) refers to the globe, reflecting the cosmopolitan distribution.

##### Notes.

Distributed in soil worldwide, including Antarctica (n = 39 records). The 119 additional GlobalFungi records support these findings.

#### Waitukubulimycota

Taxon classificationFungiFungiMucoromycota

Tedersoo phyl. nov.

A5ABFD10-E4C2-5B09-BE9E-7F45767127DB

859122

##### Type class.

Waitukubulimycetes Tedersoo.

##### Diagnosis.

Distinguishable from other fungi based on a diagnostic nucleotide signature in the LSU 5’ end (positions 52–66 in the type species and *S. cerevisiae* tggaggaaaagaaaa, no mismatch allowed). Forms a monophyletic, least inclusive clade in fungi, covering sequences EUK1120710, EUK1173015, EUK1186290, EUK1186291, and EUK1186292 (Fig. 1).

##### Notes.

Recognized based on eDNA sequences only. Not encoded specifically in EUKARYOME v1.9. Waitukubulimycota currently harbors the single class Waitukubulimycetes. Waitukubulimycota comprises five species. Members of this phylum have been detected in soil (100% out of seven records) in arctic to wet tropical biomes across all continents, excluding Antarctica.

#### Waitukubulimycetes

Taxon classificationFungiMucoromycotaWaitukubulimycota

Tedersoo class. nov.

C07D24BE-95A3-5165-8B78-2A71BAFC7686

859123

##### Type order.

Viljandiales Tedersoo.

##### Diagnosis.

Distinguishable from other fungi based on a diagnostic nucleotide signature in the LSU 5’ end (positions 52–66 in the type species and *S. cerevisiae* tggaggaaaagaaaa, no mismatch allowed). Forms a monophyletic, least inclusive clade in Waitukubulimycota, covering sequences EUK1120710, EUK1173015, EUK1186290, EUK1186291, and EUK1186292 (Fig. 1).

##### Notes.

Recognized based on eDNA sequences only. Waitukubulimycetes currently harbors Waitukubulimycetales.

#### Waitukubulimycetales

Taxon classificationFungiWaitukubulimycotaWaitukubulimycetes

Tedersoo ord. nov.

761C2DF9-5529-59EB-B171-F2092D1BCEC5

859124

##### Type family.

Waitukubulimycetaceae Tedersoo.

##### Diagnosis.

Distinguishable from other fungi based on diagnostic nucleotide signatures in LSU 5’ end (positions 52–66 in type species and *S. cerevisiae* tggaggaaaagaaaa, no mismatch allowed) and LSU D2 (positions 246–262 in type species and 240–256 in *S. cerevisiae* tgtgttcrctctgtgat; two mismatches allowed). Forms a monophyletic, least inclusive clade in Waitukubulimycetes, covering sequences EUK1120710, EUK1173015, EUK1186290, EUK1186291, and EUK1186292.

##### Notes.

Recognized based on eDNA sequences only. Currently includes Waitukubulimycetaceae (fam. nov.).

#### Waitukubulimycetaceae

Taxon classificationFungiWaitukubulimycetesWaitukubulimycetales

Tedersoo fam. nov.

4D06A584-1D52-5AA2-92C7-15D77204B61B

859125

##### Type genus.

*Waitukubulimyces* Tedersoo.

##### Diagnosis.

Distinguishable from other fungi based on diagnostic nucleotide signatures in LSU 5’ end (positions 52–66 in type species and *S. cerevisiae* tggaggaaaagaaaa, no mismatch allowed) and LSU D2 (positions 246–262 in type species and 240–256 in *S. cerevisiae* tgtgttcrctctgtgat; one mismatch allowed) and ITS2 (positions 129–138 in type species tgggtcactt; one mismatch allowed). Forms a monophyletic, least inclusive clade in Waitukubulimycetales, covering sequences EUK1120710, EUK1173015, EUK1186290, EUK1186291, and EUK1186292 (Fig. 1).

##### Notes.

Recognized based on eDNA sequences only. Currently comprises *Waitukubulimyces* (gen. nov.).

#### Waitukubulimyces

Taxon classificationFungiWaitukubulimycetalesWaitukubulimycetaceae

Tedersoo gen. nov.

AECA1819-64F2-519B-A850-C302EA439ED4

859126

##### Type species.

*Waitukubulimyces cliftonii* Tedersoo.

##### Diagnosis.

**Figure 59.**
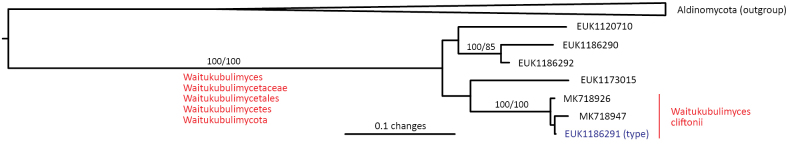
Maximum Likelihood SSU-ITS-LSU phylogram indicating the position of *Waitukubulimyces cliftonii* within Waitukubulimycota, with ultra-rapid bootstrap values indicated (for higher-level classifications only). Other genus-level groups are collapsed. Aldinomycota spp. were used as an outgroup.

Distinguishable from other fungi based on diagnostic nucleotide signatures in LSU 5’ end (positions 52–66 in type species and *S. cerevisiae* tggaggaaaagaaaa, no mismatch allowed), LSU D2 (positions 246–262 in type species and 240–256 in *S. cerevisiae* tgtgttcrctctgtgat; one mismatch allowed), and ITS2 (positions 129–138 in type species tgggtcactt; one mismatch allowed). Forms a monophyletic, least inclusive clade in Waitukubulimycetaceae, covering sequences EUK1120710, EUK1173015, EUK1186290, EUK1186291, and EUK1186292 (Figs 1, 59).

##### Notes.

Recognized based on eDNA sequences only. Comprises five potential species represented by sequences EUK1120710 (botanical garden soil in Estonia), EUK1173015 (forest soil in China), and EUK1186290 and EUK1186292 (both forest soil in Puerto Rico).

#### Waitukubulimyces cliftonii

Taxon classificationFungiWaitukubulimycetalesWaitukubulimycetaceae

Tedersoo sp. nov.

ED3F6019-3F2E-59F3-B870-7B96AAA54785

859127

##### Diagnosis.

**Figure 60.**
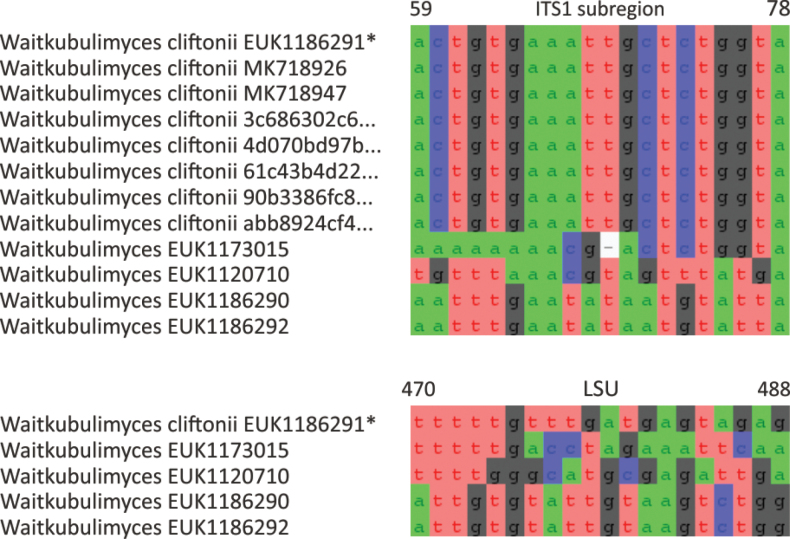
Diagnostic nucleotide sequences of *Waitukubulimyces cliftonii* relative to the closest related species in ITS2 and LSU. Numbers indicate positions in the legitype (marked with an asterisk).

Separation from other species of *Waitukubulimyces* based on ITS1 (positions 59–78 actgtgaaattgctctggta; one mismatch allowed) and LSU (positions 470–489 tttttgtttgatgagtagag; one mismatch allowed) as indicated in Fig. 60. Intraspecific variation up to 5.3% in ITS1. Interspecific distance > 20% in ITS1.

##### Type.

Vouchered soil sample TUE002020 (**holotype**); eDNA sequence EUK1186291 = OZ253816 (**legitype**); eDNA sample TUE102020 (**nucleotype**); GSMc plot G5043, tropical rainforest in Bellevue Chopin, Dominica, 15.2567, –61.3428°E.

##### Description.

Other sequences: MK718926 and MK718947 (both: barren soil in CO, USA); GlobalFungi records 3c686302c6bfd00ff4db5b414d28c645 (woodland soil in Marina, CA, USA, 36.6849, –121.7780°E); 4d070bd97b6d68091a85749c15e6c744 (forest soil in Soria, Spain, 41.8694, –2.87528°E); 61c43b4d22e1a0efa38d2dba3311970e (cropland soil in Hangle, Uyghuria, China, 46.1886°N, 83.3294°E); 90b3386fc8d47acc87e18126f1c5e50b (near-glacier soil in Arikaree, CO, USA); and abb8924cf48b17d892b88816e96f0ff0 (grassland soil in Yahelong Gongma, Tibet, 38.21°N, 98.16°E).

##### Etymology.

*>Waitukubuli>* (Igneri) refers to the country of *Dominica*, where the type material was collected, and Clifton refers to Clifton P. Bueno de Mesquita, who collected the first material of this species (MK718926 and MK718947; Bueno de Mesquita et al. 2020).

##### Notes.

Recorded from soil in three localities in Dominica and the USA. The 11 additional GlobalFungi records supplement findings from soil in various habitats in Spain, China, Tunisia, and the USA. ITS1 was used in molecular diagnosis instead of ITS2 because only a single sequence was available for ITS2.

#### Tartumyceta

Taxon classificationFungiFungiMucoromycota

Tedersoo subreg. nov.

95333BF5-9D23-564A-8485-1B74ED5E7593

859128

##### Type phylum.

Tartumycota Tedersoo.

##### Diagnosis.

Distinguishable from other fungi based on diagnostic nucleotide signatures in LSU D3 (positions 1009–1023 in type species and 969–983 in *S. cerevisiae* ggaacttgtacagtt, no mismatch allowed). Forms a monophyletic, least inclusive clade in fungi, covering sequences OQ702815, EUK1186161, OQ687331, EUK1186165, EUK1186157, EUK1200073, EUK1186162, EUK1123648, EUK1138300, OQ702816, ON754309, UDB028835, and HQ191300 (Fig. 1).

##### Notes.

Recognized as a subkingdom due to its sister position to all remaining fungi. Currently harbors Tartumycota (phyl. nov.).

#### Tartumycota

Taxon classificationFungiFungiMucoromycota

Tedersoo phyl. nov.

D0475B58-916D-5516-A0EA-A122F9DA3B69

859129

##### Type class.

Tartumycetes Tedersoo.

##### Diagnosis.

Distinguishable from other fungi based on a diagnostic nucleotide signature in LSU D3 (positions 1009–1023 in the type species and 969–983 in *S. cerevisiae* ggaacttgtacagtt, no mismatch allowed). Forms a monophyletic, least inclusive clade in fungi, covering sequences OQ702815, EUK1186161, OQ687331, EUK1186165, EUK1186157, EUK1200073, EUK1186162, EUK1123648, EUK1138300, OQ702816, ON754309, UDB028835, and HQ191300 (Fig. 1).

##### Notes.

Recognized based on eDNA and single-cell sequences only. Encoded as “freshol1” and clade BCG2 in previous studies and EUKARYOME v1.9. Currently harbors Tartumycetes (class. nov.) and potentially a class-level group represented by sequences OQ687331 (lake water in MI, USA), OQ702815 (algal sample in MI, USA), EUK1186161, and EUK1186165 (both rotting algae in Estonia). Comprises potentially 100–110 species. Detected in soil (64.9% out of 296 records), water (22.0%), sediments (8.8%), and algae (4.4%) in tundra to hot tropical biomes across all continents except Antarctica. Microscopic analyses of freshwater algae suggest parasitic interactions. It is possible that Tartumycota spp. are parasitic on soil and aquatic algae.

#### Tartumycetes

Taxon classificationFungiMucoromycotaTartumycota

Tedersoo class. nov.

0E6A3AF0-B186-574E-AFC3-CB2585907523

859130

##### Type order.

Tartumycetales Tedersoo.

##### Diagnosis.

Distinguishable from other fungi based on a diagnostic nucleotide signature in LSU D4 (positions 1439–1453 in type species and 1404–1418 in *S. cerevisiae* gatgccgcgtcgaac, one mismatch allowed). Forms a monophyletic, least inclusive clade in Tartumycota, covering sequences EUK1186157, EUK1200073, EUK1186162, EUK1123648, EUK1138300, OQ702816, ON754309, UDB028835 and HQ191300 (Fig. 1).

##### Notes.

Recognized based on eDNA and single-cell sequences only. Currently harbors Tartumycetales (ord. nov.).

#### Tartumycetales

Taxon classificationFungiTartumycotaTartumycetes

Tedersoo ord. nov.

082BFA71-99D4-50DE-AA6C-AF8F0A69374A

859131

##### Type family.

Tartumycetaceae Tedersoo.

##### Diagnosis.

Distinguishable from other fungi based on diagnostic nucleotide signatures in ITS2 (positions 120–129 in type species gaaccaaagg, one mismatch allowed) and LSU D1 (positions 164–178 in type species and 161–175 in *S. cerevisiae* gatgcctgtgggagc, one mismatch allowed). Forms a monophyletic, least inclusive clade in Tartumycetes, covering sequences EUK1186157, EUK1200073, EUK1186162, EUK1123648, EUK1138300, OQ702816, ON754309, UDB028835, and HQ191300 (Fig. 1).

##### Notes.

Recognized based on eDNA sequences only. Currently includes Tartumycetaceae and several potentially family-level groups represented by sequences EUK1186157 (forest soil in Puerto Rico), EUK1200073 (tundra soil in Finland), EUK1186162 (rotting algal sample in Estonia), OQ702816 (algal sample in MI, USA), ON754309 (river sediment in China), UDB028835 (lake water in Germany), and HQ191300 (lake water in France).

#### Tartumycetaceae

Taxon classificationFungiTartumycetesTartumycetales

Tedersoo fam. nov.

E8A6F217-9965-5ACE-93B4-23E828402B65

859132

##### Type genus.

*Tartumyces* Tedersoo.

##### Diagnosis.

Distinguishable from other fungi based on diagnostic nucleotide signatures in ITS2 (positions 173–187 in type species ggaaagcgtagtagg, two mismatches allowed) and LSU D4 (positions 1439–1453 in type species and 1404–1418 in *S. cerevisiae* gatgccgcgtcgaac, one mismatch allowed). Forms a monophyletic, least inclusive clade in Tartumycetales, covering sequences EUK1123648, EUK1138300, EUK1186160, EUK1186168, and EUK1186172 (Fig. 1).

##### Notes.

Recognized based on eDNA sequences only. Includes *Tartumyces* (gen. nov.) and several potentially genus-level taxa represented by sequences EUK1186160 (forest soil in Dominica), EUK1186168 (forest soil in Udmurtia), and EUK1186172 (forest soil in Italy).

#### Tartumyces

Taxon classificationFungiTartumycetalesTartumycetaceae

Tedersoo gen. nov.

00ECABB6-6B0C-5FF0-A962-9C819B8D84CF

859134

##### Type species.

*Tartumyces setoi* Tedersoo.

##### Diagnosis.

**Figure 61.**
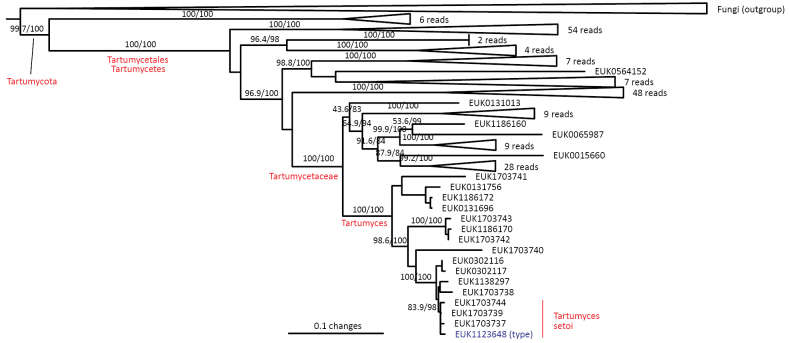
Maximum Likelihood SSU-ITS-LSU phylogram indicating the position of *Tartumyces setoi* within Tartumycota, with ultra-rapid bootstrap values indicated. Other genus-level groups are collapsed. Members of various fungal phyla were used as an outgroup.

Distinguishable from other fungi based on diagnostic nucleotide signatures in ITS2 (positions 289–300 in type species gggtttgcaaac, one mismatch allowed) and LSU D4 (positions 624–633 in type species and 601–610 in *S. cerevisiae* gaatttattc, one mismatch allowed). Forms a monophyletic, least inclusive clade in Tartumycetaceae, covering sequences EUK1123648 and EUK1138300 (Figs 1, 61).

##### Notes.

Recognized based on eDNA sequences only. Comprises around 10 potential species.

#### Tartumyces setoi

Taxon classificationFungiTartumycetalesTartumycetaceae

Tedersoo sp. nov.

628A60FC-B505-5A1D-867B-4E2A35FE1DD4

859136

##### Diagnosis.

**Figure 62.**
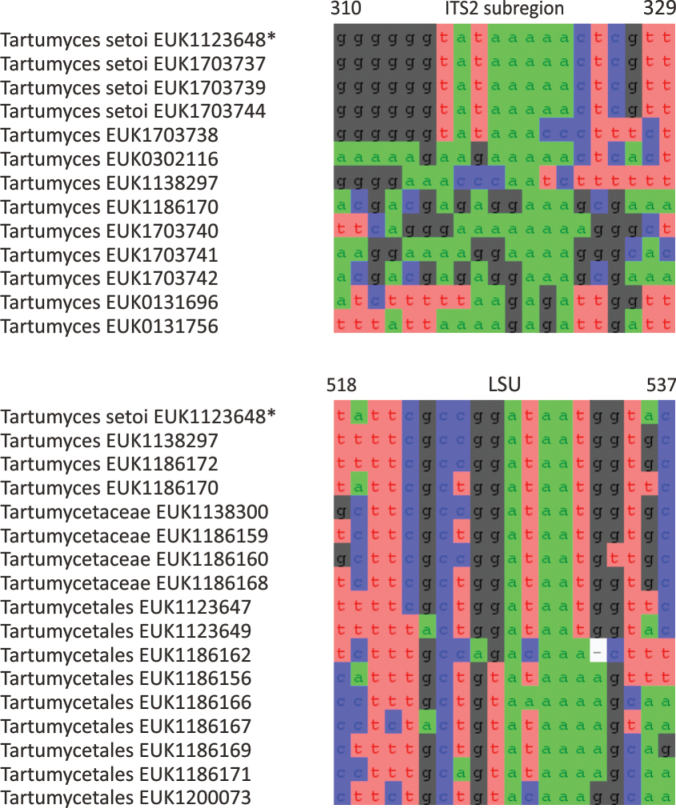
Diagnostic nucleotide sequences of *Tartumyces setoi* relative to the closest related species in ITS2 and LSU. Numbers indicate positions in the legitype (marked with an asterisk).

Separation from other species of *Tartumyces* based on ITS2 (positions 310–329 ggggggtataaaaactcgtt; one mismatch allowed) and LSU D2 (positions 518–539 tattcgccggataatggtac; no mismatch allowed) as indicated in Fig. 62. Intraspecific variation up to 2.8% in ITS2. Interspecific distance at least 4.5% in ITS2.

##### Type.

Vouchered soil sample TUE002210 (**holotype**); eDNA sequence EUK1123648 = OZ253817 (**legitype**); eDNA sample TUE102210 (**nucleotype**); GSMc plot G5233, wasteland in Tartu, Estonia, 58.3972°N, 26.7693°E.

##### Description.

Other sequences: EUK1703744 (GSMc plot G4372, mixed forest soil in Kiisli, Estonia, 58.6955°N, 26.9128°E); EUK1703739 (GSMc plot G3569, *Quercus robur* park soil in Äksi, Estonia, 58.5290°N, 26.6385°E); and EUK1703737 (GSMc plot G3413, *Salix caprea* forest soil in Väägvere, Estonia, 58.4389°N, 26.8976°E).

##### Etymology.

*>Tartu* (Estonian) refers to the city and county in Estonia, where the type material and most other specimens were collected. The epithet refers to Kensuke Seto, the first to obtain coarse single-cell photographs of species belonging to this phylum (Seto et al. 2023).

##### Notes.

Found in soil in Estonia (n = 4 records). An additional record in GlobalFungi also indicates occurrence in Estonian plantation soil.

## ﻿Conclusion

By integrating long-read sequences, DNA-based taxonomy, and phylogenetics, we formally describe species and corresponding higher taxa of the most common previously unrecognized fungal lineages. These potentially unculturable groups add roughly one-third to the known large-scale phylogenetic diversity of fungi, yet contribute to < 5% of the described and expected fungal species richness. Our analysis sheds light on the strong contribution of taxonomic dark matter to the fungal tree of life and provides a simple means for its detection and communication. Ultimately, our findings highlight the necessity for a transformative approach in fungal taxonomy that integrates rapidly advancing molecular data to capture the vast extent of fungal diversity more accurately and reproducibly. We also advocate a broader use of fluorescence-activated single-cell capture and sequencing of fungi for concomitant analysis of taxonomically and functionally important genes to understand their basic lifestyle features and obtain hints for their cultivation and visualization.

## ﻿Additional information

### Use of AI

No use of AI was reported.

### Author ORCIDs

Victoria Prins https://orcid.org/0009-0003-8968-6773

Vladimir Mikryukov https://orcid.org/0000-0003-2786-2690

Mohammad Bahram https://orcid.org/0000-0002-9539-3307

Kessy Abarenkov https://orcid.org/0000-0001-5526-4845

Keyvan Esmaeilzadeh-Salestani https://orcid.org/0000-0002-6882-7616

Julia Pawłowska https://orcid.org/0000-0003-4914-5182

Christian Wurzbacher https://orcid.org/0000-0001-7418-0831

Saad Hussin Alkahtani https://orcid.org/0000-0001-7381-5110

R. Henrik Nilsson https://orcid.org/0000-0002-8052-0107

### Data availability

All materials and data are publicly available as follows: material and eDNA samples in the TUE repository; DNA sequences in Suppl. material 3, EUKARYOME and UNITE databases, with legitypes in the European Nucleotide Archive (accessions OZ253786-OZ253832); multiple sequence alignments in the EUKARYOME homepage.

## Supplementary materials

Supplementary material 1 Maximum Likelihood SSU-5.8S-LSU phylogram Data type pdf Explanation note Maximum Likelihood SSU-5.8S-LSU phylogram showing phylogenetic placement of previously unrecognized fungal lineages among fungi, with ultra-rapid bootstrap values indicated. Low support values < 95/<99% and support values within genera are not indicated. Species of Holozoa and Nucleariae were used as an outgroup. https://binary.pensoft.net/file/1444254This dataset is made available under the Open Database License (http://opendatacommons.org/licenses/odbl/1.0/). The Open Database License (ODbL) is a license agreement intended to allow users to freely share, modify, and use this Dataset while maintaining this same freedom for others, provided that the original source and author(s) are credited.Leho Tedersoo, Mahdieh S. Hosseyni Moghadam, Kristel Panksep, Victoria Prins, Sten Anslan, Vladimir Mikryukov, Mohammad Bahram, Kessy Abarenkov, Urmas Kõljalg, Keyvan Esmaeilzadeh-Salestani, Julia Pawłowska, Christian Wurzbacher, Yi Ding, Saad Hussin Alkahtani, R. Henrik Nilsson

Supplementary material 2 Updated taxonomy of fungi, including newly described taxa and provisional higher-ranking taxa based on phylogenetic analyses Data type xlsx https://binary.pensoft.net/file/1444255This dataset is made available under the Open Database License (http://opendatacommons.org/licenses/odbl/1.0/). The Open Database License (ODbL) is a license agreement intended to allow users to freely share, modify, and use this Dataset while maintaining this same freedom for others, provided that the original source and author(s) are credited.Leho Tedersoo, Mahdieh S. Hosseyni Moghadam, Kristel Panksep, Victoria Prins, Sten Anslan, Vladimir Mikryukov, Mohammad Bahram, Kessy Abarenkov, Urmas Kõljalg, Keyvan Esmaeilzadeh-Salestani, Julia Pawłowska, Christian Wurzbacher, Yi Ding, Saad Hussin Alkahtani, R. Henrik Nilsson

Supplementary material 3 DNA sequences and their metadata used for phylogenetic analyses, taxon descriptions and delimitation Data type xlsx https://binary.pensoft.net/file/1444256This dataset is made available under the Open Database License (http://opendatacommons.org/licenses/odbl/1.0/). The Open Database License (ODbL) is a license agreement intended to allow users to freely share, modify, and use this Dataset while maintaining this same freedom for others, provided that the original source and author(s) are credited.Leho Tedersoo, Mahdieh S. Hosseyni Moghadam, Kristel Panksep, Victoria Prins, Sten Anslan, Vladimir Mikryukov, Mohammad Bahram, Kessy Abarenkov, Urmas Kõljalg, Keyvan Esmaeilzadeh-Salestani, Julia Pawłowska, Christian Wurzbacher, Yi Ding, Saad Hussin Alkahtani, R. Henrik Nilsson
