# Supplementary material for: ﻿Thirty novel fungal lineages: formal description based on environmental samples and DNA
Source: MycoKeys. 2025 Oct 20;124:1–121. doi: 10.3897/mycokeys.124.161674 (PMC12559954; doi:10.3897/mycokeys.124.161674)
Supplement: Supplementary material 1 — Maximum Likelihood SSU-5.8S-LSU phylogram [file mycokeys-124-001-s001.pdf]

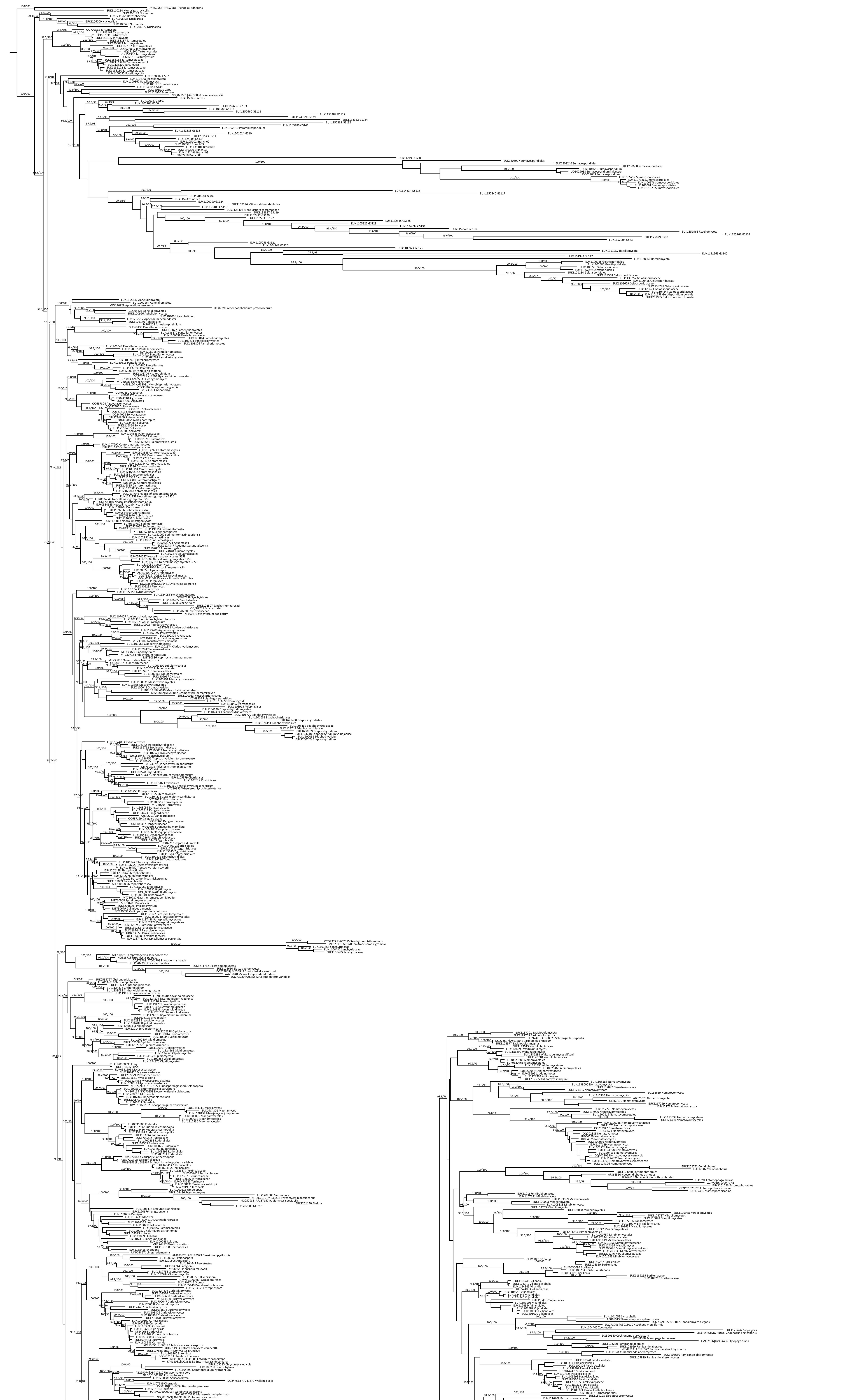

Figure S1. Maximum Likelihood SSU-5.8S-LSU phylogram showing phylogenetic placement of previously unrecognised fungal lineages among fungi, with ultra-rapid bootstrap values indicated. Low support values <95/<99% and support values within genera are not indicated. Species of Holozoa and Nucleariae were used as an outgroup.
